# SMOOT libraries and phage-induced directed evolution of Cas9 to engineer reduced off-target activity

**DOI:** 10.1371/journal.pone.0231716

**Published:** 2020-04-16

**Authors:** Derek Cerchione, Katherine Loveluck, Eric L. Tillotson, Fred Harbinski, Jen DaSilva, Chase P. Kelley, Elise Keston-Smith, Cecilia A. Fernandez, Vic E. Myer, Hariharan Jayaram, Barrett E. Steinberg

**Affiliations:** Editas Medicine, Cambridge, Massachusetts, United States of America; New England Biolabs Inc, UNITED STATES

## Abstract

RNA-guided endonucleases such as Cas9 provide efficient on-target genome editing in cells but may also cleave at off-target loci throughout the genome. Engineered variants of *Streptococcus pyogenes* Cas9 (SpCas9) have been developed to globally reduce off-target activity, but individual off-targets may remain, or on-target activity may be compromised. In order to evolve against activity at specific off-targets while maintaining strong on-target editing, we developed a novel M13 bacteriophage-mediated selection method. Using this method, sequential rounds of positive and negative selection are used to identify mutations to Cas9 that enhance or diminish editing activity at particular genomic sequences. We also introduce scanning mutagenesis of oligo-directed targets (SMOOT), a comprehensive mutagenesis method to create highly diverse libraries of Cas9 variants that can be challenged with phage-based selection. Our platform identifies novel SpCas9 mutants which mitigate cleavage against off-targets both in biochemical assays and in T-cells while maintaining higher on-target activity than previously described variants. We describe an evolved variant, *S*. *pyogenes* Adapted to Reduce Target Ambiguity Cas9 (SpartaCas), composed of the most enriched mutations, each of unknown function. This evolved Cas9 mutant reduces off-target cleavage while preserving efficient editing at multiple therapeutically relevant targets. Directed evolution of Cas9 using our system demonstrates an improved structure-independent methodology to effectively engineer nuclease activity.

## Introduction

Cas9 and other RNA-guided nucleases have been engineered to be robust tools for gene editing[1,2]. However, Cas9 activity can tolerate mismatches between the guide RNA (gRNA) and the target DNA sequence, permitting undesirable editing at off-target sites[3]. Off-target cleavage events can pose potential safety risks in therapeutic applications and complicate gene editing experiments. Current approaches to prevent off-target editing include screening targets with minimal sequence homology to other sites in the genome[4] or employing computational models to predict and avoid likely off-targets[5]. However, current models are inadequate to comprehensively predict off-targets[6], and Cas9 has been shown to cleave at off-targets with five or more mismatched bases[7], making avoidance difficult. Additionally, the choice of target sites may be limited for applications when cleavage at a particular nucleotide or motif is required or when editing loci with sparse protospacer-adjacent motif (PAM) sites.

Several high-fidelity Cas9 variants have been rationally mutated to alleviate off-target cleavage[8,9]. However, previous studies using these variants have indicated that their activities at the intended target are sharply reduced at specific loci. Additionally, certain off-target events may remain after rational engineering. If a particular gRNA retains a problematic off-target profile, an engineering strategy tailored to eliminate specific off-targets may be needed. Finally, as the gene editing toolkit continues to rapidly expand[10], structure-independent methods to strengthen nuclease fidelity are advantageous. We therefore desired a method to engineer increased fidelity without mitigating on-target cleavage activity, especially when a particular gRNA is required (eg. to disrupt a splice site[11]).

While rational design often neglects beneficial mutations outside domains of interest, directed evolution provides a naïve strategy for engineering nuclease activity. For a specific gRNA, directed evolution enables a dual approach of selecting against particular off-targets while also selecting for on-target function. We demonstrate a novel library generation method to comprehensively mutate a gene of interest, and we apply this method to exhaustively search the sequence space of *Streptococcus pyogenes* Cas9 (SpCas9). We further build a competitive selection scheme to screen these libraries using filamentous bacteriophage, which continually challenge the mutants to strengthen enrichment of the fittest variants. The combination of these approaches establishes a directed evolution platform which allows us to select a diverse library of Cas9 mutants for desired nuclease characteristics.

## Results

### SMOOT libraries exhibit comprehensive mutagenesis across Cas9 codon positions

Success of directed evolution experiments is limited by the diversity of the input library. In order to access the most diverse possible libraries of SpCas9, we strove to create a library which included every possible single amino acid mutation. Established methods, such as error-prone PCR, are often biased and probabilistically favor single-base-pair mutations within codons[12], which drastically reduces the number of potential amino acid mutations. Several techniques exist to create comprehensive saturation libraries[13]; however, these techniques are often labor-intensive and require separate reactions to generate mutations spanning the region of interest[14]. Other techniques allow for pooled generation of libraries, but either require particular sequence sites[15] or complex chemistries and thus more laborious library creation[16]. These restrictions create time-consuming bottlenecks and sequence-dependent limitations in library preparation.

**Fig 1:**
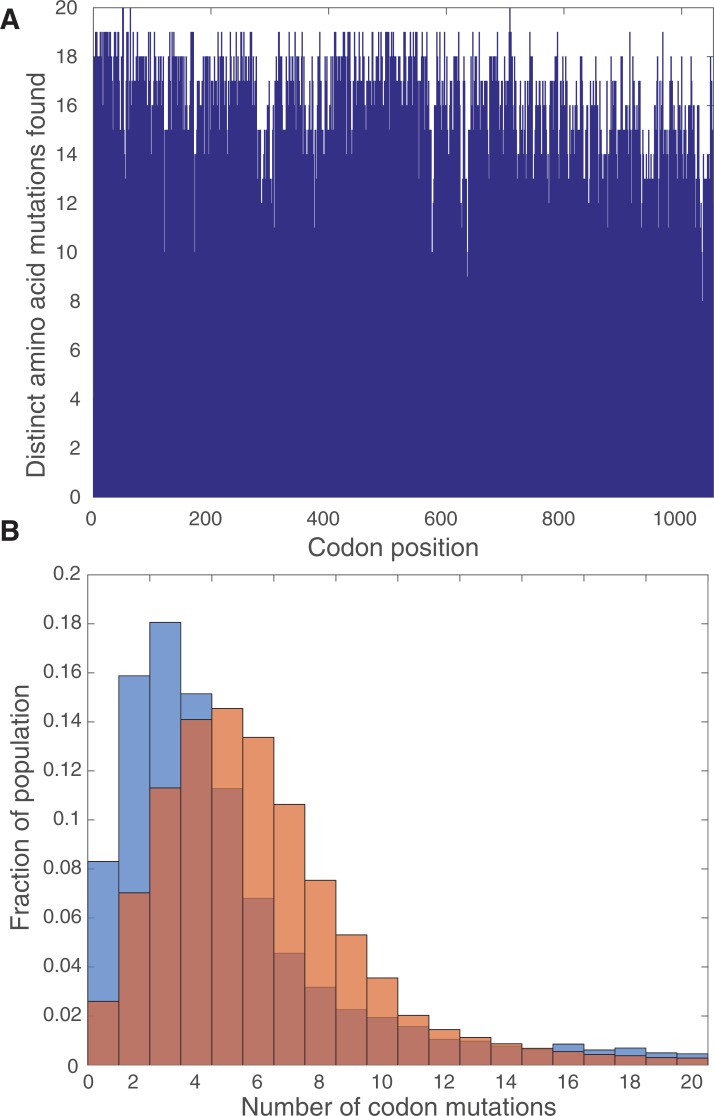
SMOOT can mutate all codons in a gene at a tunable mutation rate. (A) SMOOT optimization was originally performed on SaCas9 before being applied to SpCas9. Distribution of distinct nonsynonymous mutations produced by SMOOT throughout the Cas9 sequence, as identified by PacBio long-read sequencing and pairwise sequence alignment and custom analysis via Matlab. Each distribution represents data from a single library. (B) Probability density function of number of codon mutations per mutant allele. The orange distribution was generated from a mutant library with no forward tuning primer added, while the blue distribution was generated from a library with 0.5 nM forward tuning primer added. Overlap of distributions is shown by intermediate color.

To address these issues, we developed scanning mutagenesis of oligo-directed targets (SMOOT), a novel technique which simplifies library creation and generates a comprehensive and highly diverse library of codon substitutions in a one-step reaction that can be performed in a single day. SMOOT uses common reagents and a simple plasmid template without uracil- or sequence-dependence, making it universally applicable to any protein-coding gene. The SMOOT protocol was first optimized for mutagenizing *S*. *aureus* Cas9. (Fig 1 and S1 Fig). Phosphorylated oligonucleotides designed to incorporate mutagenic codons into the sequence of interest were first pooled. A reverse primer is designed distal to the mutagenic region (S4 Table). The template plasmid, forward mutagenic primers, and reverse primers are combined in a PCR-like reaction with Phusion high-fidelity polymerase over multiple cycles with long extension times(S5 Table). The SMOOT reaction resembles overlap extension (SoE), but the eventual polymerized oligos form multiply nicked circular plasmids (nicked on both strands), amongst other products. The mutation rate of the reaction can further be tuned by spiking in a forward primer upstream of the mutagenic region, likely by weak strand displacement activity of the Phusion polymerase (Fig 1B). After cycling, unwanted reaction byproducts are digested with a combination of DpnI (to digest template plasmid), Lambda Exonuclease (to digest linear DNA with incorporated phosphate groups), and Exo I (to digest primers and other remaining ssDNA). Finally, the reaction is transformed into electrocompetent *Escherichia coli*. Typical transformation efficiencies result in around 10^6^ CFU, allowing large library sizes. The SMOOT reaction can be completed in a single day from any circular DNA template.

To test SMOOT, we applied SMOOT to SaCas9 and successfully mutated every amino acid within the Cas9 protein to nearly every other amino acid (Fig 1). Furthermore, by isolating the enriched mutant plasmids at the end of a selection, we can perform successive rounds of SMOOT from selected libraries. Importantly, this method is not limited to mutagenesis of specific domains in the Cas9 protein, but rather can be applied to survey mutations across all codons in the gene. Finally, by titrating in the tuning primer, we observe a shift in the distribution of number of mutations incorporated into each library molecule (Fig 1B). The library size afforded by SMOOT allows for coverage of all single amino acid substitutions throughout the mutagenized protein while sampling broadly among double, triple and other higher-order combinations of mutations. SMOOT can therefore quickly and easily create a comprehensive saturation mutagenesis library while incorporating additional diversity in the library pool.

### Phage-based selection induces competition for desired nuclease activity

In order to pan the library of Cas9 variants, we developed a high-throughput selection process for SpCas9. Most existing methods to evolve nucleases are better described as screens rather than competitive selections, as they select for a single cleavage event[17]. We reasoned that such screens may not adequately interrogate Cas9 on-target efficacy, and we instead desired a selection in which library members are continuously challenged with selective events and directly compete against other variants. To accomplish this, we incorporated elements from previous screening strategies[17] into M13 phage, which is able to package plasmids and infect *E*. *coli* in liquid culture. In this selection scheme, *E*. *coli* library members house a plasmid (pEvol) containing a low-copy inducible SpCas9 variant and the gRNA of interest. This guide is targeted to a cleavage site on the phagemid (S2 Fig). The phagemid (pSelect) further contains an antibiotic resistance cassette and an inducible toxin. Selection for antibiotic resistance, in this case chloramphenicol, creates negative selection pressure against cleavage at the target site on the pSelect phagemid. Alternatively, inducing toxin expression creates positive selection for cleavage. Positive selection can be used to select for on-target cleavage, while negative selection can be used to select against undesired off-targets.

This phage-based system allows for repetitive and pooled selective events in a single liquid culture of *E*. *coli*. Library members are challenged numerous times with phagemid after successive transductions. Through antibiotic resistance or toxin expression, Cas9 activity at the target site on the phagemid is tied directly to fitness of the *E*. *coli* growth in liquid culture. Thus, fitter variants are enriched in the population during growth. We demonstrate the function of this system by monitoring the OD_600_ of cultures in various selective contexts, shown in S3 Fig. As expected, *E*. *coli* expressing wild-type Cas9 and a non-targeting gRNA exhibit a decrease in fitness after induction of toxin expression relative to *E*. *coli* with uninduced toxin. However, expression of a properly targeted gRNA rescues growth rates and fitness phenotype. Library members with beneficial mutations to Cas9 in the context of the selection should thus receive fitness advantages and enrichment relative to other library members.

Likewise, the negative selection is highly efficient in liquid growth. Chloramphenicol resistance is disrupted when a gRNA is able to cleave the pSelect phagemid containing an off-target, resulting in a decrease in fitness and growth rate relative to unchallenged *E*. *coli* (S3 Fig). However, a library pool containing a variety of Cas9 variants is able to restore fitness through the enrichment of those which do not cut efficiently at the off-target site.

**Fig 2:**
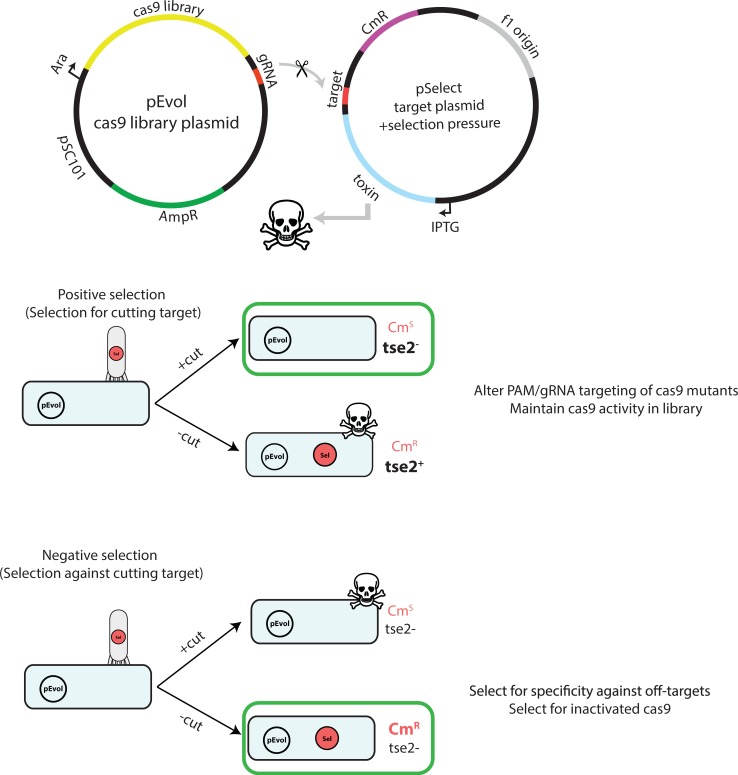
SMOOT libraries and phage selection can be used to evolve Cas9 specificity against specific off-targets.

Our phage-based selection method allows for rapid competition among many members of a library. We combine our SMOOT libraries with this high-throughput selection in order to evolve variants over multiple rounds (Fig 2). Each round of mutagenesis and selection can be accomplished in 2–3 days, allowing rapid and comprehensive directed evolution of Cas9 with approximately 10^6^ variants per round. We expect these methods to be adaptable to myriad selection experiments independent of nuclease of interest, target to be selected for, and undesired cleavage events to be selected against.

Each round of evolution starts with constructing a library of SpCas9 mutants using our SMOOT protocol, which takes one day. The library is then transformed into electrocompetent *E*. *coli* and transduced by M13KO7 Helper Phage containing an on-target DNA sequence and an inducible bacterial toxin. Successful mutants that maintain on-target activity will cleave the selection plasmid and prevent the toxin from being expressed. After the positive selection, the *E*. *coli* library is transduced with M13KO7 containing off-targets. Antibiotic can be added to the growth to select for mutants that do not cleave the off-target selection plasmid. Surviving colonies can then be pooled together and subjected to PacBio sequencing.

### Directed evolution of Cas9 mitigates off-target activity while maintaining on-target cleavage

**Fig 3:**
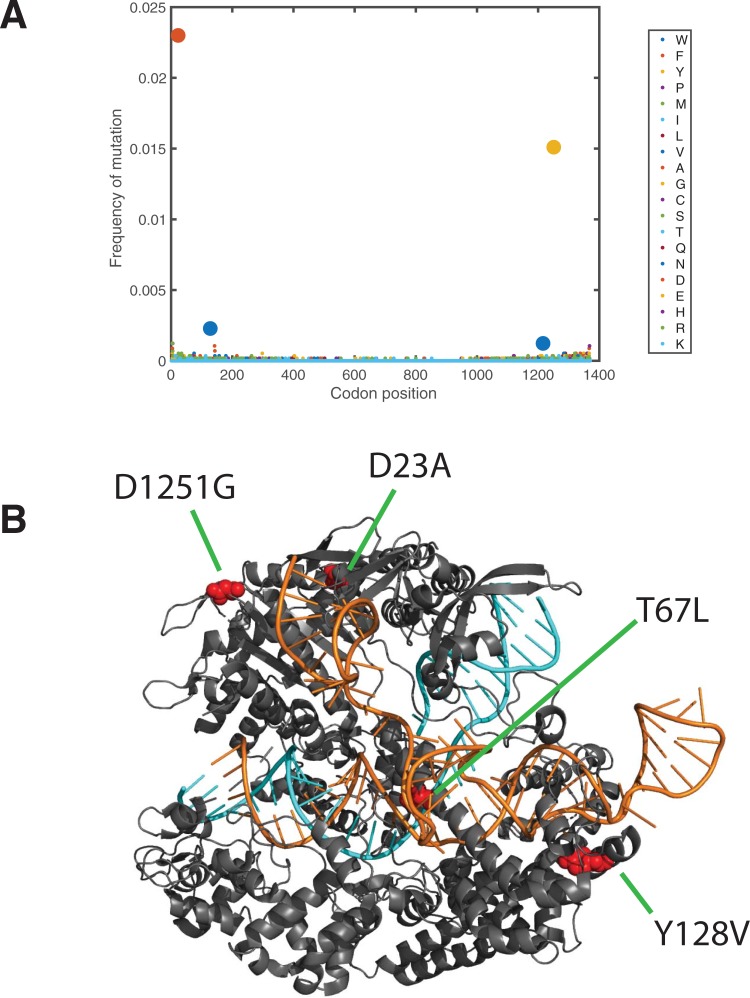
Distinct mutations were enriched after three rounds of evolution. (A) The frequency of all amino acid mutations across the Cas9 sequence. Strongly enriched mutations are marked using larger dots. (B) Locations of SpartaCas mutations drawn on the structure of SpCas9 (PDB: 5F9R). Substrate DNA is presented in orange, gRNA in cyan, and mutated residues are illustrated as red spheres (D23A, Y128V, S1216V D1251G).

**Fig 4:**
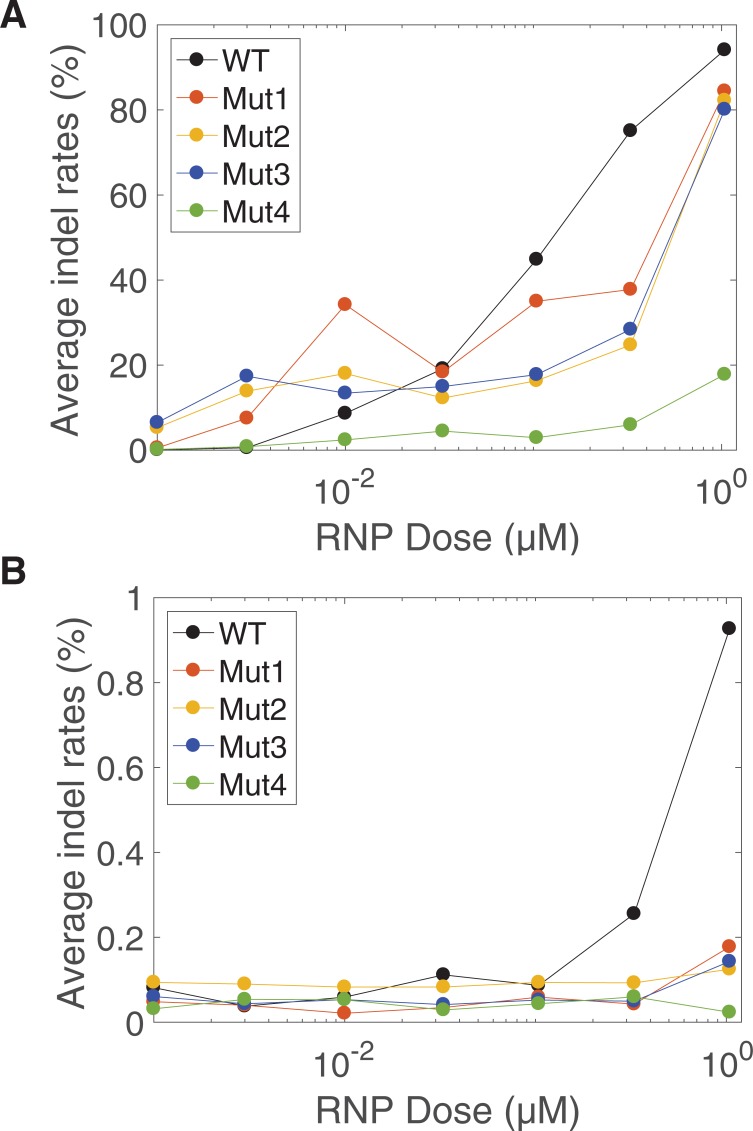
Enriched variants provide high on-target efficacy and reduced cleavage at an off-target locus in T-cells. (A) Indel fraction at the on-target locus measured in a dose response of four mutant proteins and the wildtype SpCas9. (B) Indel fraction at a single off-target locus measured in a dose response of four mutant proteins and the wildtype SpCas9. Experiments were performed in duplicate, and the value shown is the average of the two replicates.

Using our platform, we subjected SpCas9 targeted by the gRNA PD49 to three rounds of directed evolution, selecting for maintenance of on-target activity and against activity at a set of human genomic off-targets. These off-targets we previously identified as significant via GUIDE-seq after genome editing experiments using this gRNA in T-cells (S1 Table). Each round consisted of SMOOT mutagenesis of the enriched library from the previous round, selection for on-target cleavage, and selection against the three pooled off-targets. After three rounds of mutation and selection, the resulting library was sequenced using single-molecule real-time sequencing (PacBio). Long-read sequencing identified markedly enriched mutations (Fig 3). All mutations identified were distinct from previously published mutant residues (S4 Fig). Further, mutations were not concentrated in any specific domain, but rather were distributed throughout the protein. We synthesized and purified four mutant SpCas9 proteins containing combinations of these mutations (S3 Table) and evaluated their editing efficiency in T-cells at the on-target and off-target sites. All four mutant proteins decreased editing at the off-target site to background levels (Fig 4), while most mutants showed near wild-type levels of editing at the on-target. The strong reduction of off-target editing suggests that our selection strategy was highly stringent.

We chose a mutant with four of the most enriched residues (D23A, T67L, Y128V, and D1251G) to further characterize in biochemical cleavage assays and T-cell editing experiments (Fig 3 and S5 Fig). This mutant, termed *S*. *pyogenes* Adapted to Reduce Target Ambiguity Cas9 (SpartaCas), indeed demonstrated intrinsic discrimination against the off-target DNA sequences *in vitro*. SpartaCas plateaus in cleavage activity at levels 43% of that of wild-type SpCas9 when confronted only with off-target DNA duplexes, while dose responses at the on-target DNA remain indistinguishable from wild-type enzyme. The discriminatory enhancement of SpartaCas is therefore inherent to the enzyme.

**Fig 5:**
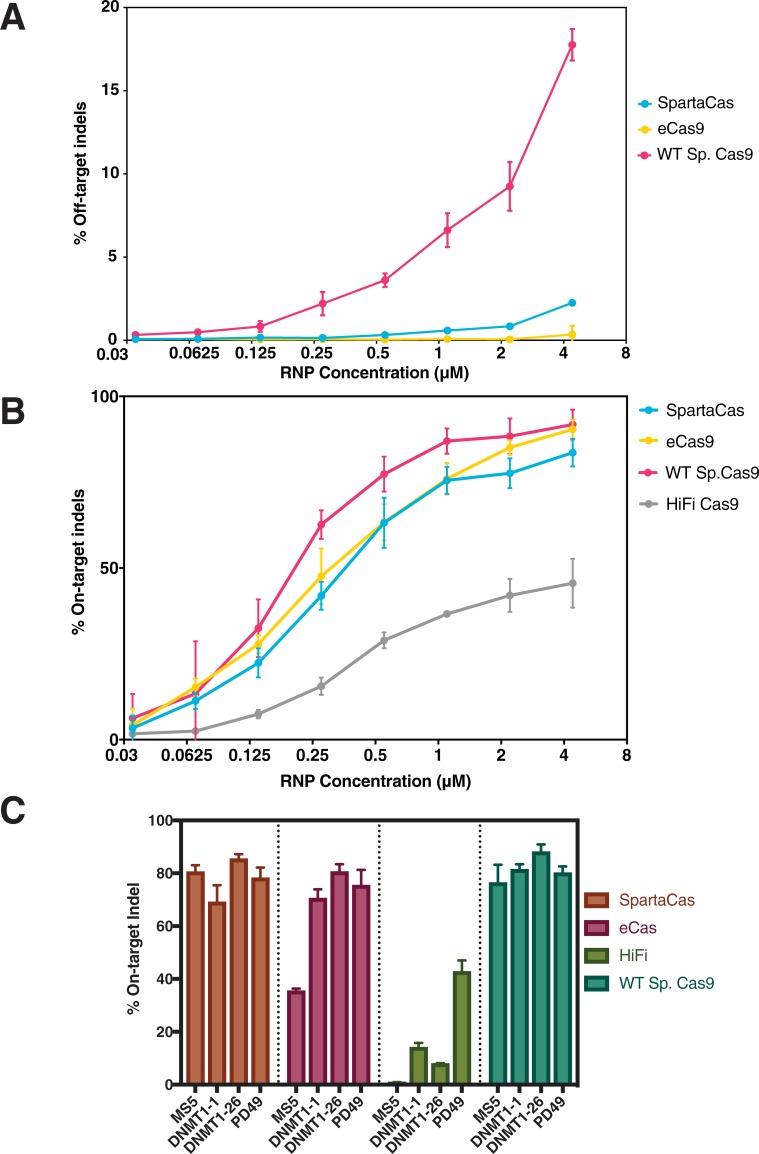
SpartaCas shows high efficacy at on-target loci and low off-target editing in T-cells. (A) Dose-response of indel fraction at the known off-target locus evolved against during phage-based selection. SpartaCas shows similar reduction in off-target editing to eCas9. (B) Dose-response of indel fraction at the on-target locus PD49. (C) On-target editing efficiency compared between wild-type and high-fidelity variants. SpartaCas maintains higher or similar editing efficiency to eCas9 and substantially higher efficiency than HF4. All experiments were performed in technical triplicates. Bars shown indicate standard error.

To assess whether SpartaCas broadly maintained on-target activity across various genomic loci, we tested it along with SpCas9-HF4(9), eSpCas9(8), and wild-type SpCas9 in T-cells Fig 5C). Although editing efficiency for SpartaCas tended to be reduced relative to wild-type, we observed dramatically higher editing efficiency using SpartaCas than HF4 at all sites tested as well as eCas9 at most loci, ranging from 97.9% activity of the eCas9 at the DNMT1-1 site to a 227% increase in activity at the MS5 site. At the PD49 site at which SpartaCas was evolved, SpartaCas demonstrated comparable activity to eCas9, editing at 78.2% and 75.4% indel efficiency respectively at peak dose. While each high-fidelity variant exhibits target-dependent effects on activity, SpartaCas performed well at all targets, including at the locus it was evolved for.

**Fig 6:**
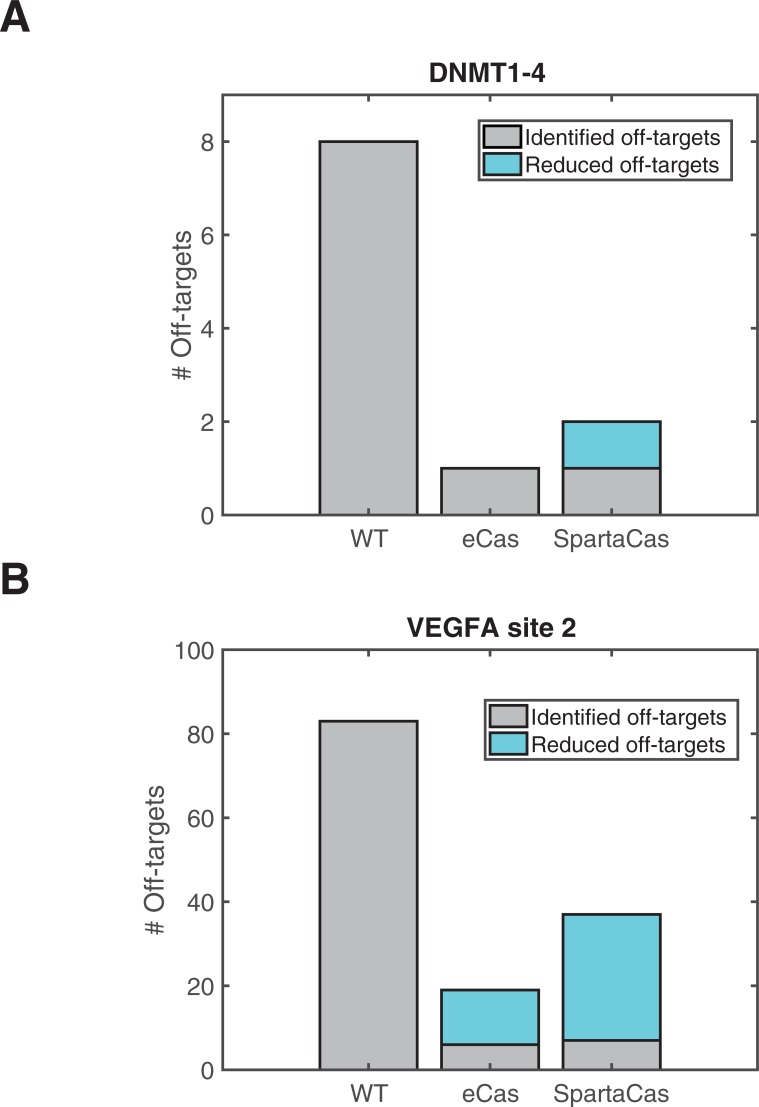
SpartaCas shows similar off-target reduction compared to eCas9 at two additional promiscuous loci. (A) Off-targets found via GUIDE-seq with the guide DNMT1-4. (B) Off-targets found via GUIDE-seq with guide VEGFA site 2. The total height of bars indicates total off-targets identified. The cyan portion of bars indicates off-targets that remain present in each data set relative to wildtype but have reduced counts in GUIDE-seq analysis. Data is shown from a single GUIDE-seq experiment for each sample.

Finally, we performed GUIDE-seq on T-cells subjected to genome editing via SpartaCas in order to determine the extent of off-target reduction at additional sites. With known promiscuous gRNAs (DNMT1-4 and VEGFA), we observed stark reduction in the number of off-target sites identified by GUIDE-seq (Fig 6 and S1 Table). Additionally, we observed a decrease in counts at identified sites relative to the wild-type enzyme. Our results show that SpartaCas performs comparably to eCas9 in reduction of off-target editing with gRNAs tested. Further, no additional off-targets were identified for the PD49 gRNA, suggesting that directed evolution of SpCas9 against specific off-targets does not trade-off specificity at other sequences (S1 Table).

Our improved directed evolution suite of SMOOT library generation and phage-based selection enabled the evolution of SpartaCas, a Cas9 variant with increased fidelity. We demonstrate a rapid, simple, and universal method for comprehensive codon mutagenesis and create the most comprehensive libraries of Cas9 to date. High-diversity libraries and competitive selection systems allowed us to engineer a Cas9 variant with improved selectivity against specific off-targets of interest. SpartaCas represents an expansion of the Cas9 toolkit to complement existing approaches to enhance specificity of genome editing. During the preparation of this manuscript, additional high-fidelity Cas9 variants have been developed[18–21]. However, none of these mutations overlap with the variant found in this study. The structure-activity relationship of these four mutations contained in this variant could provide new insight into the complex biophysics of Cas9 cleavage and target discrimination. The successful application of our system demonstrates the ability to mitigate activity at particular identified off-targets through directed evolution, potentially strengthening the safety of an RNA-guided endonuclease-based therapeutic. We anticipate that our platform should be generalizable to a wide array of selection conditions in which target sites can quickly be positively or negatively selected for. Further, it should be applicable to any RNA-guided endonuclease. Directed evolution of RNA-guided nucleases both broadens our understanding of their function and enables safer use as a potential therapeutic.

## Methods

### SMOOT library generation

SpCas9 libraries were constructed using scanning mutagenesis of oligo-directed targets (SMOOT), a modified PCR with 5’-phosphorylated oligonucleotides (Integrated DNA Technologies) containing a single degenerate codon in the middle of their sequence targeting every codon position along the entire length of the protein (S5 Table). Primers were ordered resuspended from the vendor in equimolar pool at 100μM 1xIDTE. In each round of the PCR cycle, the oligonucleotides bind all along the Cas plasmid along with a reverse primer that bound in the T7 terminator and an optional forward primer (S4 Table). Phusion^®^ Hot Start Flex 2X Master Mix (New England Biolabs) DNA polymerase was used due to its very low strand displacing activity, resulting in multiple mutations per mutant. A forward primer binding in the T7 terminator downstream of the gRNA was spiked in, allowing for fine-tuning of the mutation rate of the library with the PCR conditions found in S4 Table and S5 Table. The library was then digested with 20 units Exonuclease I (New England Biolabs), 5 units Lambda Exonuclease (New England Biolabs), and 20 units DpnI (New England Biolabs) to remove the starting template and reaction byproducts that were not full length. After purifying the mutagenized plasmid library with a DNA Clean & Concentrator^™^-5 kit (Zymo Research), the mutants were electroporated into NEB^®^ Turbo Electrocompetent *E*. *coli*. 15 mL of LB with 10% glycerol was used to sweep libraries off of LB Agar Bioassay Trays with 100 μg/mL carbenicillin (Teknova) and stored in a -80°C freezer until the time of the selection.

### Packaging selection plasmid into phage

A selection plasmid containing an inducible bacterial toxin and target site was transformed into NEB^®^ Turbo Competent *E*. *coli* (High Efficiency) (S7 Fig). We started an 8 mL overnight culture of the cells shaking at 250 rpm at 37°C for 16 hours. In the morning, 200 μL of cells were then inoculated into 50 mL of Terrific Broth (Teknova) with 2.5 μL of 1X10^11^ pfu/ml M13KO7 Helper Phage (New England Biolabs). After four hours of growing the culture at 37°C at 250 rpm, cells were spun at 4500 g for 10 min in an Avanti JXN-26 centrifuge (Beckman Coulter). The supernatant was collected without disturbing the cell pellet and the centrifugation was repeated. The top 40 mL of supernatant were added to a fresh tube and we mixed with 10 mL of 2.5 M NaCl/20% PEG-8000 (w/v) (VWR). Phages were precipitated overnight at 4°C for 16 hours and then spun at 12,000 g for one hour at 4°C. Pellet was resuspended in 1 mL of TBS and 200 μL of 2.5 M NaCl/20% PEG-8000 (w/v) (VWR) was added. Phages were precipitated for 3 hours on ice and then spun them at 12,000 rpm in a Sorvall^™^ Legend^™^ Micro 21R centrifuge (Thermo Scientific) for 30 minutes at 4°C. After removing the supernatant, 200 μL of 1X TBS and 200 μL of 50% glycerol were used to resuspend the pellet. The phages were stored at -20°C until the start of the selection.

### Phage selection

For positive selections to maintain on target activity, the Cas9 library was diluted 1:100 (10 μL in 1 mL of LB) and 1 μL of the dilution was added along with 10 μL of phage containing the on-target in 1 mL of LB. Phages were allowed to transduce the E. coli population shaking at 250 rpm for 1 hour at 37C before 1 mM isopropyl β-D-1-thiogalactopyranoside (IPTG) was added to the culture to induce expression of the toxin. Liquid cultures were shaken overnight at 250 rpm at 37°C to enrich for successful variants for 16 hours. Selected libraries were stored as glycerol stocks at -80°C in a final glycerol concentration of 25% v/v until the next selection.

For the negative selection, 1 μL of the positive selection cell stocks was added to 1 mL of LB along with 100 μg/mL of ampicillin and 3.33 μL of each phage containing one of the three PD1 off-targets After transduction for 1 hour at 250 rpm at 37°C, 1μL of 50 mg/mL of chloramphenicol was added to the liquid cultures to select for mutants that were unable to cleave the off-target. After overnight growth at 37°C and 250 rpm, cultures were miniprepped using the QIAprep Spin Miniprep Kit (Qiagen) to isolate enriched Cas9 mutants to serve as the template in the next round of mutagenesis. The libraries each underwent three rounds of SMOOT mutagenesis, positive selection, and negative selection.

### Next-generation sequencing

Libraries that passed positive and negative selections were prepared for long-read PacBio sequencing using PCR. Phusion^®^ Hot Start Flex 2X Master Mix (New England Biolabs) DNA polymerase was used according to the manufacturer’s protocol. The PCR is described in detail in the supplement (S4 Table and S6 Table). Libraries were purified with DNA Clean & Concentrator^™^-5 (Zymo Research, #D4013). PacBio SMRT sequencing was performed by the Icahn School of Medicine at Mount Sinai (New York, NY).

### Protein purification

Protein expression plasmid vectors containing the eight Cas9 variants of interest from the PD1 selections were synthesized by Genewiz with human codon optimization (S8 Fig). Expression constructs were transformed into Novagen Rosetta^™^ 2(DE3) Singles^™^ Competent Cells according to the manufacturer’s protocol, and colonies were grown in 8mL of LB w/ 50 μL/mL of kanamycin and 15 μg/mL chloramphenicol at 250 rpm at 37°C for 16 hours. Each culture was inoculated into 1 L of Terrific Broth (Teknova) and shaken at 37°C at 250 rpm until the OD reached 2.0. 1 mM IPTG was then added and cells were allowed to express the proteins overnight shaking at 250 rpm at 18°C for 16 hours. After centrifuging the cells at 12,230 g in the Avanti JXN-26 centrifuge (Beckman Coulter), the pellet was resuspended with TG1000 (50 mM Tris, 10% glycerol and 1000 mM NaCl, 1 mM Bond-Breaker^™^ TCEP Solution Neutral pH [Thermo Scientific], 1:1000 Protease Inhibitor Cocktail, Animal Component Free [Sigma]) and the suspension was put through the Microfluidizer^™^ LM10 for lysis. The cytosolic fraction was collected by spinning at 31,000 g in the Avanti JXN-26 centrifuge (Beckman Coulter) for 30 minutes. The supernatant was incubated with 2 mL HisPur^™^ Cobalt Resin (Thermo Scientific) and 10 mM Imidazole pH 8 (Boston Bioproducts) up to a volume of 50 mL while rocking for 1 hour at 4°C. Samples were washed with TG300 buffer (50 mM Tris, 300 mM NaCl, 20% glycerol) and eluted with 300 mM imidazole on a gravity column. Proteins were further purified by the HiTrap SP HP 5mL cation exchange chromatography column (GE Healthcare Life Sciences) and the Sepax SRT-10 SEC-300 size exclusion chromatography column (Sepax Technologies) on the Äkta pure 25 FPLC (GE Healthcare Life Sciences).

### Complexation of variant Cas9 RNPs

Purified SpCas9 variants were complexed with two-part gRNA (IDT). Prior to complexation, all gRNAs were annealed at a concentration of 200 μM, with a 1:1 molar ratio of crRNA to tracrRNA. Cas9:gRNA holoenzymes were complexed at a 1:2 molar ratio of apoprotein to RNA. Solutions of 100 μM protein and 200 μM gRNA were mixed at a 1:1 ratio by volume to achieve a final RNP concentration of 50 μM, and the complexation reaction occurred for 30 minutes at room temperature. The RNPs were then serially diluted 2-fold across eight concentrations. RNPs were flash frozen in liquid nitrogen and stored at -80° C until biochemical assays and T-cell nucleofection.

### Biochemical cleavage testing

Target cleavage efficiency was measured *in vitro* by a dose-response of Cas9 variant RNP against 2 nM synthetic DNA substrate for 1 hour at 37°C. RNP was added and mixed with DNA substrate using a Biomek FX^p^ liquid handler. Cas9 activity was quenched by addition of Proteinase K (New England Biolabs, #P8107S), and RNA was digested using RNase If (New England Biolabs, #M0243). Fraction of substrate cleaved was measured using the Fragment Analyzer automated capillary electrophoresis system (AATI). Dose response analysis was performed with GraphPad Prism using four parameter logistic regression.

### Culture of T-Cells

T-Cells were cultured with Lonza X-Vivo 15 media. The cells were thawed and cultured with Dynabeads Human T-Activator CD3/CD28 for T Cell Expansion and Activation. On day two post thaw that cells were removed from the beads. The cells continued to be cultured to day four, upon which they were spun down for nucleofection. On day two post nucleofection the cell volume was divided in half into a new plate so that they had continued room to expand.

### T-Cell nucleofection

Cells were counted using the BioRad T-20 cell counter. Cells were mixed 1:1 with trypan blue and counted. The total amount of cells needed (enough for 500k cells per well) were aliquoted to a separate tube and then spun down at 1500 rpm for 5 minutes. The cells were then resuspended in Lonza P2 nucleofection solution. The cells were then plated at 20 μL per well in the Lonza 96 well nucleofection plate. Cells and RNP plates were then brought over to the BioMek FX robot. Using the 96-well head 2 μL of each RNP was transferred and mixed into the nucleofection plate. The nucleofection plate was then immediately brought over to the Lonza shuttle system where it was nucleofected with the DS-130 pulse code. Cells were then immediately brought back to the BioMek FX where they were transferred to a pre-warmed 96-well nontreated media plate and mixed. The cell plate was then placed at 37° C for incubation.

### gDNA extraction

On day four cells were spun down in their plates at 2000 rpm for 5 minutes. The media was then decanted. The cell pellets were then resuspended in Agencourt DNAdvance lysis solution. The gDNA was extracted using the DNAdvance protocol on the BioMek FX.

### GUIDE-seq

GUIDE-seq was performed based on the protocol of Tsai et al. 2015[22]and adapted to T-cells as follows. 10 μL of 4.4 μM RNP were combined with 4 μL of 100 μM dsODN, and 6 μL of 1xH150 buffer for a total volume of the 20 μL. RNPs were placed on ice until nucleofection. T-cells were counted using the BioRad T-20 cell counter. Cells were mixed 1:1 with trypan blue and counted. The total amount of cells needed (enough for 2 million cells per cuvette) were aliquoted to a separate tube and then spun down at 1500 rpm for 5 minutes. The cells were then resuspended in 80 μL of Lonza P2 solution. Cells were then pipetted into their respective cuvettes, and the 20 μL of RNP/dsODN were added to each cuvette, and the whole solution was gently mixed. Cells were then nucleofected using the CA-137 pulse code. Cells were then immediately pipetted into a pre-warmed noncoated media plate. The cell plate was then placed at 37° C for incubation. gDNA was extracted and analyzed using the protocol of Tsai et al. 2014, using only bidirectional reads.

## Supporting information

S1 Fig SMOOT is effective to create comprehensive codon mutagenesis throughout a protein. Original SMOOT protocols were optimized for SaCas9 before being applied to SpCas9. (A) Diagram of first round SMOOT products. Oligonucleotides with degenerate triplet bases in the middle of their sequence targeting every codon position along the length of the protein will bind to the parent plasmid in the first round of SMOOT. Bound oligonucleotides will extend until they reach an abutting DNA fragment. The reverse primer will bind outside of the mutagenized Cas9 region and will terminate when it reaches a nick. However, in subsequent rounds, each of these fragments will be able to anneal either with each other or the parent plasmid and extend to become a full length nicked circular plasmid. A ten-minute extension time helps with some of these inefficiencies, but undesirable products remain in the reaction. At the end of the reaction, linear products will be digested by the Lambda Exonuclease, Exo I, and Exo III cleanup step. (B) SMOOT is able to access all amino acid mutations throughout the protein. Heat map of amino acid mutations found as a function of codon position. (C) SMOOT makes mutations throughout the protein. Counts of mutations found as a function of codon position.
(EPS)

S2 Fig Phage can be used to select for Cas9 activity in *E*. *coli*. (A) In the two-plasmid system, each *E*. *coli* cell will contain both a pEvol plasmid with a Cas9 mutant and gRNA and a pSel plasmid with a bacterial toxin and target site. (B) Diagram of selection conditions for or against Cas9 cleavage activity. In the positive selections, active mutants survive by cleaving the matching on-target on the selection plasmid to reduce toxin expression. In the negative selections, antibiotic is added to select for mutants that cannot cleave a mismatched off-target on the selection plasmid.
(EPS)

S3 Fig Phage-based selection is effective in liquid culture. (A) *E*. *coli* harboring Cas9 correctly targeted to a selection phagemid (red) experience limited fitness decrease in selective conditions relative to *E*. *Coli* either without induced Cas9 (blue) or with nonmatching Cas9 (green). Correctly targeted Cas9 can thus be selected for to cleave phagemid DNA. (B) *E*. *coli* expressing correctly targeted Cas9 experience significant fitness decrease in negative selection for chloramphenicol maintenance (blue) relative to the library of Cas9 mutants which are able to escape negative selection (red). Growth curves represent data from a single experiment.
(EPS)

S4 Fig SpartaCas mutations are distinct from those found in previously engineered high-fidelity Sp. Cas9 proteins. Mutations introduced relative to the wildtype Cas9 are indicated in red sphere on the protein structure (pdb:5f9r). Template DNA is shown in cyan, and the gRNA is shown in orange.
(EPS)

S5 Fig SpartaCas maintains specificity in a biochemical assay, indicating intrinsic specificity advantages of the enzyme. Cleavage of a synthetic DNA substrate was measured as a function of increasing Cas9 RNP dose. Cleavage of the off-target (dots) relative to the on-target (triangles) was lower for SpartaCas (orange) as compared to wildtype Sp. Cas9 (black). The experiment was performed in technical triplicate. Bars indicate standard error.
(EPS)

S6 Fig Plasmid map of the SpCas9 library. (DOCX)

S7 Fig Plasmid map of the selection plasmid containing the bacterial toxin and the target site. (DOCX)

S8 Fig The expression construct used to generate the SpartaCas protein. (DOCX)

S9 Fig Sp.Cas9 library diversity after first round of evolution. Sp.Cas9 shows diverse library representation after selection from round 1 (A,B). After enriching for selective residues over one round, most amino acid mutations are still present in the library.
(EPS)

S1 Table GUIDE-seq results for wildtype Sp.Cas9, eCas, and SpartaCas. (DOCX)

S2 Table The PD1 on target and three off target sequences contained within each selection plasmid used during the initial directed evolution experiments. (DOCX)

S3 Table Mutants evaluated in this study. (DOCX)

S4 Table Primers used for SMOOT reactions and PacBio library preparation. (DOCX)

S5 Table SMOOT reaction conditions. (DOCX)

S6 Table Reaction conditions for the PCR to prepare for PacBio sequencing. (DOCX)

## References

[1] Doudna JA, Charpentier E. The new frontier of genome engineering with CRISPR-Cas9. Science. 2014 11 28;346(6213):1258096–1258096. 10.1126/science.1258096 25430774

[2] Hsu PD, Lander ES, Zhang F. Development and Applications of CRISPR-Cas9 for Genome Engineering. Cell. 2014 6;157(6):1262–78. 10.1016/j.cell.2014.05.010 24906146 PMC4343198

[3] Pattanayak V, Lin S, Guilinger JP, Ma E, Doudna JA, Liu DR. High-throughput profiling of off-target DNA cleavage reveals RNA-programmed Cas9 nuclease specificity. Nat Biotechnol. 2013 8 11;31(9):839–7. 10.1038/nbt.2673 23934178 PMC3782611

[4] Chen S, Sanjana NE, Zheng K, Shalem O, Lee K, Shi X, et al Genome-wide CRISPR Screen in a Mouse Model of Tumor Growth and Metastasis. Cell. 2015 3 12;160(6):1246–60. 10.1016/j.cell.2015.02.038 25748654 PMC4380877

[5] Doench JG, Fusi N, Sullender M, Hegde M, Vaimberg EW, Donovan KF, et al Optimized sgRNA design to maximize activity and minimize off-target effects of CRISPR-Cas9. Nat Biotechnol. 2016 1 18;34(2):184–91. 10.1038/nbt.3437 26780180 PMC4744125

[6] Lin J, Wong K-C. Off-target predictions in CRISPR-Cas9 gene editing using deep learning. Bioinformatics. 2018 9 8;34(17):i656–63. 10.1093/bioinformatics/bty554 30423072 PMC6129261

[7] Kim D, Bae S, Park J, Kim E, Kim S, Yu HR, et al Digenome-seq: genome-wide profiling of CRISPR-Cas9 off-target effects in human cells. Nature. 2015; 10.1038/nmeth.3284 25664545

[8] Slaymaker IM, Gao L, Zetsche B, Scott DA, Yan WX, Zhang F. Rationally engineered Cas9 nucleases with improved specificity. Science. 2015 12 1;351(6268):84–8. 10.1126/science.aad5227 26628643 PMC4714946

[9] Kleinstiver BP, Pattanayak V, Prew MS, Tsai SQ, Nguyen NT, Zheng Z, et al High-fidelity CRISPR–Cas9 nucleases with no detectable genome-wide off-target effects. Nature. 2016 1 6;529(7587):490–5. 10.1038/nature16526 26735016 PMC4851738

[10] Stella S, Alcón P, Montoya G. Class 2 CRISPR–Cas RNA-guided endonucleases: Swiss Army knives of genome editing. Nat Struct Amp Mol Biol. 2017 11 1;24(11):882–92. 10.1038/nsmb.3486 29035385

[11] Long C, Li H, Tiburcy M, Rodriguez-Caycedo C, Kyrychenko V, Zhou H, et al Correction of diverse muscular dystrophy mutations in human engineered heart muscle by single-site genome editing. Sci Adv. 2018 1 1;4(1):eaap9004 10.1126/sciadv.aap9004 29404407 PMC5796795

[12] Pritchard L, Corne D, Kell D, Rowland J, Winson M. A general model of error-prone PCR. J Theor Biol. 2005 6 21;234(4):497–509. 10.1016/j.jtbi.2004.12.005 15808871

[13] Araya CL, Fowler DM. Deep mutational scanning: assessing protein function on a massive scale. Trends Biotechnol. 2011 5 9;435–42. 10.1016/j.tibtech.2011.04.003 21561674 PMC3159719

[14] Siloto RMP, Weselake RJ. Site saturation mutagenesis: Methods and applications in protein engineering. Biocatal Agric Biotechnol. 2012 7;1(3):181–9.

[15] Wrenbeck EE, Klesmith JR, Stapleton JA, Adeniran A, Tyo KEJ, Whitehead TA. Plasmid-based one-pot saturation mutagenesis. Nat Methods. 2016 11 1;13(11):928–30. 10.1038/nmeth.4029 27723752 PMC5666567

[16] Kitzman JO, Starita LM, Lo RS, Fields S, Shendure J. Massively parallel single-amino-acid mutagenesis. Nat Publ Group. 2015 3;12(3):203–6-4 p following 206. 10.1038/nmeth.3223 PMC4344410 25559584

[17] Kleinstiver BP, Prew MS, Tsai SQ, Topkar VV, Nguyen NT, Zheng Z, et al Engineered CRISPR-Cas9 nucleases with altered PAM specificities. Nature. 2015 7 23;523(7561):481–5. 10.1038/nature14592 26098369 PMC4540238

[18] Casini A, Olivieri M, Petris G, Montagna C, Reginato G, Maule G, et al A highly specific SpCas9 variant is identified by &lt;i&gt;in vivo&lt;/i&gt; screening in yeast. Nat Biotechnol. 2018 1 29;533:103. 10.1038/nbt.4066 PMC6066108 29431739

[19] Ikeda A, Fujii W, Sugiura K, Naito K. High-fidelity endonuclease variant HypaCas9 facilitates accurate allele-specific gene modification in mouse zygotes. Commun Biol. 2019 12;2(1):371. 31633062 10.1038/s42003-019-0627-8 PMC6787007

[20] Lee JK, Jeong E, Lee J, Jung M, Shin E, Kim Y, et al Directed evolution of CRISPR-Cas9 to increase its specificity. Nat Commun. 2018 8 6;9(1):3048 10.1038/s41467-018-05477-x 30082838 PMC6078992

[21] Vakulskas CA, Dever DP, Rettig GR, Turk R, Jacobi AM, Collingwood MA, et al A high-fidelity Cas9 mutant delivered as a ribonucleoprotein complex enables efficient gene editing in human hematopoietic stem and progenitor cells. Nat Med. 2018 8 1;24(8):1216–24. 10.1038/s41591-018-0137-0 30082871 PMC6107069

[22] Tsai SQ, Zheng Z, Nguyen NT, Liebers M, Topkar VV, Thapar V, et al GUIDE-seq enables genome-wide profiling of off-target cleavage by CRISPR-Cas nucleases. Nat Biotechnol. 2015 2;33(2):187–97. 10.1038/nbt.3117 25513782 PMC4320685

