# Supplementary material for: SMOOT libraries and phage-induced directed evolution of Cas9 to engineer reduced off-target activity
Source: PLoS One. 2020 Apr 16;15(4):e0231716. doi: 10.1371/journal.pone.0231716 (PMC7161989; doi:10.1371/journal.pone.0231716)
Supplement: S6 Fig — (DOCX) [file pone.0231716.s006.docx]

aaatctagagcggttcagtagaaaagatcaaaggatcttcttgagatcctttttttctgcgcgtaatcttttgccctgtaaacgaaaaaaccacctggggaggtggtttgatcgaaggttaagtcagttggggaactgcttaaccgtggtaactggctttcgcagagcacagcaaccaaatctgtccttccagtgtagccggactttggcgcacacttcaagagcaaccgcgtgtttagctaaacaaatcctctgcgaactcccagttaccaatggctgctgccagtggcgttttaccgtgcttttccgggttggactcaagtgaacagttaccggataaggcgcagcagtcgggctgaacggggagttcttgcttacagcccagcttggagcgaacgacctacaccgagccgagataccagtgtgtgagctatgagaaagcgccacacttcccgtaagggagaaaggcggaacaggtatccggtaaacggcagggtcggaacaggagagcgcaagagggagcgacccgccggaaacggtggggatctttaagtcctgtcgggtttcgcccgtactgtcagattcatggttgagcctcacggctcccacagatgcaccggaaaagcgtctgtttatgtgaactctggcaggagggcggagcctatggaaaaacgccaccggcgcggccctgctgttttgcctcacatgttagtcccctgcttatccacggaatctgtgggtaactttgtatgtgtccgcagcgcccgccgcagtctcacgcccggagcgtagcgaccgagtgagctagctatttgtttatttttctaaatacattcaaatatgtatccgctcatgagacaataaccctgataaatgcttcaataatattgaaaaaggaagagtatgagtattcaacatttccgtgtcgcccttattcccttttttgcggcattttgccttcctgtttttgctcacccagaaacgctggtgaaagtaaaagatgctgaagatcagttgggtgcacgagtgggttacatcgaactggatctcaacagcggtaagatccttgagagttttcgccccgaagaacgttttccaatgatgagcacttttaaagttctgctatgtggcgcggtattatcccgtattgacgccgggcaagagcaactcggtcgccgcatacactattctcagaatgacttggttgagtactcaccagtcacagaaaagcatcttacggatggcatgacagtaagagaattatgcagtgctgccataaccatgagtgataacactgcggccaacttacttctgacaacgatcggaggaccgaaggagctaaccgcttttttgcacaacatgggggatcatgtaactcgccttgatcgttgggaaccggagctgaatgaagccataccaaacgacgagcgtgacaccacgatgcctgtagcaatggcaacaacgttgcgcaaactattaactggcgaactacttactctagcttcccggcaacaattaatagactggatggaggcggataaagttgcaggaccacttctgcgctcggcgcttccggctggctggtttattgctgataaatctggagccggtgagcgtgggtctcgcggtatcattgcagcactggggccagatggtaagccctcccgtatcgtagttatctacacgacggggagtcaggcaactatggatgaacgaaatagacagatcgctgagataggtgcctcactgattaagcattggtaataatgtctaacaattcgttcaagccgaggggccgcaagatccggccacgatgacccggtcgtcggttcagggcagggtaccaggcacgcctaaccgtcagtgagattggatgagtgaacgatattgatcgagaagagccctgcgcagccgctgccgtgcctgcaggaagcaacggcccggagggtggcgggcaggacgcccgccataaactgccaggcatcaaattaagcagaaggccatcctgacggatggcctttttgcgtttctacaaactctgctagcAAGCTGTTGTGACCGCTTGCTCTAGCCAGCTATCGAGTTGTGAACCGATCCATCTAGCAATTGGTCTCGATCTAGCGATAGGCTTCGATCTAGCTATGTAGAAACGCCGTGTGCTCGATCGCTTTATAAGGTCCACAGTAGCTGCTATAATTGCTTCAACAGAACATATTGACTATCCGGTATTACCCGGCAGATCTTTGTCGATCCTACCATCCACTCGACACACCCGCCAGCGGCCGCTGCCAAGCTTCCGAGCTCTCGAATTCAAAGGAGGTACCCACCatggacaaaaagtatagcattggtctggacattggcaccaatagcgttggctgggcagtgatcaccgacgaatataaagtgcctagcaagaagttcaaagttctgggcaatacagaccgccacagcatcaaaaagaacctgatcggcgcactgctgttcgacagcggcgaaaccgccgaagccacacgcttaaaacgcacagcacgtcgccgctacacccgccgcaaaaaccgcatctgctatctgcaggagatcttcagcaacgagatggccaaggttgacgatagctttttccatcgcctggaggagagcttcctggttgaggaagataagaagcacgaacgccacccgattttcggcaacatcgtggatgaggttgcctatcatgagaagtaccctacaatttatcacttacgcaagaaactggttgacagcaccgacaaggccgacttacgtttaatttatctggccctggcccatatgattaagtttcgtggtcattttctgatcgagggcgatctgaacccggataacagcgacgtggataaattatttattcagttagtgcagacatacaatcagctgtttgaggaaaaccctatcaacgccagcggcgttgatgccaaggccatcttaagtgcccgcctgagtaagagccgtcgtctggagaatctgattgcccagctgccgggtgagaagaagaatggcctgtttggcaacctgatcgccttaagcctgggcctgacaccgaattttaagagcaactttgacctggcagaagacgccaagctgcagttaagcaaagatacatacgatgatgatttagataacttactggcacagatcggtgaccaatatgccgatctgtttttagccgcaaaaaatctgagtgacgccattttactgagcgatattctgcgcgttaacaccgagatcacaaaagcccctctgagcgccagcatgattaagcgctatgacgagcaccaccaggacctgacattactgaaggccctggtgcgtcaacagctgccggagaaatataaagaaattttcttcgatcaaagcaaaaacggttacgccggctatatcgacggtggtgccagtcaagaagaattctacaaatttattaaacctattctggagaagatggatggcaccgaggaattactggtgaaactgaaccgtgaggacctgctgcgtaaacagcgcacctttgataacggcagcatcccgcaccagatccacttaggcgagttacatgccattctgcgccgccaagaagatttttatcctttcttaaaggacaaccgtgaaaaaattgagaaaattctgacattccgcatcccgtattacgttggtccgttagcccgtggcaatagccgctttgcctggatgacccgcaagagtgaggagaccatcaccccgtggaatttcgaagaggtggtggataagggcgccagtgcccagagcttcattgaacgtatgaccaatttcgataaaaatttaccgaacgaaaaggtgctgccgaagcacagcctgctgtacgagtattttacagtgtataacgagctgacaaaagttaaatacgtgaccgaaggtatgcgcaaaccggcatttctgagtggcgagcaaaagaaagccatcgtggacctgctgttcaagaccaaccgcaaggtgaccgtgaaacagctgaaagaagactattttaagaaaattgaatgctttgatagtgttgaaatcagcggcgttgaagaccgctttaatgccagcctgggcacctatcatgatctgttaaagattatcaaagataaggatttcctggataatgaggagaatgaagacatcctggaggacattgtgctgacactgaccctgttcgaagatcgcgaaatgattgaggagcgcctgaagacctatgcacatctgtttgacgacaaagtgatgaagcagctgaagcgtcgtcgttatacaggttggggccgtctgagccgtaaactgatcaatggcatccgcgacaagcagagtggtaaaaccatcctggacttcttaaaatctgacggtttcgcaaaccgcaacttcatgcagctgatccacgacgacagtctgacattcaaggaagacattcagaaagcccaggttagcggccagggcgatagcctgcatgagcatatcgccaacctggcaggtagcccggccattaagaagggcatcctgcagaccgtgaaagtggtggatgaactggtgaaggttatgggccgccacaagcctgaaaacattgtgatcgagatggcccgcgaaaaccagacaacccaaaaaggtcagaaaaacagccgcgaacgtatgaagcgtattgaagagggtatcaaggaactgggcagccaaatcctgaaggaacacccggtggagaacacccagctgcaaaacgaaaagttatacctgtactatctgcagaatggccgtgacatgtacgtggaccaagagctggacatcaaccgtctgagcgattacgacgttgatcacatcgttccgcaaagctttctgaaggatgatagcatcgacaacaaggtgctgacccgtagcgacaagaatcgtggcaaaagtgacaacgttccgagcgaagaagttgtgaaaaagatgaaaaattattggcgccagctgttaaacgccaaattaattacacagcgtaaatttgataatctgacaaaagccgaacgtggtggcctgagtgagttagataaagcaggttttatcaaacgccagctggtggaaacccgccaaattaccaaacacgttgcccaaattctggatagccgcatgaacacaaaatatgacgagaacgacaagctgatccgtgaggtgaaagttattaccctgaaaagtaaactggttagcgactttcgtaaagatttccagttttacaaagttcgcgagatcaacaattatcatcacgcacatgatgcctatctgaacgcagtggtgggcaccgccctgatcaagaaatatccgaagctggaaagcgaatttgtgtacggcgattacaaggtttatgacgtgcgtaaaatgatcgccaaaagtgaacaggaaatcggtaaggccacagccaagtacttcttttatagcaacatcatgaacttttttaaaacagaaattaccctggccaatggtgagatccgtaagcgcccgttaatcgaaaccaatggcgagaccggcgaaatcgtgtgggataaaggccgtgattttgccaccgtgcgtaaggttttaagcatgccgcaagtgaatattgtgaagaaaacagaggttcagaccggtggctttagtaaggaaagcattctgccgaaacgcaatagcgacaaactgattgcccgcaagaaagactgggaccctaagaaatacggcggctttgatagccctacagttgcctacagcgtgctggtggtggcaaaggttgaaaagggcaaaagtaagaaactgaaaagcgttaaagagctgttaggcatcaccatcatggagcgcagcagttttgagaagaacccgattgatttcctggaggccaaaggctataaggaagtgaagaaagacctgatcatcaaattacctaagtatagcctgtttgagctggagaatggtcgtaagcgcatgctggcaagtgcaggtgagctgcagaaaggtaacgaactggcattacctagtaagtacgtgaactttctgtatttagcaagtcactacgaaaaactgaagggcagcccggaggacaacgaacagaaacagctgtttgttgaacaacataagcattatttagatgaaattattgaacaaattagcgaatttagcaagcgcgtgattctggccgatgccaacctggataaggtgctgagcgcatacaataaacatcgcgataaaccgattcgcgagcaggcagaaaatatcatccacctgttcaccctgacaaatctgggcgcccctgccgcctttaaatactttgacaccacaattgatcgtaaacgttatacaagcaccaaggaagttctggacgcaaccctgatccatcagagcattacaggtctgtatgaaacacgtattgatctgagccaattaggtggtgattaatgaATGCATccgggacaatgaaaacgttagtcatggcgcgccttgacggctagctcagtcctaggtacagtgctagcttaatGTCTGGGCGGTGCTACAACTGTTTTAGAGCTAGAAATAGCAAGTTAAAATAAGGCTAGTCCGTTATCAACTTGAAAAAGTGGCACCGAGTCGGTGCTTTTTccgctgagcaataactagcataaccccttggggcctctaaacgggtcttgaggggttttttgacaaagaaagccgggcaatgcccggctttttCTCGAGccacgcctcctgggctgacttcaggtgctacatttgaagagataaattgcactg

1949 2036 terminator

label rrnBT2\term

2332 6438 misc_feature

label from ws013

904 1764 CDS

label AmpR

2329 6435 CDS

label bact opt SP cas9

26 764 rep_origin

label cloD13\(CDF)

2047 2237 promoter

label OBX18

2315 2328 RBS

label KOZAK_ShineDalgarno

6681 6711 terminator

label terminator

6449 6517 promoter

label J23100\promoter

6637 6619 primer

label T7\Terminator\Primer\#69337-3

6633 6680 terminator

label T7 terminator

6439 6444 misc_feature

label NsiI

6712 6717 misc_feature

label XhoI

6619 6637 misc_feature

label T7-term-R primer

6518 6537 misc_feature

label PD1 spacer

6538 6618 misc_feature

label SP tracer

**S6 Fig. Plasmid map of the SpCas9 library.**
