# Supplementary material for: SMOOT libraries and phage-induced directed evolution of Cas9 to engineer reduced off-target activity
Source: PLoS One. 2020 Apr 16;15(4):e0231716. doi: 10.1371/journal.pone.0231716 (PMC7161989; doi:10.1371/journal.pone.0231716)
Supplement: S7 Fig — (DOCX) [file pone.0231716.s007.docx]

ttgttccagtttggaacaagagtccactattaaagaacgtggactccaacgtcaaagggcgaaaaaccgtctatcagggcgatggcccactacgtgaaccatcaccctaatcaagttttttggggtcgaggtgccgtaaagcactaaatcggaaccctaaagggagcccccgatttagagcttgacggggaaagccggcgaacgtggcgagaaaggaagggaagaaagcgaaaggagcgggcgctagggcgctggcaagtgtagcggtcacgctgcgcgtaaccaccacacccgccgcgcttaatgcCCCAAGTACGTAGCAAGGTGACTCAAACAGAGTACATCCTGCCCGCGTTTCGTATGAATCAAGTTAGAAGTTATGGAACATAATAACATGTGGATGGCCAGTGGTCGGTTGTTACACGCCTGCCGCAACGTTGAAAGTCCCGGATTAGACGTTGATCGGGCACGTAAGAGGTTCCAACTTTCACCATAATGAAATAAGATCACTACCGGGCGTATTTTTTGAGTTATCGAGATTTTCAGGAGCTAAGGAAGCTAAAatggagaaaaaaatcactggatataccaccgttgatatatcccaatggcatcgtaaagaacattttgaggcatttcagtcagttgctcaatgtacctataaccagaccgttcagctggatattacggcctttttaaagaccgtaaagaaaaataagcacaagttttatccggcctttattcacattcttgcccgcctgatgaatgctcatccggaattccgtatggcaatgaaagacggtgagctggtgatatgggatagtgttcacccttgttacaccgttttccatgagcaaactgaaacgttttcatcgctctggagtgaataccacgacgatttccggcagtttctacacatatattcgcaagatgtggcgtgttacggtgaaaacctggcctatttccctaaagggtttattgagaatatgtttttcgtctcagccaatccctgggtgagtttcaccagttttgatttaaacgtggccaatatggacaacttcttcgcccccgttttcacaatgggcaaatattatacgcaaggcgacaaggtgctgatgccgctggcgattcaggttcatcatgccgtTtgtgatggcttccatgtcggcagaatgcttaatgaattacaacagtactgcgatgagtggcagggcggggcgtaactgtcagaccaagtttactcatatatactttagattgatttaaaacttcatttttaatttaaaaggatctaggtgaagatcctttttgataatctcatgaccaaaatcccttaacgtgagttttcgttccactgagcgtcagaccccgtagaaaagatcaaaggatcttcttgagatcctttttttctgcgcgtaatctgctgcttgcaaacaaaaaaaccaccgctaccagcggtggtttgtttgccggatcaagagctaccaactctttttccgaaggtaactggcttcagcagagcgcagataccaaatactgttcttctagtgtagccgtagttaggccaccacttcaagaactctgtagcaccgcctacatacctcgctctgctaatcctgttaccagtggctgctgccagtggcgataagtcgtgtcttaccgggttggactcaagacgatagttaccggataaggcgcagcggtcgggctgaacggggggttcgtgcacacagcccagcttggagcgaacgacctacaccgaactgagatacctacagcgtgagctatgagaaagcgccacgcttcccgaagggagaaaggcggacaggtatccggtaagcggcagggtcggaacaggagagcgcacgagggagcttccagggggaaacgcctggtatctttatagtcctgtcgggtttcgccacctctgacttgagcgtcgatttttgtgatgctcgtcaggggggcggagcctatggaaaaacgccagcaacgcggcctttttacggttcctggccttttgctggccttttgctcacatgacccgacaccatcgaatggcgcaaaacctttcgcggtatggcatgatagcgcccggaagagagtcaattcagggtggtgaatgtgaaaccagtaacgttatacgatgtcgcagagtatgccggtgtctcttatcagaccgtttcccgcgtggtgaaccaggccagccacgtttctgcgaaaacgcgggaaaaagtggaagcggcgatggcggagctgaattacattcccaaccgcgtggcacaacaactggcgggcaaacagtcgttgctgattggcgttgccacctccagtctggccctgcacgcgccgtcgcaaattgtcgcggcgattaaatctcgcgccgatcaactgggtgccagcgtggtggtgtcgatggtagaacgaagcggcgtcgaagcctgtaaagcggcggtgcacaatcttctcgcgcaacgcgtcagtgggctgatcattaactatccgctggatgaccaggatgccattgctgtggaagctgcctgcactaatgttccggcgttatttcttgatgtctctgaccagacacccatcaacagtattattttctcccatgaagacggtacgcgactgggcgtggagcatctggtcgcattgggtcaccagcaaatcgcgctgttagcgggcccattaagttctgtctcggcgcgtctgcgtctggctggctggcataaatatctcactcgcaatcaaattcagccgatagcggaacgggaaggcgactggagtgccatgtccggttttcaacaaaccatgcaaatgctgaatgagggcatcgttcccactgcgatgctggttgccaacgatcagatggcgctgggcgcaatgcgcgccattaccgagtccgggctgcgcgttggtgcggacatctcggtagtgggatacgacgataccgaagacagctcatgttatatcccgccgttaaccaccatcaaacaggattttcgcctgctggggcaaaccagcgtggaccgcttgctgcaactctctcagggccaggcggtgaagggcaatcagctgttgcccgtctcactggtgaaaagaaaaaccaccctggcgcccaatacgcaaaccgcctctccccgcgcgttggccgattcattaatgcagctggcacgacaggtttcccgactggaaagcgggcagtgagcggtacccgataaaagcggcttcctgacaggaggccgttttgttttgcagcccacctcaacgcaattaatgtgagttagctcactcattaggcaccccaggctttacactttatgcttccggctcgtatgttgtgtggaattgtgagcggataacaatttcacacaggaaacagctatgaccatgattacgaatttctagataacgagggcaaatcATGCTTTTATATAAAATGTGTGACAATCAAAATTATGGGGTCACTTACATGAAGTTTTTATTGGCATTTTCGCTTTTAATACCATCCGTGGTTTTTGCAAGTAGTGCAGGTGTTATGACAGGAGCAAAATTCACGCAGATCCAGTTTGGTATGACACGTCAGCAGGTCCTCGACATAGCAGGTGCTGAGAACTGTGAGACTGGTGGATCGTTCGGTGACAGCATCCATTGTCGTGGACATGCAGCAGGAGACTATTATGCATACGCAACCTTCGGCTTCACCAGCGCAGCTGCAGACGCAAAGGTGGATTCGAAAAGCCAGGAAAAACTGCTTGCACCAAGCGCACCAACTCTTACTCTTGCTAAGTTCAACCAAGTCACTGTTGGTATGACTAGAGCACAAGTACTTGCTACCGTCGGACAGGGTTCTTGTACCACTTGGAGTGAGTACTATCCAGCATATCCATCGACGGCAGGAGTGACTCTCAGCCTGTCCTGCTTCGATGTGGACGGTTACTCGTCGACCGGGTTCTACCGAGGCTCGGCGCACCTCTGGTTCACGGACGGGGTGCTTCAGGGCAAGCGGCAGTGGGACCTTGTATAAATGGAAGCCGCGGCCGCGGCGGCACCTCGTCTGGGCGGTGCTACAACTGGGAAGAATTGGAGCCAATCAATTCTTGCGGAGAACTGTGAATGAATTCGCGCAAAGCTTCCCTTGGCAGAACATtgttaaaattcgcgttaaatttttgttaaatcagctcattttttaaccaataggccgaaatcggcaaaatcccttataaatcaaaagaatagaccgagatagggttgagtg

1378 1997 rep_origin

label pMB1

3425 4027 misc_feature

label BLIP

3425 3529 misc_feature

label Sig Sequence

2125 3207 CDS

label LacI

1223 1201 misc_feature

label P317 Cm for

3210 3215 misc_binding

label misc_binding

564 1147 misc_feature

1149 1223

label Cmr

3218 3254 terminator

label tHP_terminator

782 777 misc_feature

label EcoRI

3276 3296 misc_signal

label CAP_binding_site

3311 3316 -35_signal

label -35_signal

3335 3340 -10_signal

label -10_signal

3371 3397 primer_bind

label phiS3

3385 3408 misc_feature

label LacZ

3404 3409 misc_binding

label misc_binding

4037 4044 misc_feature

label NotI

4119 4124 misc_feature

label EcoRI

4056 4078 misc_feature

label PD1 spacer

1 307 rep_origin

label oriF1

3373 3389 misc_feature

label M13R F primer

458 563 misc_feature

label Added Chlor Promoter/RBS

**S7 Fig. Plasmid map of the selection plasmid containing the bacterial toxin and the target site.**
