# Supplementary material for: SMOOT libraries and phage-induced directed evolution of Cas9 to engineer reduced off-target activity
Source: PLoS One. 2020 Apr 16;15(4):e0231716. doi: 10.1371/journal.pone.0231716 (PMC7161989; doi:10.1371/journal.pone.0231716)
Supplement: S8 Fig — (DOCX) [file pone.0231716.s008.docx]

PD1s7 D23A Y128V D1251G T67L - Cas9: ATGGATAAAAAGTATTCTATTGGTTTAGACATCGGCACTAATTCCGTTGGATGGGCTGTCATAACCGCCGAATACAAAGTACCTTCAAAGAAATTTAAGGTGTTGGGGAACACAGACCGTCATTCGATTAAAAAGAATCTTATCGGTGCCCTCCTATTCGATAGTGGCGAAACGGCAGAGGCGACTCGCCTGAAACGACTGGCTCGGAGAAGGTATACACGTCGCAAGAACCGAATATGTTACTTACAAGAAATTTTTAGCAATGAGATGGCCAAAGTTGACGATTCTTTCTTTCACCGTTTGGAAGAGTCCTTCCTTGTCGAAGAGGACAAGAAACATGAACGGCACCCCATCTTTGGAAACATAGTAGATGAGGTGGCAGTGCATGAAAAGTACCCAACGATTTATCACCTCAGAAAAAAGCTAGTTGACTCAACTGATAAAGCGGACCTGAGGTTAATCTACTTGGCTCTTGCCCATATGATAAAGTTCCGTGGGCACTTTCTCATTGAGGGTGATCTAAATCCGGACAACTCGGATGTCGACAAACTGTTCATCCAGTTAGTACAAACCTATAATCAGTTGTTTGAAGAGAACCCTATAAATGCAAGTGGCGTGGATGCGAAGGCTATTCTTAGCGCCCGCCTCTCTAAATCCCGACGGCTAGAAAACCTGATCGCACAATTACCCGGAGAGAAGAAAAATGGGTTGTTCGGTAACCTTATAGCGCTCTCACTAGGCCTGACACCAAATTTTAAGTCGAACTTCGACTTAGCTGAAGATGCCAAATTGCAGCTTAGTAAGGACACGTACGATGACGATCTCGACAATCTACTGGCACAAATTGGAGATCAGTATGCGGACTTATTTTTGGCTGCCAAAAACCTTAGCGATGCAATCCTCCTATCTGACATACTGAGAGTTAATACTGAGATTACCAAGGCGCCGTTATCCGCTTCAATGATCAAAAGGTACGATGAACATCACCAAGACTTGACACTTCTCAAGGCCCTAGTCCGTCAGCAACTGCCTGAGAAATATAAGGAAATATTCTTTGATCAGTCGAAAAACGGGTACGCAGGTTATATTGACGGCGGAGCGAGTCAAGAGGAATTCTACAAGTTTATCAAACCCATATTAGAGAAGATGGATGGGACGGAAGAGTTGCTTGTAAAACTCAATCGCGAAGATCTACTGCGAAAGCAGCGGACTTTCGACAACGGTAGCATTCCACATCAAATCCACTTAGGCGAATTGCATGCTATACTTAGAAGGCAGGAGGATTTTTATCCGTTCCTCAAAGACAATCGTGAAAAGATTGAGAAAATCCTAACCTTTCGCATACCTTACTATGTGGGACCCCTGGCCCGAGGGAACTCTCGGTTCGCATGGATGACAAGAAAGTCCGAAGAAACGATTACTCCATGGAATTTTGAGGAAGTTGTCGATAAAGGTGCGTCAGCTCAATCGTTCATCGAGAGGATGACCAACTTTGACAAGAATTTACCGAACGAAAAAGTATTGCCTAAGCACAGTTTACTTTACGAGTATTTCACAGTGTACAATGAACTCACGAAAGTTAAGTATGTCACTGAGGGCATGCGTAAACCCGCCTTTCTAAGCGGAGAACAGAAGAAAGCAATAGTAGATCTGTTATTCAAGACCAACCGCAAAGTGACAGTTAAGCAATTGAAAGAGGACTACTTTAAGAAAATTGAATGCTTCGATTCTGTCGAGATCTCCGGGGTAGAAGATCGATTTAATGCGTCACTTGGTACGTATCATGACCTCCTAAAGATAATTAAAGATAAGGACTTCCTGGATAACGAAGAGAATGAAGATATCTTAGAAGATATAGTGTTGACTCTTACCCTCTTTGAAGATCGGGAAATGATTGAGGAAAGACTAAAAACATACGCTCACCTGTTCGACGATAAGGTTATGAAACAGTTAAAGAGGCGTCGCTATACGGGCTGGGGACGATTGTCGCGGAAACTTATCAACGGGATAAGAGACAAGCAAAGTGGTAAAACTATTCTCGATTTTCTAAAGAGCGACGGCTTCGCCAATAGGAACTTTATGCAGCTGATCCATGATGACTCTTTAACCTTCAAAGAGGATATACAAAAGGCACAGGTTTCCGGACAAGGGGACTCATTGCACGAACATATTGCGAATCTTGCTGGTTCGCCAGCCATCAAAAAGGGCATACTCCAGACAGTCAAAGTAGTGGATGAGCTAGTTAAGGTCATGGGACGTCACAAACCGGAAAACATTGTAATCGAGATGGCACGCGAAAATCAAACGACTCAGAAGGGGCAAAAAAACAGTCGAGAGCGGATGAAGAGAATAGAAGAGGGTATTAAAGAACTGGGCAGCCAGATCTTAAAGGAGCATCCTGTGGAAAATACCCAATTGCAGAACGAGAAACTTTACCTCTATTACCTACAAAATGGAAGGGACATGTATGTTGATCAGGAACTGGACATAAACCGTTTATCTGATTACGACGTCGATCACATTGTACCCCAATCCTTTTTGAAGGACGATTCAATCGACAATAAAGTGCTTACACGCTCGGATAAGAACCGAGGGAAAAGTGACAATGTTCCAAGCGAGGAAGTCGTAAAGAAAATGAAGAACTATTGGCGGCAGCTCCTAAATGCGAAACTGATAACGCAAAGAAAGTTCGATAACTTAACTAAAGCTGAGAGGGGTGGCTTGTCTGAACTTGACAAGGCCGGATTTATTAAACGTCAGCTCGTGGAAACCCGCCAAATCACAAAGCATGTTGCACAGATACTAGATTCCCGAATGAATACGAAATACGACGAGAACGATAAGCTGATTCGGGAAGTCAAAGTAATCACTTTAAAGTCAAAATTGGTGTCGGACTTCAGAAAGGATTTTCAATTCTATAAAGTTAGGGAGATAAATAACTACCACCATGCGCACGACGCTTATCTTAATGCCGTCGTAGGGACCGCACTCATTAAGAAATACCCGAAGCTAGAAAGTGAGTTTGTGTATGGTGATTACAAAGTTTATGACGTCCGTAAGATGATCGCGAAAAGCGAACAGGAGATAGGCAAGGCTACAGCCAAATACTTCTTTTATTCTAACATTATGAATTTCTTTAAGACGGAAATCACTCTGGCAAACGGAGAGATACGCAAACGACCTTTAATTGAAACCAATGGGGAGACAGGTGAAATCGTATGGGATAAGGGCCGGGACTTCGCGACGGTGAGAAAAGTTTTGTCCATGCCCCAAGTCAACATAGTAAAGAAAACTGAGGTGCAGACCGGAGGGTTTTCAAAGGAATCGATTCTTCCAAAAAGGAATAGTGATAAGCTCATCGCTCGTAAAAAGGACTGGGACCCGAAAAAGTACGGTGGCTTCGATAGCCCTACAGTTGCCTATTCTGTCCTAGTAGTGGCAAAAGTTGAGAAGGGAAAATCCAAGAAACTGAAGTCAGTCAAAGAATTATTGGGGATAACGATTATGGAGCGCTCGTCTTTTGAAAAGAACCCCATCGACTTCCTTGAGGCGAAAGGTTACAAGGAAGTAAAAAAGGATCTCATAATTAAACTACCAAAGTATAGTCTGTTTGAGTTAGAAAATGGCCGAAAACGGATGTTGGCTAGCGCCGGAGAGCTTCAAAAGGGGAACGAACTCGCACTACCGTCTAAATACGTGAATTTCCTGTATTTAGCGTCCCATTACGAGAAGTTGAAAGGTTCACCTGAAGGCAACGAACAGAAGCAACTTTTTGTTGAGCAGCACAAACATTATCTCGACGAAATCATAGAGCAAATTTCGGAATTCAGTAAGAGAGTCATCCTAGCTGATGCCAATCTGGACAAAGTATTAAGCGCATACAACAAGCACAGGGATAAACCCATACGTGAGCAGGCGGAAAATATTATCCATTTGTTTACTCTTACCAACCTCGGCGCTCCAGCCGCATTCAAGTATTTTGACACAACGATAGATCGCAAACGATACACTTCTACCAAGGAGGTGCTAGACGCGACACTGATTCACCAATCCATCACGGGATTATATGAAACTCGGATAGATTTGTCACAGCTTGGGGGTGAC

| Ori_pUC | misc_feature | 7,192 | 7,864 | 673 | forward |
| --- | --- | --- | --- | --- | --- |
| lacI | misc_feature | 6,050 | 7,129 | 1,080 | forward |
| P_lacI | misc_feature | 5,968 | 6,049 | 82 | forward |
| Kanamycin-r | misc_feature | 5,158 | 5,964 | 807 | forward |
| P_Amp | misc_feature | 5,030 | 5,147 | 118 | forward |
| Gly | misc_feature | 4,772 | 4,774 | 3 | forward |
| TAT | Editing History Replacement | 1,043 | 1,045 | 3 | none |
| Cas9 | misc_feature | 662 | 4,765 | 4,104 | forward |
| monopartiteNLS | misc_feature | 641 | 661 | 21 | forward |
| His | misc_feature | 620 | 637 | 18 | forward |
| strong RBS | misc_feature | 602 | 613 | 12 | forward |
| lacO | misc_feature | 527 | 556 | 30 | forward |
| P_T5_Inducible | misc_feature | 439 | 556 | 118 | forward |
| LacO1 | misc_feature | 439 | 459 | 21 | forward |
| Term_bla | misc_feature | 121 | 421 | 301 | forward |
| Term_rpoC | misc_feature | 1 | 120 | 120 | forward |

**S8 Fig. The expression construct used to generate the SpartaCas protein.**
