# Supplementary material for: SMOOT libraries and phage-induced directed evolution of Cas9 to engineer reduced off-target activity
Source: PLoS One. 2020 Apr 16;15(4):e0231716. doi: 10.1371/journal.pone.0231716 (PMC7161989; doi:10.1371/journal.pone.0231716)
Supplement: S1 Table — (DOCX) [file pone.0231716.s010.docx]

**S1 Table. GUIDE-seq results for wildtype Sp.Cas9, eCas, and SpartaCas.**
