# Supplementary material for: SMOOT libraries and phage-induced directed evolution of Cas9 to engineer reduced off-target activity
Source: PLoS One. 2020 Apr 16;15(4):e0231716. doi: 10.1371/journal.pone.0231716 (PMC7161989; doi:10.1371/journal.pone.0231716)
Supplement: S2 Table — (DOCX) [file pone.0231716.s011.docx]

| On Target Sequence |
| --- |
| GTCTGGGCGGTGCTACAACT |

| Off Target Sequences |
| --- |
| AACGGGGCGGTACTACAACT |
| GTCTGGTGGTGCTACAACT |
| ACCTGGACGGTGATACAACC |

**S2 Table.** **The PD1 on target and three off target sequences contained within each selection plasmid used during the initial directed evolution experiments.**
