# Supplementary material for: SMOOT libraries and phage-induced directed evolution of Cas9 to engineer reduced off-target activity
Source: PLoS One. 2020 Apr 16;15(4):e0231716. doi: 10.1371/journal.pone.0231716 (PMC7161989; doi:10.1371/journal.pone.0231716)
Supplement: S3 Table — (DOCX) [file pone.0231716.s012.docx]

| Mutant ID | Mutations relative to wildtype Sp. Cas9 |
| --- | --- |
| Mutant 1 | T67L |
| Mutant 2 | D23A/Y128V |
| Mutant 3: SpartaCas | D23A/T67L/Y128V/D1251G |
| Mutant 4 | N497A/R661A/Q695A/Q926A |
|  |  |

**S3 Table.** **Mutants evaluated in this study.**
