# Supplementary material for: SMOOT libraries and phage-induced directed evolution of Cas9 to engineer reduced off-target activity
Source: PLoS One. 2020 Apr 16;15(4):e0231716. doi: 10.1371/journal.pone.0231716 (PMC7161989; doi:10.1371/journal.pone.0231716)
Supplement: S4 Table — (DOCX) [file pone.0231716.s013.docx]

| Cod Primers on position | Oligo Sequences |
| --- | --- |
| SMOOT For | ataaccccttggggcctctaaac |
| SMOOT Rev | GCTAGTTATTGCTCAGCGG |
| PacBio For | TCCTACCATCCACTCGACACA |
| PacBio Rev | ggcgcgccatgactaacgttttc |
| Mutagenesis codon 1 | ggaggtacccaccNNNgataaaaagtattc |
| Mutagenesis codon 2 | caaaggaggtacccaccatgNNNaaaaagtattcta |
| Mutagenesis codon 3 | ggaggtacccaccatggatNNNaagtattctatt |
| Mutagenesis codon 4 | ggtacccaccatggataaaNNNtattctattggt |
| Mutagenesis codon 5 | cccaccatggataaaaagNNNtctattggtttag |
| Mutagenesis codon 6 | cccaccatggataaaaagtatNNNattggtttagac |
| Mutagenesis codon 7 | aaaaagtattctNNNggtttagacatcggcac |
| Mutagenesis codon 8 | aagtattctattNNNttagacatcggcactaattccgttg |
| Mutagenesis codon 9 | gtattctattggtNNNgacatcggcactaattcc |
| Mutagenesis codon 10 | tctattggtttaNNNatcggcactaattccgttgg |
| Mutagenesis codon 11 | gtattctattggtttagacNNNggcactaattcc |
| Mutagenesis codon 12 | ggtttagacatcNNNactaattccgttggatgg |
| Mutagenesis codon 13 | ggtttagacatcggcNNNaattccgttggat |
| Mutagenesis codon 14 | agacatcggcactNNNtccgttggatgg |
| Mutagenesis codon 15 | atcggcactaatNNNgttggatgggctg |
| Mutagenesis codon 16 | ggcactaattccNNNggatgggctgtcat |
| Mutagenesis codon 17 | cggcactaattccgttNNNtgggctgtcata |
| Mutagenesis codon 18 | ggcactaattccgttggaNNNgctgtcataacc |
| Mutagenesis codon 19 | tccgttggatggNNNgtcataaccgatgaata |
| Mutagenesis codon 20 | ccgttggatgggctNNNataaccgatgaa |
| Mutagenesis codon 21 | ggatgggctgtcNNNaccgatgaatac |
| Mutagenesis codon 22 | gttggatgggctgtcataNNNgatgaatacaaag |
| Mutagenesis codon 23 | gggctgtcataaccNNNgaatacaaagtacct |
| Mutagenesis codon 24 | tgggctgtcataaccgatNNNtacaaagtacct |
| Mutagenesis codon 25 | gctgtcataaccgatgaaNNNaaagtaccttcaaag |
| Mutagenesis codon 26 | tgggctgtcataaccgatgaatacNNNgtaccttcaaaga |
| Mutagenesis codon 27 | gctgtcataaccgatgaatacaaaNNNccttcaaagaaa |
| Mutagenesis codon 28 | ccgatgaatacaaagtaNNNtcaaagaaatttaaggtg |
| Mutagenesis codon 29 | tacaaagtacctNNNaagaaatttaaggtgttgggg |
| Mutagenesis codon 30 | ccgatgaatacaaagtaccttcaNNNaaatttaaggtg |
| Mutagenesis codon 31 | gtaccttcaaagNNNtttaaggtgttggggaac |
| Mutagenesis codon 32 | gtaccttcaaagaaaNNNaaggtgttggggaac |
| Mutagenesis codon 33 | gtaccttcaaagaaatttNNNgtgttggggaac |
| Mutagenesis codon 34 | ccttcaaagaaatttaagNNNttggggaacacag |
| Mutagenesis codon 35 | aaagaaatttaaggtgNNNgggaacacagaccg |
| Mutagenesis codon 36 | caaagaaatttaaggtgttgNNNaacacagaccgtc |
| Mutagenesis codon 37 | aaggtgttggggNNNacagaccgtcatt |
| Mutagenesis codon 38 | ggtgttggggaacNNNgaccgtcattcg |
| Mutagenesis codon 39 | ttggggaacacaNNNcgtcattcgattaaaaag |
| Mutagenesis codon 40 | gggaacacagacNNNcattcgattaaaaaga |
| Mutagenesis codon 41 | tggggaacacagaccgtNNNtcgattaaaaag |
| Mutagenesis codon 42 | ggggaacacagaccgtcatNNNattaaaaagaat |
| Mutagenesis codon 43 | gggaacacagaccgtcattcgNNNaaaaagaatctta |
| Mutagenesis codon 44 | cacagaccgtcattcgattNNNaagaatcttatc |
| Mutagenesis codon 45 | cgtcattcgattaaaNNNaatcttatcggtgccc |
| Mutagenesis codon 46 | ttcgattaaaaagNNNcttatcggtgccctcctatt |
| Mutagenesis codon 47 | cgattaaaaagaatNNNatcggtgccctcctat |
| Mutagenesis codon 48 | aaaaagaatcttNNNggtgccctcctattcg |
| Mutagenesis codon 49 | aaagaatcttatcNNNgccctcctattcg |
| Mutagenesis codon 50 | aatcttatcggtNNNctcctattcgatagtggc |
| Mutagenesis codon 51 | aatcttatcggtgccNNNctattcgatagtg |
| Mutagenesis codon 52 | atcggtgccctcNNNttcgatagtggc |
| Mutagenesis codon 53 | ggtgccctcctaNNNgatagtggcgaaac |
| Mutagenesis codon 54 | gccctcctattcNNNagtggcgaaacg |
| Mutagenesis codon 55 | ctcctattcgatNNNggcgaaacggcag |
| Mutagenesis codon 56 | ctattcgatagtNNNgaaacggcagaggc |
| Mutagenesis codon 57 | ttcgatagtggcNNNacggcagaggcgactc |
| Mutagenesis codon 58 | gatagtggcgaaNNNgcagaggcgactc |
| Mutagenesis codon 59 | agtggcgaaacgNNNgaggcgactcgc |
| Mutagenesis codon 60 | tggcgaaacggcaNNNgcgactcgcctgaaa |
| Mutagenesis codon 61 | ggcgaaacggcagagNNNactcgcctgaaa |
| Mutagenesis codon 62 | acggcagaggcgNNNcgcctgaaacga |
| Mutagenesis codon 63 | gcagaggcgactNNNctgaaacgaacc |
| Mutagenesis codon 64 | gaggcgactcgcNNNaaacgaaccgctc |
| Mutagenesis codon 65 | gcgactcgcctgNNNcgaaccgctcgga |
| Mutagenesis codon 66 | actcgcctgaaaNNNaccgctcggagaag |
| Mutagenesis codon 67 | tcgcctgaaacgaNNNgctcggagaagg |
| Mutagenesis codon 68 | tcgcctgaaacgaaccNNNcggagaaggtatac |
| Mutagenesis codon 69 | aaacgaaccgctNNNagaaggtatacacgtc |
| Mutagenesis codon 70 | cgaaccgctcggNNNaggtatacacgt |
| Mutagenesis codon 71 | accgctcggagaNNNtatacacgtcgcaag |
| Mutagenesis codon 72 | gctcggagaaggNNNacacgtcgcaagaa |
| Mutagenesis codon 73 | cggagaaggtatNNNcgtcgcaagaac |
| Mutagenesis codon 74 | cggagaaggtatacaNNNcgcaagaaccga |
| Mutagenesis codon 75 | cggagaaggtatacacgtNNNaagaaccgaata |
| Mutagenesis codon 76 | aggtatacacgtcgcNNNaaccgaatatgt |
| Mutagenesis codon 77 | acacgtcgcaagNNNcgaatatgttacttacaagaa |
| Mutagenesis codon 78 | acacgtcgcaagaacNNNatatgttacttac |
| Mutagenesis codon 79 | cgtcgcaagaaccgaNNNtgttacttacaaga |
| Mutagenesis codon 80 | cgtcgcaagaaccgaataNNNtacttacaagaa |
| Mutagenesis codon 81 | cgtcgcaagaaccgaatatgtNNNttacaagaaatt |
| Mutagenesis codon 82 | cgcaagaaccgaatatgttacNNNcaagaaatttttagc |
| Mutagenesis codon 83 | cgcaagaaccgaatatgttacttaNNNgaaatttttagc |
| Mutagenesis codon 84 | tgttacttacaaNNNatttttagcaatgagatggccaaag |
| Mutagenesis codon 85 | gttacttacaagaaNNNtttagcaatgagatggcc |
| Mutagenesis codon 86 | cttacaagaaattNNNagcaatgagatggcc |
| Mutagenesis codon 87 | caagaaatttttNNNaatgagatggccaaagttgacg |
| Mutagenesis codon 88 | agaaatttttagcNNNgagatggccaaagttgac |
| Mutagenesis codon 89 | caagaaatttttagcaatNNNatggccaaagttgacg |
| Mutagenesis codon 90 | tttagcaatgagNNNgccaaagttgacgattct |
| Mutagenesis codon 91 | agcaatgagatgNNNaaagttgacgattc |
| Mutagenesis codon 92 | agcaatgagatggccNNNgttgacgattct |
| Mutagenesis codon 93 | agcaatgagatggccaaaNNNgacgattctttc |
| Mutagenesis codon 94 | gatggccaaagttNNNgattctttctttcaccg |
| Mutagenesis codon 95 | tggccaaagttgacNNNtctttctttcacc |
| Mutagenesis codon 96 | ggccaaagttgacgatNNNttctttcaccgttt |
| Mutagenesis codon 97 | gttgacgattctNNNtttcaccgtttggaag |
| Mutagenesis codon 98 | tgacgattctttcNNNcaccgtttggaagag |
| Mutagenesis codon 99 | tgacgattctttctttNNNcgtttggaagagtcctt |
| Mutagenesis codon 100 | cgattctttctttcacNNNttggaagagtcctt |
| Mutagenesis codon 101 | ctttctttcaccgtNNNgaagagtccttcct |
| Mutagenesis codon 102 | ctttcaccgtttgNNNgagtccttccttgt |
| Mutagenesis codon 103 | caccgtttggaaNNNtccttccttgtcgaa |
| Mutagenesis codon 104 | ccgtttggaagagNNNttccttgtcgaag |
| Mutagenesis codon 105 | ccgtttggaagagtccNNNcttgtcgaagag |
| Mutagenesis codon 106 | ggaagagtccttcNNNgtcgaagaggac |
| Mutagenesis codon 107 | agagtccttccttNNNgaagaggacaaga |
| Mutagenesis codon 108 | gtccttccttgtcNNNgaggacaagaaacat |
| Mutagenesis codon 109 | ttccttgtcgaaNNNgacaagaaacatgaacgg |
| Mutagenesis codon 110 | ccttgtcgaagagNNNaagaaacatgaacgg |
| Mutagenesis codon 111 | gtcgaagaggacNNNaaacatgaacggcaccc |
| Mutagenesis codon 112 | cgaagaggacaagNNNcatgaacggcac |
| Mutagenesis codon 113 | gaggacaagaaaNNNgaacggcacccc |
| Mutagenesis codon 114 | ggacaagaaacatNNNcggcaccccatct |
| Mutagenesis codon 115 | gacaagaaacatgaaNNNcaccccatctttgg |
| Mutagenesis codon 116 | acaagaaacatgaacggNNNcccatctttgga |
| Mutagenesis codon 117 | gaaacatgaacggcacNNNatctttggaaacata |
| Mutagenesis codon 118 | gaacggcaccccNNNtttggaaacatagt |
| Mutagenesis codon 119 | cggcaccccatcNNNggaaacatagta |
| Mutagenesis codon 120 | cggcaccccatctttNNNaacatagtagatg |
| Mutagenesis codon 121 | cggcaccccatctttggaNNNatagtagatgag |
| Mutagenesis codon 122 | atctttggaaacNNNgtagatgaggtggcatatc |
| Mutagenesis codon 123 | tttggaaacataNNNgatgaggtggcatatc |
| Mutagenesis codon 124 | tttggaaacatagtaNNNgaggtggcatatc |
| Mutagenesis codon 125 | ggaaacatagtagatNNNgtggcatatcatg |
| Mutagenesis codon 126 | ggaaacatagtagatgagNNNgcatatcatgaaaag |
| Mutagenesis codon 127 | gtagatgaggtgNNNtatcatgaaaagtaccc |
| Mutagenesis codon 128 | gatgaggtggcaNNNcatgaaaagtaccc |
| Mutagenesis codon 129 | tgaggtggcatatNNNgaaaagtacccaacg |
| Mutagenesis codon 130 | gaggtggcatatcatNNNaagtacccaacgattt |
| Mutagenesis codon 131 | ggtggcatatcatgaaNNNtacccaacgatt |
| Mutagenesis codon 132 | gtggcatatcatgaaaagNNNccaacgatttatc |
| Mutagenesis codon 133 | ggcatatcatgaaaagtacNNNacgatttatcacctc |
| Mutagenesis codon 134 | gaaaagtacccaNNNatttatcacctcagaaaaaagc |
| Mutagenesis codon 135 | aagtacccaacgNNNtatcacctcaga |
| Mutagenesis codon 136 | tacccaacgattNNNcacctcagaaaaaagc |
| Mutagenesis codon 137 | cccaacgatttatNNNctcagaaaaaagctagttgac |
| Mutagenesis codon 138 | cccaacgatttatcacNNNagaaaaaagctagttg |
| Mutagenesis codon 139 | cccaacgatttatcacctcNNNaaaaagctagtt |
| Mutagenesis codon 140 | tatcacctcagaNNNaagctagttgactcaactg |
| Mutagenesis codon 141 | tcacctcagaaaaNNNctagttgactcaactga |
| Mutagenesis codon 142 | ctcagaaaaaagNNNgttgactcaactgataaagcg |
| Mutagenesis codon 143 | agaaaaaagctaNNNgactcaactgataaagcggacc |
| Mutagenesis codon 144 | aaaaagctagttNNNtcaactgataaagcggacctg |
| Mutagenesis codon 145 | aagctagttgacNNNactgataaagcggacct |
| Mutagenesis codon 146 | gctagttgactcaNNNgataaagcggacc |
| Mutagenesis codon 147 | gctagttgactcaactNNNaaagcggacctg |
| Mutagenesis codon 148 | gactcaactgatNNNgcggacctgagg |
| Mutagenesis codon 149 | gactcaactgataaaNNNgacctgaggttaatc |
| Mutagenesis codon 150 | actgataaagcgNNNctgaggttaatctacttgg |
| Mutagenesis codon 151 | gactcaactgataaagcggacNNNaggttaatctactt |
| Mutagenesis codon 152 | ctgataaagcggacctgNNNttaatctacttgg |
| Mutagenesis codon 153 | gcggacctgaggNNNatctacttggct |
| Mutagenesis codon 154 | gcggacctgaggttaNNNtacttggctctt |
| Mutagenesis codon 155 | ggacctgaggttaatcNNNttggctcttgcccata |
| Mutagenesis codon 156 | gaggttaatctacNNNgctcttgcccatatgataaa |
| Mutagenesis codon 157 | cctgaggttaatctacttgNNNcttgcccatatg |
| Mutagenesis codon 158 | gaggttaatctacttggctNNNgcccatatgata |
| Mutagenesis codon 159 | ctacttggctcttNNNcatatgataaagttccgtg |
| Mutagenesis codon 160 | cttggctcttgccNNNatgataaagttccg |
| Mutagenesis codon 161 | ggctcttgcccatNNNataaagttccgtg |
| Mutagenesis codon 162 | cttgcccatatgNNNaagttccgtggg |
| Mutagenesis codon 163 | gcccatatgataNNNttccgtgggcac |
| Mutagenesis codon 164 | cccatatgataaagNNNcgtgggcactttct |
| Mutagenesis codon 165 | gcccatatgataaagttcNNNgggcactttctc |
| Mutagenesis codon 166 | gcccatatgataaagttccgtNNNcactttctcatt |
| Mutagenesis codon 167 | aagttccgtgggNNNtttctcattgagggtgat |
| Mutagenesis codon 168 | ttccgtgggcacNNNctcattgagggt |
| Mutagenesis codon 169 | ccgtgggcactttNNNattgagggtgat |
| Mutagenesis codon 170 | gggcactttctcNNNgagggtgatctaaat |
| Mutagenesis codon 171 | gtgggcactttctcattNNNggtgatctaaatc |
| Mutagenesis codon 172 | ggcactttctcattgagNNNgatctaaatccg |
| Mutagenesis codon 173 | cactttctcattgagggtNNNctaaatccggac |
| Mutagenesis codon 174 | cattgagggtgatNNNaatccggacaactc |
| Mutagenesis codon 175 | gagggtgatctaNNNccggacaactcg |
| Mutagenesis codon 176 | gagggtgatctaaatNNNgacaactcggatgt |
| Mutagenesis codon 177 | gggtgatctaaatccgNNNaactcggatgtc |
| Mutagenesis codon 178 | tctaaatccggacNNNtcggatgtcgacaaa |
| Mutagenesis codon 179 | gtgatctaaatccggacaacNNNgatgtcgacaaact |
| Mutagenesis codon 180 | ccggacaactcgNNNgtcgacaaactg |
| Mutagenesis codon 181 | cggacaactcggatNNNgacaaactgttc |
| Mutagenesis codon 182 | cggacaactcggatgtcNNNaaactgttcatc |
| Mutagenesis codon 183 | tcggatgtcgacNNNctgttcatccag |
| Mutagenesis codon 184 | ctcggatgtcgacaaaNNNttcatccagttag |
| Mutagenesis codon 185 | cggatgtcgacaaactgNNNatccagttagta |
| Mutagenesis codon 186 | cgacaaactgttcNNNcagttagtacaaacctat |
| Mutagenesis codon 187 | tgtcgacaaactgttcatcNNNttagtacaaacc |
| Mutagenesis codon 188 | gacaaactgttcatccagNNNgtacaaacctata |
| Mutagenesis codon 189 | cgacaaactgttcatccagttaNNNcaaacctataatc |
| Mutagenesis codon 190 | cgacaaactgttcatccagttagtaNNNacctataatcag |
| Mutagenesis codon 191 | ctgttcatccagttagtacaaNNNtataatcagttg |
| Mutagenesis codon 192 | catccagttagtacaaaccNNNaatcagttgttt |
| Mutagenesis codon 193 | ccagttagtacaaacctatNNNcagttgtttgaagag |
| Mutagenesis codon 194 | catccagttagtacaaacctataatNNNttgtttgaagag |
| Mutagenesis codon 195 | ccagttagtacaaacctataatcagNNNtttgaagagaac |
| Mutagenesis codon 196 | caaacctataatcagttgNNNgaagagaaccct |
| Mutagenesis codon 197 | aatcagttgtttNNNgagaaccctataaatgcaagtggc |
| Mutagenesis codon 198 | cagttgtttgaaNNNaaccctataaatgcaagtggc |
| Mutagenesis codon 199 | cagttgtttgaagagNNNcctataaatgcaagtg |
| Mutagenesis codon 200 | gtttgaagagaacNNNataaatgcaagtggcgtg |
| Mutagenesis codon 201 | gaagagaaccctNNNaatgcaagtggcgtgg |
| Mutagenesis codon 202 | tgaagagaaccctataNNNgcaagtggcgtgga |
| Mutagenesis codon 203 | gaaccctataaatNNNagtggcgtggatgc |
| Mutagenesis codon 204 | cctataaatgcaNNNggcgtggatgcg |
| Mutagenesis codon 205 | ataaatgcaagtNNNgtggatgcgaaggctattc |
| Mutagenesis codon 206 | aatgcaagtggcNNNgatgcgaaggctatt |
| Mutagenesis codon 207 | gcaagtggcgtgNNNgcgaaggctatt |
| Mutagenesis codon 208 | gcaagtggcgtggatNNNaaggctattctt |
| Mutagenesis codon 209 | tggcgtggatgcgNNNgctattcttagc |
| Mutagenesis codon 210 | gcgtggatgcgaagNNNattcttagcgcc |
| Mutagenesis codon 211 | gatgcgaaggctNNNcttagcgcccgc |
| Mutagenesis codon 212 | tgcgaaggctattNNNagcgcccgcctc |
| Mutagenesis codon 213 | aaggctattcttNNNgcccgcctctcta |
| Mutagenesis codon 214 | gctattcttagcNNNcgcctctctaaatcc |
| Mutagenesis codon 215 | ctattcttagcgccNNNctctctaaatccc |
| Mutagenesis codon 216 | ttcttagcgcccgcNNNtctaaatcccgac |
| Mutagenesis codon 217 | agcgcccgcctcNNNaaatcccgacggcta |
| Mutagenesis codon 218 | gcccgcctctctNNNtcccgacggcta |
| Mutagenesis codon 219 | ccgcctctctaaaNNNcgacggctagaa |
| Mutagenesis codon 220 | ccgcctctctaaatccNNNcggctagaaaac |
| Mutagenesis codon 221 | ccgcctctctaaatcccgaNNNctagaaaacctga |
| Mutagenesis codon 222 | aaatcccgacggNNNgaaaacctgatcg |
| Mutagenesis codon 223 | tcccgacggctaNNNaacctgatcgcacaa |
| Mutagenesis codon 224 | cgacggctagaaNNNctgatcgcacaa |
| Mutagenesis codon 225 | cggctagaaaacNNNatcgcacaattaccc |
| Mutagenesis codon 226 | ggctagaaaacctgNNNgcacaattaccc |
| Mutagenesis codon 227 | cggctagaaaacctgatcNNNcaattacccgga |
| Mutagenesis codon 228 | aacctgatcgcaNNNttacccggagaga |
| Mutagenesis codon 229 | cctgatcgcacaaNNNcccggagagaagaa |
| Mutagenesis codon 230 | gatcgcacaattaNNNggagagaagaaaaatggg |
| Mutagenesis codon 231 | cgcacaattacccNNNgagaagaaaaatggg |
| Mutagenesis codon 232 | cgcacaattacccggaNNNaagaaaaatggg |
| Mutagenesis codon 233 | cgcacaattacccggagagNNNaaaaatgggttg |
| Mutagenesis codon 234 | cccggagagaagNNNaatgggttgttc |
| Mutagenesis codon 235 | ggagagaagaaaNNNgggttgttcggtaac |
| Mutagenesis codon 236 | cccggagagaagaaaaatNNNttgttcggtaac |
| Mutagenesis codon 237 | cccggagagaagaaaaatgggNNNttcggtaaccttata |
| Mutagenesis codon 238 | ggagagaagaaaaatgggttgNNNggtaaccttata |
| Mutagenesis codon 239 | aatgggttgttcNNNaaccttatagcgctctca |
| Mutagenesis codon 240 | tgggttgttcggtNNNcttatagcgctctc |
| Mutagenesis codon 241 | gttgttcggtaacNNNatagcgctctcacta |
| Mutagenesis codon 242 | gttcggtaaccttNNNgcgctctcacta |
| Mutagenesis codon 243 | tcggtaaccttataNNNctctcactaggcc |
| Mutagenesis codon 244 | tcggtaaccttatagcgNNNtcactaggcctga |
| Mutagenesis codon 245 | accttatagcgctcNNNctaggcctgacaccaaat |
| Mutagenesis codon 246 | atagcgctctcaNNNggcctgacacca |
| Mutagenesis codon 247 | agcgctctcactaNNNctgacaccaaat |
| Mutagenesis codon 248 | gcgctctcactaggcNNNacaccaaatttt |
| Mutagenesis codon 249 | cgctctcactaggcctgNNNccaaattttaag |
| Mutagenesis codon 250 | ctcactaggcctgacaNNNaattttaagtcg |
| Mutagenesis codon 251 | ggcctgacaccaNNNtttaagtcgaac |
| Mutagenesis codon 252 | ggcctgacaccaaatNNNaagtcgaacttc |
| Mutagenesis codon 253 | gcctgacaccaaattttNNNtcgaacttcgactta |
| Mutagenesis codon 254 | ggcctgacaccaaattttaagNNNaacttcgactta |
| Mutagenesis codon 255 | ccaaattttaagtcgNNNttcgacttagctgaag |
| Mutagenesis codon 256 | tttaagtcgaacNNNgacttagctgaagatgcc |
| Mutagenesis codon 257 | aagtcgaacttcNNNttagctgaagatgccaaa |
| Mutagenesis codon 258 | tcgaacttcgacNNNgctgaagatgcc |
| Mutagenesis codon 259 | agtcgaacttcgacttaNNNgaagatgccaaat |
| Mutagenesis codon 260 | gaacttcgacttagctNNNgatgccaaattgc |
| Mutagenesis codon 261 | cgacttagctgaaNNNgccaaattgcagct |
| Mutagenesis codon 262 | cgacttagctgaagatNNNaaattgcagcttag |
| Mutagenesis codon 263 | gctgaagatgccNNNttgcagcttagt |
| Mutagenesis codon 264 | gctgaagatgccaaaNNNcagcttagtaagg |
| Mutagenesis codon 265 | gctgaagatgccaaattgNNNcttagtaaggac |
| Mutagenesis codon 266 | gccaaattgcagNNNagtaaggacacgtac |
| Mutagenesis codon 267 | tgccaaattgcagcttNNNaaggacacgtac |
| Mutagenesis codon 268 | ttgcagcttagtNNNgacacgtacgatgac |
| Mutagenesis codon 269 | gcagcttagtaagNNNacgtacgatgacgat |
| Mutagenesis codon 270 | gcttagtaaggacNNNtacgatgacgatctc |
| Mutagenesis codon 271 | ttagtaaggacacgNNNgatgacgatctcg |
| Mutagenesis codon 272 | aaggacacgtacNNNgacgatctcgacaa |
| Mutagenesis codon 273 | ggacacgtacgatNNNgatctcgacaatc |
| Mutagenesis codon 274 | cacgtacgatgacNNNctcgacaatctact |
| Mutagenesis codon 275 | gtacgatgacgatNNNgacaatctactggc |
| Mutagenesis codon 276 | cgatgacgatctcNNNaatctactggcacaaat |
| Mutagenesis codon 277 | tgacgatctcgacNNNctactggcacaaat |
| Mutagenesis codon 278 | tgacgatctcgacaatNNNctggcacaaattg |
| Mutagenesis codon 279 | ctcgacaatctaNNNgcacaaattggagatcag |
| Mutagenesis codon 280 | ctcgacaatctactgNNNcaaattggagatcagta |
| Mutagenesis codon 281 | cgacaatctactggcaNNNattggagatcag |
| Mutagenesis codon 282 | tctactggcacaaNNNggagatcagtatgc |
| Mutagenesis codon 283 | ctggcacaaattNNNgatcagtatgcggactt |
| Mutagenesis codon 284 | ggcacaaattggaNNNcagtatgcggactta |
| Mutagenesis codon 285 | ctggcacaaattggagatNNNtatgcggacttat |
| Mutagenesis codon 286 | ggcacaaattggagatcagNNNgcggacttattt |
| Mutagenesis codon 287 | ggagatcagtatNNNgacttatttttggctgcc |
| Mutagenesis codon 288 | tggagatcagtatgcgNNNttatttttggctg |
| Mutagenesis codon 289 | tcagtatgcggacNNNtttttggctgcc |
| Mutagenesis codon 290 | tcagtatgcggacttaNNNttggctgccaaa |
| Mutagenesis codon 291 | tgcggacttatttNNNgctgccaaaaacct |
| Mutagenesis codon 292 | tgcggacttatttttgNNNgccaaaaaccttag |
| Mutagenesis codon 293 | gcggacttatttttggctNNNaaaaaccttagcg |
| Mutagenesis codon 294 | cggacttatttttggctgccNNNaaccttagcgat |
| Mutagenesis codon 295 | ttggctgccaaaNNNcttagcgatgcaatc |
| Mutagenesis codon 296 | gctgccaaaaacNNNagcgatgcaatcc |
| Mutagenesis codon 297 | gctgccaaaaaccttNNNgatgcaatcctc |
| Mutagenesis codon 298 | tgccaaaaaccttagcNNNgcaatcctccta |
| Mutagenesis codon 299 | aaaccttagcgatNNNatcctcctatctgacat |
| Mutagenesis codon 300 | cttagcgatgcaNNNctcctatctgacatac |
| Mutagenesis codon 301 | accttagcgatgcaatcNNNctatctgacatact |
| Mutagenesis codon 302 | gcgatgcaatcctcNNNtctgacatactg |
| Mutagenesis codon 303 | gatgcaatcctcctaNNNgacatactgagag |
| Mutagenesis codon 304 | cgatgcaatcctcctatctNNNatactgagagtt |
| Mutagenesis codon 305 | caatcctcctatctgacNNNctgagagttaatac |
| Mutagenesis codon 306 | cctcctatctgacataNNNagagttaatactgag |
| Mutagenesis codon 307 | cctcctatctgacatactgNNNgttaatactgaga |
| Mutagenesis codon 308 | gacatactgagaNNNaatactgagattaccaaggc |
| Mutagenesis codon 309 | atactgagagttNNNactgagattaccaaggcgccgtt |
| Mutagenesis codon 310 | ctgagagttaatNNNgagattaccaaggcgc |
| Mutagenesis codon 311 | gagagttaatactNNNattaccaaggcgccgtt |
| Mutagenesis codon 312 | gagagttaatactgagNNNaccaaggcgccgttat |
| Mutagenesis codon 313 | aatactgagattNNNaaggcgccgttatcc |
| Mutagenesis codon 314 | actgagattaccNNNgcgccgttatcc |
| Mutagenesis codon 315 | ctgagattaccaagNNNccgttatccgcttcaatgat |
| Mutagenesis codon 316 | ctgagattaccaaggcgNNNttatccgcttcaat |
| Mutagenesis codon 317 | accaaggcgccgNNNtccgcttcaatg |
| Mutagenesis codon 318 | caaggcgccgttaNNNgcttcaatgatcaa |
| Mutagenesis codon 319 | ggcgccgttatccNNNtcaatgatcaaa |
| Mutagenesis codon 320 | cgccgttatccgctNNNatgatcaaaagg |
| Mutagenesis codon 321 | cgccgttatccgcttcaNNNatcaaaaggtac |
| Mutagenesis codon 322 | tccgcttcaatgNNNaaaaggtacgatgaacat |
| Mutagenesis codon 323 | ccgcttcaatgatcNNNaggtacgatgaacat |
| Mutagenesis codon 324 | ccgcttcaatgatcaaaNNNtacgatgaacatc |
| Mutagenesis codon 325 | cgcttcaatgatcaaaaggNNNgatgaacatcac |
| Mutagenesis codon 326 | gatcaaaaggtacNNNgaacatcaccaagactt |
| Mutagenesis codon 327 | caaaaggtacgatNNNcatcaccaagacttg |
| Mutagenesis codon 328 | aaggtacgatgaaNNNcaccaagacttgacac |
| Mutagenesis codon 329 | tacgatgaacatNNNcaagacttgacacttctc |
| Mutagenesis codon 330 | ggtacgatgaacatcacNNNgacttgacactt |
| Mutagenesis codon 331 | gaacatcaccaaNNNttgacacttctcaaggc |
| Mutagenesis codon 332 | gaacatcaccaagacNNNacacttctcaagg |
| Mutagenesis codon 333 | caccaagacttgNNNcttctcaaggcc |
| Mutagenesis codon 334 | caccaagacttgacaNNNctcaaggcccta |
| Mutagenesis codon 335 | agacttgacacttNNNaaggccctagtcc |
| Mutagenesis codon 336 | ttgacacttctcNNNgccctagtccgt |
| Mutagenesis codon 337 | gacacttctcaagNNNctagtccgtcag |
| Mutagenesis codon 338 | acttctcaaggccNNNgtccgtcagcaa |
| Mutagenesis codon 339 | tctcaaggccctaNNNcgtcagcaactg |
| Mutagenesis codon 340 | aaggccctagtcNNNcagcaactgcct |
| Mutagenesis codon 341 | gccctagtccgtNNNcaactgcctgag |
| Mutagenesis codon 342 | cctagtccgtcagNNNctgcctgagaaata |
| Mutagenesis codon 343 | gtccgtcagcaaNNNcctgagaaatataagg |
| Mutagenesis codon 344 | ccgtcagcaactgNNNgagaaatataaggaaata |
| Mutagenesis codon 345 | gtccgtcagcaactgcctNNNaaatataaggaa |
| Mutagenesis codon 346 | ccgtcagcaactgcctgagNNNtataaggaaata |
| Mutagenesis codon 347 | cagcaactgcctgagaaaNNNaaggaaatattc |
| Mutagenesis codon 348 | cagcaactgcctgagaaatatNNNgaaatattctttg |
| Mutagenesis codon 349 | gcctgagaaatataagNNNatattctttgatcagtcg |
| Mutagenesis codon 350 | ctgcctgagaaatataaggaaNNNttctttgatcag |
| Mutagenesis codon 351 | ctgcctgagaaatataaggaaataNNNtttgatcagtcg |
| Mutagenesis codon 352 | aaggaaatattcNNNgatcagtcgaaaaacgggtac |
| Mutagenesis codon 353 | aggaaatattctttNNNcagtcgaaaaacgggtacg |
| Mutagenesis codon 354 | atattctttgatNNNtcgaaaaacgggtacgcagg |
| Mutagenesis codon 355 | ttctttgatcagNNNaaaaacgggtacgcaggtta |
| Mutagenesis codon 356 | ctttgatcagtcgNNNaacgggtacgcaggtt |
| Mutagenesis codon 357 | gatcagtcgaaaNNNgggtacgcaggt |
| Mutagenesis codon 358 | ctttgatcagtcgaaaaacNNNtacgcaggttat |
| Mutagenesis codon 359 | cagtcgaaaaacgggNNNgcaggttatattg |
| Mutagenesis codon 360 | agtcgaaaaacgggtacNNNggttatattgac |
| Mutagenesis codon 361 | aacgggtacgcaNNNtatattgacggcggag |
| Mutagenesis codon 362 | cgggtacgcaggtNNNattgacggcggag |
| Mutagenesis codon 363 | tacgcaggttatNNNgacggcggagcg |
| Mutagenesis codon 364 | gcaggttatattNNNggcggagcgagtc |
| Mutagenesis codon 365 | cgcaggttatattgacNNNggagcgagtcaa |
| Mutagenesis codon 366 | aggttatattgacggcNNNgcgagtcaagag |
| Mutagenesis codon 367 | attgacggcggaNNNagtcaagaggaattc |
| Mutagenesis codon 368 | gacggcggagcgNNNcaagaggaattcta |
| Mutagenesis codon 369 | ggcggagcgagtNNNgaggaattctac |
| Mutagenesis codon 370 | ggcggagcgagtcaaNNNgaattctacaag |
| Mutagenesis codon 371 | ggcggagcgagtcaagagNNNttctacaagtttat |
| Mutagenesis codon 372 | cggcggagcgagtcaagaggaaNNNtacaagtttatc |
| Mutagenesis codon 373 | gcgagtcaagaggaattcNNNaagtttatcaaacccata |
| Mutagenesis codon 374 | gcgagtcaagaggaattctacNNNtttatcaaacccata |
| Mutagenesis codon 375 | gcgagtcaagaggaattctacaagNNNatcaaacccata |
| Mutagenesis codon 376 | gagtcaagaggaattctacaagtttNNNaaacccatatta |
| Mutagenesis codon 377 | gaggaattctacaagtttatcNNNcccatattagag |
| Mutagenesis codon 378 | caagtttatcaaaNNNatattagagaagatggatgggacg |
| Mutagenesis codon 379 | tttatcaaacccNNNttagagaagatggatgggac |
| Mutagenesis codon 380 | atcaaacccataNNNgagaagatggatgggac |
| Mutagenesis codon 381 | aaacccatattaNNNaagatggatgggacggaagag |
| Mutagenesis codon 382 | cccatattagagNNNatggatgggacggaagag |
| Mutagenesis codon 383 | atattagagaagNNNgatgggacggaagagttg |
| Mutagenesis codon 384 | ttagagaagatgNNNgggacggaagagttg |
| Mutagenesis codon 385 | agagaagatggatNNNacggaagagttgcttg |
| Mutagenesis codon 386 | agagaagatggatgggNNNgaagagttgctt |
| Mutagenesis codon 387 | gatggatgggacgNNNgagttgcttgtaaa |
| Mutagenesis codon 388 | gatggatgggacggaaNNNttgcttgtaaaa |
| Mutagenesis codon 389 | gggacggaagagNNNcttgtaaaactcaatc |
| Mutagenesis codon 390 | gggacggaagagttgNNNgtaaaactcaatc |
| Mutagenesis codon 391 | gacggaagagttgcttNNNaaactcaatcgc |
| Mutagenesis codon 392 | gaagagttgcttgtaNNNctcaatcgcgaaga |
| Mutagenesis codon 393 | gacggaagagttgcttgtaaaaNNNaatcgcgaagat |
| Mutagenesis codon 394 | gcttgtaaaactcNNNcgcgaagatctactg |
| Mutagenesis codon 395 | gttgcttgtaaaactcaatNNNgaagatctactgcg |
| Mutagenesis codon 396 | aaactcaatcgcNNNgatctactgcgaaagc |
| Mutagenesis codon 397 | actcaatcgcgaaNNNctactgcgaaagc |
| Mutagenesis codon 398 | caatcgcgaagatNNNctgcgaaagcag |
| Mutagenesis codon 399 | cgcgaagatctaNNNcgaaagcagcgg |
| Mutagenesis codon 400 | cgcgaagatctactgNNNaagcagcggactt |
| Mutagenesis codon 401 | gatctactgcgaNNNcagcggactttc |
| Mutagenesis codon 402 | tctactgcgaaagNNNcggactttcgacaa |
| Mutagenesis codon 403 | ctgcgaaagcagNNNactttcgacaacggta |
| Mutagenesis codon 404 | tgcgaaagcagcggNNNttcgacaacggtag |
| Mutagenesis codon 405 | aagcagcggactNNNgacaacggtagc |
| Mutagenesis codon 406 | cagcggactttcNNNaacggtagcattc |
| Mutagenesis codon 407 | cggactttcgacNNNggtagcattcca |
| Mutagenesis codon 408 | cggactttcgacaacNNNagcattccacatc |
| Mutagenesis codon 409 | gcggactttcgacaacggtNNNattccacatcaaat |
| Mutagenesis codon 410 | cgacaacggtagcNNNccacatcaaatccactta |
| Mutagenesis codon 411 | caacggtagcattNNNcatcaaatccacttagg |
| Mutagenesis codon 412 | acggtagcattccaNNNcaaatccacttagg |
| Mutagenesis codon 413 | acggtagcattccacatNNNatccacttaggc |
| Mutagenesis codon 414 | gtagcattccacatcaaNNNcacttaggcgaa |
| Mutagenesis codon 415 | ccacatcaaatcNNNttaggcgaattgcatgctata |
| Mutagenesis codon 416 | ccacatcaaatccacNNNggcgaattgcat |
| Mutagenesis codon 417 | ccacatcaaatccacttaNNNgaattgcatgcta |
| Mutagenesis codon 418 | ccacatcaaatccacttaggcNNNttgcatgctata |
| Mutagenesis codon 419 | tccacttaggcgaaNNNcatgctatacttagaa |
| Mutagenesis codon 420 | ttaggcgaattgNNNgctatacttagaaggcaggaggatt |
| Mutagenesis codon 421 | ggcgaattgcatNNNatacttagaaggcagg |
| Mutagenesis codon 422 | ggcgaattgcatgctNNNcttagaaggcag |
| Mutagenesis codon 423 | ggcgaattgcatgctataNNNagaaggcaggag |
| Mutagenesis codon 424 | gcatgctatacttNNNaggcaggaggattt |
| Mutagenesis codon 425 | catgctatacttagaNNNcaggaggatttttatccg |
| Mutagenesis codon 426 | gcatgctatacttagaaggNNNgaggatttttatc |
| Mutagenesis codon 427 | catgctatacttagaaggcagNNNgatttttatccg |
| Mutagenesis codon 428 | cttagaaggcaggagNNNttttatccgttcc |
| Mutagenesis codon 429 | aggcaggaggatNNNtatccgttcctc |
| Mutagenesis codon 430 | gcaggaggattttNNNccgttcctcaaag |
| Mutagenesis codon 431 | aggcaggaggatttttatNNNttcctcaaagac |
| Mutagenesis codon 432 | gcaggaggatttttatccgNNNctcaaagacaat |
| Mutagenesis codon 433 | ggaggatttttatccgttcNNNaaagacaatcgtg |
| Mutagenesis codon 434 | ggatttttatccgttcctcNNNgacaatcgtgaaa |
| Mutagenesis codon 435 | tccgttcctcaaaNNNaatcgtgaaaaga |
| Mutagenesis codon 436 | tccgttcctcaaagacNNNcgtgaaaagattg |
| Mutagenesis codon 437 | tccgttcctcaaagacaatNNNgaaaagattgag |
| Mutagenesis codon 438 | ccgttcctcaaagacaatcgtNNNaagattgagaaa |
| Mutagenesis codon 439 | cctcaaagacaatcgtgaaNNNattgagaaaatcc |
| Mutagenesis codon 440 | gacaatcgtgaaaagNNNgagaaaatcctaacc |
| Mutagenesis codon 441 | cgtgaaaagattNNNaaaatcctaacctttcgcatacc |
| Mutagenesis codon 442 | cgtgaaaagattgagNNNatcctaacctttcgcata |
| Mutagenesis codon 443 | cgtgaaaagattgagaaaNNNctaacctttcgcatacc |
| Mutagenesis codon 444 | gattgagaaaatcNNNacctttcgcataccttac |
| Mutagenesis codon 445 | gagaaaatcctaNNNtttcgcataccttactatgtgggac |
| Mutagenesis codon 446 | gagaaaatcctaaccNNNcgcataccttactat |
| Mutagenesis codon 447 | atcctaacctttNNNataccttactatgtgggacc |
| Mutagenesis codon 448 | aatcctaacctttcgcNNNccttactatgtg |
| Mutagenesis codon 449 | ctaacctttcgcataNNNtactatgtgggacc |
| Mutagenesis codon 450 | cctttcgcatacctNNNtatgtgggaccc |
| Mutagenesis codon 451 | tcgcataccttacNNNgtgggacccctg |
| Mutagenesis codon 452 | ataccttactatNNNggacccctggcc |
| Mutagenesis codon 453 | accttactatgtgNNNcccctggcccgag |
| Mutagenesis codon 454 | cttactatgtgggaNNNctggcccgaggga |
| Mutagenesis codon 455 | ctatgtgggacccNNNgcccgagggaact |
| Mutagenesis codon 456 | gtgggacccctgNNNcgagggaactct |
| Mutagenesis codon 457 | ggacccctggccNNNgggaactctcgg |
| Mutagenesis codon 458 | acccctggcccgaNNNaactctcggttc |
| Mutagenesis codon 459 | ctggcccgagggNNNtctcggttcgcat |
| Mutagenesis codon 460 | gcccgagggaacNNNcggttcgcatgga |
| Mutagenesis codon 461 | gcccgagggaactctNNNttcgcatggatg |
| Mutagenesis codon 462 | gagggaactctcggNNNgcatggatgacaa |
| Mutagenesis codon 463 | gggaactctcggttcNNNtggatgacaaga |
| Mutagenesis codon 464 | tctcggttcgcaNNNatgacaagaaagtcc |
| Mutagenesis codon 465 | cggttcgcatggNNNacaagaaagtcc |
| Mutagenesis codon 466 | ttcgcatggatgNNNagaaagtccgaagaaacgatta |
| Mutagenesis codon 467 | cgcatggatgacaNNNaagtccgaagaaac |
| Mutagenesis codon 468 | tcgcatggatgacaagaNNNtccgaagaaacg |
| Mutagenesis codon 469 | cgcatggatgacaagaaagNNNgaagaaacgatta |
| Mutagenesis codon 470 | ggatgacaagaaagtccNNNgaaacgattactc |
| Mutagenesis codon 471 | gacaagaaagtccgaaNNNacgattactccatg |
| Mutagenesis codon 472 | gacaagaaagtccgaagaaNNNattactccatgga |
| Mutagenesis codon 473 | gtccgaagaaacgNNNactccatggaattt |
| Mutagenesis codon 474 | gtccgaagaaacgattNNNccatggaattttg |
| Mutagenesis codon 475 | gtccgaagaaacgattactNNNtggaattttgagg |
| Mutagenesis codon 476 | ccgaagaaacgattactccaNNNaattttgaggaag |
| Mutagenesis codon 477 | cgattactccatggNNNtttgaggaagttgtc |
| Mutagenesis codon 478 | actccatggaatNNNgaggaagttgtcg |
| Mutagenesis codon 479 | ccatggaattttNNNgaagttgtcgataaaggtgc |
| Mutagenesis codon 480 | tggaattttgagNNNgttgtcgataaaggtgcgt |
| Mutagenesis codon 481 | tggaattttgaggaaNNNgtcgataaaggtgcg |
| Mutagenesis codon 482 | tttgaggaagttNNNgataaaggtgcgtcagc |
| Mutagenesis codon 483 | gaggaagttgtcNNNaaaggtgcgtcag |
| Mutagenesis codon 484 | gaagttgtcgatNNNggtgcgtcagctc |
| Mutagenesis codon 485 | gaggaagttgtcgataaaNNNgcgtcagctcaa |
| Mutagenesis codon 486 | tgtcgataaaggtNNNtcagctcaatcgttcatc |
| Mutagenesis codon 487 | cgataaaggtgcgNNNgctcaatcgttcat |
| Mutagenesis codon 488 | aaaggtgcgtcaNNNcaatcgttcatcgag |
| Mutagenesis codon 489 | ggtgcgtcagctNNNtcgttcatcgag |
| Mutagenesis codon 490 | gtgcgtcagctcaaNNNttcatcgagagg |
| Mutagenesis codon 491 | gtcagctcaatcgNNNatcgagaggatga |
| Mutagenesis codon 492 | gctcaatcgttcNNNgagaggatgacc |
| Mutagenesis codon 493 | gctcaatcgttcatcNNNaggatgaccaactt |
| Mutagenesis codon 494 | tcgttcatcgagNNNatgaccaactttgacaagaat |
| Mutagenesis codon 495 | cgttcatcgagaggNNNaccaactttgacaagaat |
| Mutagenesis codon 496 | cgttcatcgagaggatgNNNaactttgacaagaat |
| Mutagenesis codon 497 | cgagaggatgaccNNNtttgacaagaatttacc |
| Mutagenesis codon 498 | gagaggatgaccaacNNNgacaagaatttacc |
| Mutagenesis codon 499 | cgagaggatgaccaactttNNNaagaatttaccg |
| Mutagenesis codon 500 | ggatgaccaactttgacNNNaatttaccgaacgaaa |
| Mutagenesis codon 501 | gaccaactttgacaagNNNttaccgaacgaaa |
| Mutagenesis codon 502 | gaccaactttgacaagaatNNNccgaacgaaaaa |
| Mutagenesis codon 503 | gacaagaatttaNNNaacgaaaaagtattgcctaagcac |
| Mutagenesis codon 504 | gacaagaatttaccgNNNgaaaaagtattgcctaagc |
| Mutagenesis codon 505 | gacaagaatttaccgaacNNNaaagtattgcctaagc |
| Mutagenesis codon 506 | ttaccgaacgaaNNNgtattgcctaagcac |
| Mutagenesis codon 507 | ccgaacgaaaaaNNNttgcctaagcacagtttac |
| Mutagenesis codon 508 | accgaacgaaaaagtaNNNcctaagcacagt |
| Mutagenesis codon 509 | ccgaacgaaaaagtattgNNNaagcacagtttac |
| Mutagenesis codon 510 | cgaaaaagtattgcctNNNcacagtttactttacg |
| Mutagenesis codon 511 | ccgaacgaaaaagtattgcctaagNNNagtttactttac |
| Mutagenesis codon 512 | ttgcctaagcacNNNttactttacgagtatttcac |
| Mutagenesis codon 513 | ttgcctaagcacagtNNNctttacgagtatttc |
| Mutagenesis codon 514 | tgcctaagcacagtttaNNNtacgagtatttcac |
| Mutagenesis codon 515 | gcacagtttacttNNNgagtatttcacagtgtac |
| Mutagenesis codon 516 | gcctaagcacagtttactttacNNNtatttcacagtg |
| Mutagenesis codon 517 | gcacagtttactttacgagNNNttcacagtgtaca |
| Mutagenesis codon 518 | gcacagtttactttacgagtatNNNacagtgtacaatg |
| Mutagenesis codon 519 | gcacagtttactttacgagtatttcNNNgtgtacaatgaa |
| Mutagenesis codon 520 | cgagtatttcacaNNNtacaatgaactcacgaaag |
| Mutagenesis codon 521 | cgagtatttcacagtgNNNaatgaactcacgaaagt |
| Mutagenesis codon 522 | gtatttcacagtgtacNNNgaactcacgaaagt |
| Mutagenesis codon 523 | cacagtgtacaatNNNctcacgaaagttaag |
| Mutagenesis codon 524 | gtgtacaatgaaNNNacgaaagttaagtatgtcactgagg |
| Mutagenesis codon 525 | cacagtgtacaatgaactcNNNaaagttaagtatgtc |
| Mutagenesis codon 526 | cagtgtacaatgaactcacgNNNgttaagtatgtc |
| Mutagenesis codon 527 | gaactcacgaaaNNNaagtatgtcactgaggg |
| Mutagenesis codon 528 | ctcacgaaagttNNNtatgtcactgagggc |
| Mutagenesis codon 529 | cacgaaagttaagNNNgtcactgagggcat |
| Mutagenesis codon 530 | cacgaaagttaagtatNNNactgagggcatgc |
| Mutagenesis codon 531 | aagttaagtatgtcNNNgagggcatgcgtaaac |
| Mutagenesis codon 532 | aagtatgtcactNNNggcatgcgtaaaccc |
| Mutagenesis codon 533 | gtatgtcactgagNNNatgcgtaaacccg |
| Mutagenesis codon 534 | gtcactgagggcNNNcgtaaacccgcctttcta |
| Mutagenesis codon 535 | cactgagggcatgNNNaaacccgccttt |
| Mutagenesis codon 536 | gagggcatgcgtNNNcccgcctttcta |
| Mutagenesis codon 537 | gggcatgcgtaaaNNNgcctttctaagc |
| Mutagenesis codon 538 | gggcatgcgtaaacccNNNtttctaagcgga |
| Mutagenesis codon 539 | cgtaaacccgccNNNctaagcggagaa |
| Mutagenesis codon 540 | aaacccgcctttNNNagcggagaacag |
| Mutagenesis codon 541 | cccgcctttctaNNNggagaacagaag |
| Mutagenesis codon 542 | cgcctttctaagcNNNgaacagaagaaagc |
| Mutagenesis codon 543 | cgcctttctaagcggaNNNcagaagaaagcaatag |
| Mutagenesis codon 544 | ctaagcggagaaNNNaagaaagcaatagtagatc |
| Mutagenesis codon 545 | agcggagaacagNNNaaagcaatagtagatctg |
| Mutagenesis codon 546 | agcggagaacagaagNNNgcaatagtagat |
| Mutagenesis codon 547 | agcggagaacagaagaaaNNNatagtagatctg |
| Mutagenesis codon 548 | gcggagaacagaagaaagcaNNNgtagatctgtta |
| Mutagenesis codon 549 | gcggagaacagaagaaagcaataNNNgatctgttattc |
| Mutagenesis codon 550 | aaagcaatagtaNNNctgttattcaagaccaaccgc |
| Mutagenesis codon 551 | gcaatagtagatNNNttattcaagaccaaccgc |
| Mutagenesis codon 552 | gcaatagtagatctgNNNttcaagaccaaccgcaaa |
| Mutagenesis codon 553 | gtagatctgttaNNNaagaccaaccgcaaagtg |
| Mutagenesis codon 554 | gatctgttattcNNNaccaaccgcaaagtgaca |
| Mutagenesis codon 555 | ctgttattcaagNNNaaccgcaaagtgacagtt |
| Mutagenesis codon 556 | ctgttattcaagaccNNNcgcaaagtgacagtt |
| Mutagenesis codon 557 | ttcaagaccaacNNNaaagtgacagttaagc |
| Mutagenesis codon 558 | aagaccaaccgcNNNgtgacagttaagc |
| Mutagenesis codon 559 | caagaccaaccgcaaaNNNacagttaagcaa |
| Mutagenesis codon 560 | aaccgcaaagtgNNNgttaagcaattgaaagagg |
| Mutagenesis codon 561 | ccgcaaagtgacaNNNaagcaattgaaagagg |
| Mutagenesis codon 562 | accgcaaagtgacagttNNNcaattgaaagag |
| Mutagenesis codon 563 | cgcaaagtgacagttaagNNNttgaaagaggac |
| Mutagenesis codon 564 | ccgcaaagtgacagttaagcaaNNNaaagaggactac |
| Mutagenesis codon 565 | gtgacagttaagcaattgNNNgaggactacttta |
| Mutagenesis codon 566 | gtgacagttaagcaattgaaaNNNgactactttaag |
| Mutagenesis codon 567 | gtgacagttaagcaattgaaagagNNNtactttaagaaa |
| Mutagenesis codon 568 | gcaattgaaagaggacNNNtttaagaaaattgaatgcttc |
| Mutagenesis codon 569 | gaaagaggactacNNNaagaaaattgaatgcttcg |
| Mutagenesis codon 570 | gaggactactttNNNaaaattgaatgcttcgattctgtcg |
| Mutagenesis codon 571 | gaggactactttaagNNNattgaatgcttcg |
| Mutagenesis codon 572 | gaggactactttaagaaaNNNgaatgcttcgattc |
| Mutagenesis codon 573 | ggactactttaagaaaattNNNtgcttcgattctgtcg |
| Mutagenesis codon 574 | aagaaaattgaaNNNttcgattctgtcgagatctccgg |
| Mutagenesis codon 575 | gaaaattgaatgcNNNgattctgtcgagatctcc |
| Mutagenesis codon 576 | attgaatgcttcNNNtctgtcgagatctccg |
| Mutagenesis codon 577 | gaatgcttcgatNNNgtcgagatctccg |
| Mutagenesis codon 578 | tgcttcgattctNNNgagatctccggg |
| Mutagenesis codon 579 | gcttcgattctgtcNNNatctccggggta |
| Mutagenesis codon 580 | cgattctgtcgagNNNtccggggtagaa |
| Mutagenesis codon 581 | tctgtcgagatcNNNggggtagaagatc |
| Mutagenesis codon 582 | tgtcgagatctccNNNgtagaagatcgattta |
| Mutagenesis codon 583 | cgagatctccgggNNNgaagatcgattta |
| Mutagenesis codon 584 | cgagatctccggggtaNNNgatcgatttaat |
| Mutagenesis codon 585 | ctccggggtagaaNNNcgatttaatgcg |
| Mutagenesis codon 586 | ccggggtagaagatNNNtttaatgcgtcact |
| Mutagenesis codon 587 | gggtagaagatcgaNNNaatgcgtcacttg |
| Mutagenesis codon 588 | gaagatcgatttNNNgcgtcacttggtacg |
| Mutagenesis codon 589 | gggtagaagatcgatttaatNNNtcacttggtacg |
| Mutagenesis codon 590 | cgatttaatgcgNNNcttggtacgtatcatgac |
| Mutagenesis codon 591 | tttaatgcgtcaNNNggtacgtatcatgacc |
| Mutagenesis codon 592 | aatgcgtcacttNNNacgtatcatgacctcc |
| Mutagenesis codon 593 | gcgtcacttggtNNNtatcatgacctcct |
| Mutagenesis codon 594 | tcacttggtacgNNNcatgacctcctaaag |
| Mutagenesis codon 595 | gcgtcacttggtacgtatNNNgacctcctaaag |
| Mutagenesis codon 596 | cgtcacttggtacgtatcatNNNctcctaaagataa |
| Mutagenesis codon 597 | gcgtcacttggtacgtatcatgacNNNctaaagataatt |
| Mutagenesis codon 598 | cgtatcatgacctcNNNaagataattaaagataaggac |
| Mutagenesis codon 599 | catgacctcctaNNNataattaaagataaggacttcc |
| Mutagenesis codon 600 | gacctcctaaagNNNattaaagataaggacttcc |
| Mutagenesis codon 601 | cctcctaaagataNNNaaagataaggacttcctgg |
| Mutagenesis codon 602 | gacctcctaaagataattNNNgataaggacttcc |
| Mutagenesis codon 603 | cctcctaaagataattaaaNNNaaggacttcctgg |
| Mutagenesis codon 604 | gacctcctaaagataattaaagatNNNgacttcctggat |
| Mutagenesis codon 605 | cctcctaaagataattaaagataagNNNttcctggataac |
| Mutagenesis codon 606 | aaagataaggacNNNctggataacgaagagaatg |
| Mutagenesis codon 607 | gataaggacttcNNNgataacgaagagaatgaag |
| Mutagenesis codon 608 | aagataaggacttcctgNNNaacgaagagaatga |
| Mutagenesis codon 609 | ggacttcctggatNNNgaagagaatgaagatatc |
| Mutagenesis codon 610 | ggacttcctggataacNNNgagaatgaagatatc |
| Mutagenesis codon 611 | ggacttcctggataacgaaNNNaatgaagatatc |
| Mutagenesis codon 612 | cctggataacgaagagNNNgaagatatcttagaag |
| Mutagenesis codon 613 | cctggataacgaagagaatNNNgatatcttagaag |
| Mutagenesis codon 614 | cctggataacgaagagaatgaaNNNatcttagaagat |
| Mutagenesis codon 615 | cgaagagaatgaagatNNNttagaagatatagtgttgac |
| Mutagenesis codon 616 | cgaagagaatgaagatatcNNNgaagatatagtgttgac |
| Mutagenesis codon 617 | cgaagagaatgaagatatcttaNNNgatatagtgttgac |
| Mutagenesis codon 618 | cgaagagaatgaagatatcttagaaNNNatagtgttgact |
| Mutagenesis codon 619 | gatatcttagaagatNNNgtgttgactcttaccc |
| Mutagenesis codon 620 | cttagaagatataNNNttgactcttaccctctttgaag |
| Mutagenesis codon 621 | gaagatatagtgNNNactcttaccctctttg |
| Mutagenesis codon 622 | cttagaagatatagtgttgNNNcttaccctctttg |
| Mutagenesis codon 623 | atagtgttgactNNNaccctctttgaagatcg |
| Mutagenesis codon 624 | agtgttgactcttNNNctctttgaagatcggg |
| Mutagenesis codon 625 | gtgttgactcttaccNNNtttgaagatcgggaaa |
| Mutagenesis codon 626 | gactcttaccctcNNNgaagatcgggaaat |
| Mutagenesis codon 627 | gactcttaccctctttNNNgatcgggaaatg |
| Mutagenesis codon 628 | cttaccctctttgaaNNNcgggaaatgattgag |
| Mutagenesis codon 629 | accctctttgaagatNNNgaaatgattgagg |
| Mutagenesis codon 630 | ctttgaagatcggNNNatgattgaggaaagacta |
| Mutagenesis codon 631 | gaagatcgggaaNNNattgaggaaagactaa |
| Mutagenesis codon 632 | tttgaagatcgggaaatgNNNgaggaaagacta |
| Mutagenesis codon 633 | cgggaaatgattNNNgaaagactaaaaacatacgc |
| Mutagenesis codon 634 | cgggaaatgattgagNNNagactaaaaacatacgc |
| Mutagenesis codon 635 | tcgggaaatgattgaggaaNNNctaaaaacatacgc |
| Mutagenesis codon 636 | cgggaaatgattgaggaaagaNNNaaaacatacgct |
| Mutagenesis codon 637 | gaggaaagactaNNNacatacgctcacctg |
| Mutagenesis codon 638 | tgaggaaagactaaaaNNNtacgctcacctg |
| Mutagenesis codon 639 | gaggaaagactaaaaacaNNNgctcacctgttc |
| Mutagenesis codon 640 | aggaaagactaaaaacatacNNNcacctgttcgac |
| Mutagenesis codon 641 | aaaacatacgctNNNctgttcgacgataagg |
| Mutagenesis codon 642 | acatacgctcacNNNttcgacgataag |
| Mutagenesis codon 643 | tacgctcacctgNNNgacgataaggttatg |
| Mutagenesis codon 644 | cgctcacctgttcNNNgataaggttatgaaac |
| Mutagenesis codon 645 | cgctcacctgttcgacNNNaaggttatgaaa |
| Mutagenesis codon 646 | tcacctgttcgacgatNNNgttatgaaacagtt |
| Mutagenesis codon 647 | cacctgttcgacgataagNNNatgaaacagtta |
| Mutagenesis codon 648 | cgacgataaggttNNNaaacagttaaagaggcg |
| Mutagenesis codon 649 | cgacgataaggttatgNNNcagttaaagaggc |
| Mutagenesis codon 650 | cgacgataaggttatgaaaNNNttaaagaggcgtcg |
| Mutagenesis codon 651 | aggttatgaaacagNNNaagaggcgtcgctat |
| Mutagenesis codon 652 | atgaaacagttaNNNaggcgtcgctatacgg |
| Mutagenesis codon 653 | aggttatgaaacagttaaagNNNcgtcgctatacg |
| Mutagenesis codon 654 | acagttaaagaggNNNcgctatacgggc |
| Mutagenesis codon 655 | ttaaagaggcgtNNNtatacgggctggg |
| Mutagenesis codon 656 | aagaggcgtcgcNNNacgggctggggac |
| Mutagenesis codon 657 | aggcgtcgctatNNNggctggggacga |
| Mutagenesis codon 658 | cgtcgctatacgNNNtggggacgattgt |
| Mutagenesis codon 659 | cgctatacgggcNNNggacgattgtcg |
| Mutagenesis codon 660 | cgctatacgggctggNNNcgattgtcgcggaaacttat |
| Mutagenesis codon 661 | acgggctggggaNNNttgtcgcggaaa |
| Mutagenesis codon 662 | cgggctggggacgaNNNtcgcggaaactta |
| Mutagenesis codon 663 | ctggggacgattgNNNcggaaacttatc |
| Mutagenesis codon 664 | ggacgattgtcgNNNaaacttatcaacgggataa |
| Mutagenesis codon 665 | acgattgtcgcggNNNcttatcaacggg |
| Mutagenesis codon 666 | ttgtcgcggaaaNNNatcaacgggataagag |
| Mutagenesis codon 667 | gtcgcggaaacttNNNaacgggataagag |
| Mutagenesis codon 668 | cgcggaaacttatcNNNgggataagagacaa |
| Mutagenesis codon 669 | gcggaaacttatcaacNNNataagagacaagcaaag |
| Mutagenesis codon 670 | cgcggaaacttatcaacgggNNNagagacaagcaaag |
| Mutagenesis codon 671 | atcaacgggataNNNgacaagcaaagtggtaaa |
| Mutagenesis codon 672 | caacgggataagaNNNaagcaaagtggtaaaac |
| Mutagenesis codon 673 | cgggataagagacNNNcaaagtggtaaaactattc |
| Mutagenesis codon 674 | acgggataagagacaagNNNagtggtaaaactattc |
| Mutagenesis codon 675 | acgggataagagacaagcaaNNNggtaaaactattc |
| Mutagenesis codon 676 | cgggataagagacaagcaaagtNNNaaaactattctc |
| Mutagenesis codon 677 | gagacaagcaaagtggtNNNactattctcgattt |
| Mutagenesis codon 678 | gagacaagcaaagtggtaaaNNNattctcgattttc |
| Mutagenesis codon 679 | gacaagcaaagtggtaaaactNNNctcgattttcta |
| Mutagenesis codon 680 | gtggtaaaactattNNNgattttctaaagagcgacgg |
| Mutagenesis codon 681 | gtggtaaaactattctcNNNtttctaaagagcgacg |
| Mutagenesis codon 682 | aaactattctcgatNNNctaaagagcgacggc |
| Mutagenesis codon 683 | attctcgattttNNNaagagcgacggcttcg |
| Mutagenesis codon 684 | ctcgattttctaNNNagcgacggcttcg |
| Mutagenesis codon 685 | gattttctaaagNNNgacggcttcgccaatag |
| Mutagenesis codon 686 | cgattttctaaagagcNNNggcttcgccaata |
| Mutagenesis codon 687 | tttctaaagagcgacNNNttcgccaatagg |
| Mutagenesis codon 688 | aagagcgacggcNNNgccaataggaactt |
| Mutagenesis codon 689 | gagcgacggcttcNNNaataggaacttt |
| Mutagenesis codon 690 | cgacggcttcgccNNNaggaactttatg |
| Mutagenesis codon 691 | cgacggcttcgccaatNNNaactttatgcag |
| Mutagenesis codon 692 | ggcttcgccaataggNNNtttatgcagctgat |
| Mutagenesis codon 693 | cgccaataggaacNNNatgcagctgatc |
| Mutagenesis codon 694 | cgccaataggaactttNNNcagctgatccat |
| Mutagenesis codon 695 | cgccaataggaactttatgNNNctgatccatgat |
| Mutagenesis codon 696 | gccaataggaactttatgcagNNNatccatgatgac |
| Mutagenesis codon 697 | ggaactttatgcagctgNNNcatgatgactcttta |
| Mutagenesis codon 698 | ggaactttatgcagctgatcNNNgatgactcttta |
| Mutagenesis codon 699 | gcagctgatccatNNNgactctttaacc |
| Mutagenesis codon 700 | gcagctgatccatgatNNNtctttaaccttc |
| Mutagenesis codon 701 | gcagctgatccatgatgacNNNttaaccttcaaa |
| Mutagenesis codon 702 | ccatgatgactctNNNaccttcaaagagg |
| Mutagenesis codon 703 | gctgatccatgatgactctttaNNNttcaaagaggat |
| Mutagenesis codon 704 | ccatgatgactctttaaccNNNaaagaggatatac |
| Mutagenesis codon 705 | ccatgatgactctttaaccttcNNNgaggatatacaa |
| Mutagenesis codon 706 | ttaaccttcaaaNNNgatatacaaaaggcacaggtttcc |
| Mutagenesis codon 707 | aaccttcaaagagNNNatacaaaaggcacagg |
| Mutagenesis codon 708 | ccttcaaagaggatNNNcaaaaggcacagg |
| Mutagenesis codon 709 | ccttcaaagaggatataNNNaaggcacaggtt |
| Mutagenesis codon 710 | caaagaggatatacaaNNNgcacaggtttccg |
| Mutagenesis codon 711 | caaagaggatatacaaaagNNNcaggtttccgga |
| Mutagenesis codon 712 | atacaaaaggcaNNNgtttccggacaaggg |
| Mutagenesis codon 713 | caaaaggcacagNNNtccggacaaggg |
| Mutagenesis codon 714 | aaggcacaggttNNNggacaaggggactc |
| Mutagenesis codon 715 | gcacaggtttccNNNcaaggggactcatt |
| Mutagenesis codon 716 | caggtttccggaNNNggggactcattg |
| Mutagenesis codon 717 | caggtttccggacaaNNNgactcattgcac |
| Mutagenesis codon 718 | tccggacaagggNNNtcattgcacgaa |
| Mutagenesis codon 719 | cggacaaggggacNNNttgcacgaacata |
| Mutagenesis codon 720 | caaggggactcaNNNcacgaacatattgc |
| Mutagenesis codon 721 | ggggactcattgNNNgaacatattgcgaatct |
| Mutagenesis codon 722 | gggactcattgcacNNNcatattgcgaatct |
| Mutagenesis codon 723 | gggactcattgcacgaaNNNattgcgaatctt |
| Mutagenesis codon 724 | ttgcacgaacatNNNgcgaatcttgctggtt |
| Mutagenesis codon 725 | gcacgaacatattNNNaatcttgctggttcgc |
| Mutagenesis codon 726 | cgaacatattgcgNNNcttgctggttcg |
| Mutagenesis codon 727 | catattgcgaatNNNgctggttcgccag |
| Mutagenesis codon 728 | attgcgaatcttNNNggttcgccagcc |
| Mutagenesis codon 729 | tgcgaatcttgctNNNtcgccagccatc |
| Mutagenesis codon 730 | cgaatcttgctggtNNNccagccatcaaa |
| Mutagenesis codon 731 | cttgctggttcgNNNgccatcaaaaagg |
| Mutagenesis codon 732 | gctggttcgccaNNNatcaaaaagggcata |
| Mutagenesis codon 733 | ggttcgccagccNNNaaaaagggcatac |
| Mutagenesis codon 734 | tcgccagccatcNNNaagggcatactc |
| Mutagenesis codon 735 | ccagccatcaaaNNNggcatactccagaca |
| Mutagenesis codon 736 | gccatcaaaaagNNNatactccagacagtcaaagt |
| Mutagenesis codon 737 | ccatcaaaaagggcNNNctccagacagtcaaa |
| Mutagenesis codon 738 | cagccatcaaaaagggcataNNNcagacagtcaaa |
| Mutagenesis codon 739 | cagccatcaaaaagggcatactcNNNacagtcaaagta |
| Mutagenesis codon 740 | gggcatactccagNNNgtcaaagtagtg |
| Mutagenesis codon 741 | gggcatactccagacaNNNaaagtagtggatga |
| Mutagenesis codon 742 | ctccagacagtcNNNgtagtggatgag |
| Mutagenesis codon 743 | ccagacagtcaaaNNNgtggatgagctagt |
| Mutagenesis codon 744 | cagacagtcaaagtaNNNgatgagctagttaagg |
| Mutagenesis codon 745 | cagacagtcaaagtagtgNNNgagctagttaag |
| Mutagenesis codon 746 | caaagtagtggatNNNctagttaaggtcatggg |
| Mutagenesis codon 747 | gtagtggatgagNNNgttaaggtcatggg |
| Mutagenesis codon 748 | gtggatgagctaNNNaaggtcatgggac |
| Mutagenesis codon 749 | ggatgagctagttNNNgtcatgggacgtcac |
| Mutagenesis codon 750 | ggatgagctagttaagNNNatgggacgtcacaaa |
| Mutagenesis codon 751 | gagctagttaaggtcNNNggacgtcacaaa |
| Mutagenesis codon 752 | ctagttaaggtcatgNNNcgtcacaaaccg |
| Mutagenesis codon 753 | aaggtcatgggaNNNcacaaaccggaaaac |
| Mutagenesis codon 754 | ggtcatgggacgtNNNaaaccggaaaac |
| Mutagenesis codon 755 | atgggacgtcacNNNccggaaaacattgta |
| Mutagenesis codon 756 | ggtcatgggacgtcacaaaNNNgaaaacattgta |
| Mutagenesis codon 757 | gggacgtcacaaaccgNNNaacattgtaatc |
| Mutagenesis codon 758 | cgtcacaaaccggaaNNNattgtaatcgagatg |
| Mutagenesis codon 759 | gtcacaaaccggaaaacNNNgtaatcgagatg |
| Mutagenesis codon 760 | accggaaaacattNNNatcgagatggcacgc |
| Mutagenesis codon 761 | cggaaaacattgtaNNNgagatggcacgc |
| Mutagenesis codon 762 | ccggaaaacattgtaatcNNNatggcacgcgaaa |
| Mutagenesis codon 763 | attgtaatcgagNNNgcacgcgaaaatcaaacg |
| Mutagenesis codon 764 | cattgtaatcgagatgNNNcgcgaaaatcaaacg |
| Mutagenesis codon 765 | aatcgagatggcaNNNgaaaatcaaacgactc |
| Mutagenesis codon 766 | gagatggcacgcNNNaatcaaacgactca |
| Mutagenesis codon 767 | atggcacgcgaaNNNcaaacgactcagaa |
| Mutagenesis codon 768 | gcacgcgaaaatNNNacgactcagaagg |
| Mutagenesis codon 769 | acgcgaaaatcaaNNNactcagaaggggcaaa |
| Mutagenesis codon 770 | gcgaaaatcaaacgNNNcagaaggggcaaa |
| Mutagenesis codon 771 | acgcgaaaatcaaacgactNNNaaggggcaaaaa |
| Mutagenesis codon 772 | atcaaacgactcagNNNgggcaaaaaaacag |
| Mutagenesis codon 773 | acgactcagaagNNNcaaaaaaacagtcgagag |
| Mutagenesis codon 774 | actcagaaggggNNNaaaaacagtcgagag |
| Mutagenesis codon 775 | cagaaggggcaaNNNaacagtcgagag |
| Mutagenesis codon 776 | aaggggcaaaaaNNNagtcgagagcggatga |
| Mutagenesis codon 777 | gggcaaaaaaacNNNcgagagcggatgaa |
| Mutagenesis codon 778 | gggcaaaaaaacagtNNNgagcggatgaag |
| Mutagenesis codon 779 | ggcaaaaaaacagtcgaNNNcggatgaagagaa |
| Mutagenesis codon 780 | aacagtcgagagNNNatgaagagaatagaagagggtat |
| Mutagenesis codon 781 | agtcgagagcggNNNaagagaatagaagagggtat |
| Mutagenesis codon 782 | cgagagcggatgNNNagaatagaagagggtat |
| Mutagenesis codon 783 | cgagagcggatgaagNNNatagaagagggtat |
| Mutagenesis codon 784 | gagcggatgaagagaNNNgaagagggtatta |
| Mutagenesis codon 785 | gagcggatgaagagaataNNNgagggtattaaag |
| Mutagenesis codon 786 | gagcggatgaagagaatagaaNNNggtattaaagaac |
| Mutagenesis codon 787 | gcggatgaagagaatagaagagNNNattaaagaactgg |
| Mutagenesis codon 788 | atagaagagggtNNNaaagaactgggcagcc |
| Mutagenesis codon 789 | gaagagggtattNNNgaactgggcagcc |
| Mutagenesis codon 790 | gagggtattaaaNNNctgggcagccaga |
| Mutagenesis codon 791 | gagggtattaaagaaNNNggcagccagatc |
| Mutagenesis codon 792 | gaagagggtattaaagaactgNNNagccagatctta |
| Mutagenesis codon 793 | gggtattaaagaactgggcNNNcagatcttaaag |
| Mutagenesis codon 794 | gaactgggcagcNNNatcttaaaggagcat |
| Mutagenesis codon 795 | ctgggcagccagNNNttaaaggagcat |
| Mutagenesis codon 796 | gggcagccagatcNNNaaggagcatcct |
| Mutagenesis codon 797 | agccagatcttaNNNgagcatcctgtggaaa |
| Mutagenesis codon 798 | gccagatcttaaagNNNcatcctgtggaa |
| Mutagenesis codon 799 | gccagatcttaaaggagNNNcctgtggaaaata |
| Mutagenesis codon 800 | ccagatcttaaaggagcatNNNgtggaaaatacc |
| Mutagenesis codon 801 | aaaggagcatcctNNNgaaaatacccaattgc |
| Mutagenesis codon 802 | ggagcatcctgtgNNNaatacccaattg |
| Mutagenesis codon 803 | catcctgtggaaNNNacccaattgcagaacgagaaa |
| Mutagenesis codon 804 | gagcatcctgtggaaaatNNNcaattgcagaac |
| Mutagenesis codon 805 | cctgtggaaaataccNNNttgcagaacgaga |
| Mutagenesis codon 806 | cctgtggaaaatacccaaNNNcagaacgagaaa |
| Mutagenesis codon 807 | cctgtggaaaatacccaattgNNNaacgagaaactt |
| Mutagenesis codon 808 | gtggaaaatacccaattgcagNNNgagaaactttac |
| Mutagenesis codon 809 | acccaattgcagaacNNNaaactttacctct |
| Mutagenesis codon 810 | cccaattgcagaacgagNNNctttacctctatta |
| Mutagenesis codon 811 | gcagaacgagaaaNNNtacctctattacctac |
| Mutagenesis codon 812 | gcagaacgagaaacttNNNctctattacctac |
| Mutagenesis codon 813 | gcagaacgagaaactttacNNNtattacctacaa |
| Mutagenesis codon 814 | gcagaacgagaaactttacctcNNNtacctacaaaat |
| Mutagenesis codon 815 | cgagaaactttacctctatNNNctacaaaatggaagg |
| Mutagenesis codon 816 | cgagaaactttacctctattacNNNcaaaatggaagg |
| Mutagenesis codon 817 | cctctattacctaNNNaatggaagggacatg |
| Mutagenesis codon 818 | cctctattacctacaaNNNggaagggacatg |
| Mutagenesis codon 819 | cctctattacctacaaaatNNNagggacatgtatg |
| Mutagenesis codon 820 | ctacaaaatggaNNNgacatgtatgttgatcaggaac |
| Mutagenesis codon 821 | caaaatggaaggNNNatgtatgttgatcaggaactg |
| Mutagenesis codon 822 | cctacaaaatggaagggacNNNtatgttgatcag |
| Mutagenesis codon 823 | ggaagggacatgNNNgttgatcaggaac |
| Mutagenesis codon 824 | ggaagggacatgtatNNNgatcaggaactg |
| Mutagenesis codon 825 | gggacatgtatgttNNNcaggaactggac |
| Mutagenesis codon 826 | ggaagggacatgtatgttgatNNNgaactggacataa |
| Mutagenesis codon 827 | catgtatgttgatcagNNNctggacataaaccg |
| Mutagenesis codon 828 | gttgatcaggaaNNNgacataaaccgtttatc |
| Mutagenesis codon 829 | gttgatcaggaactgNNNataaaccgtttatctg |
| Mutagenesis codon 830 | gatcaggaactggacNNNaaccgtttatct |
| Mutagenesis codon 831 | caggaactggacataNNNcgtttatctgattacg |
| Mutagenesis codon 832 | ggaactggacataaacNNNttatctgattacgac |
| Mutagenesis codon 833 | gacataaaccgtNNNtctgattacgacgtcg |
| Mutagenesis codon 834 | ggacataaaccgtttaNNNgattacgacgtcg |
| Mutagenesis codon 835 | aaccgtttatctNNNtacgacgtcgatcac |
| Mutagenesis codon 836 | accgtttatctgatNNNgacgtcgatcacattgt |
| Mutagenesis codon 837 | cgtttatctgattacNNNgtcgatcacattgtacc |
| Mutagenesis codon 838 | gtttatctgattacgacNNNgatcacattgtaccc |
| Mutagenesis codon 839 | tctgattacgacgtcNNNcacattgtaccc |
| Mutagenesis codon 840 | tacgacgtcgatNNNattgtaccccaatcc |
| Mutagenesis codon 841 | cgacgtcgatcacNNNgtaccccaatcc |
| Mutagenesis codon 842 | cgtcgatcacattNNNccccaatcctttttg |
| Mutagenesis codon 843 | cgacgtcgatcacattgtaNNNcaatcctttttg |
| Mutagenesis codon 844 | cgtcgatcacattgtacccNNNtcctttttgaag |
| Mutagenesis codon 845 | tcacattgtaccccaaNNNtttttgaaggacg |
| Mutagenesis codon 846 | gtaccccaatccNNNttgaaggacgattc |
| Mutagenesis codon 847 | ccccaatcctttNNNaaggacgattcaatcg |
| Mutagenesis codon 848 | cccaatcctttttgNNNgacgattcaatcg |
| Mutagenesis codon 849 | cccaatcctttttgaagNNNgattcaatcgacaa |
| Mutagenesis codon 850 | cccaatcctttttgaaggacNNNtcaatcgacaat |
| Mutagenesis codon 851 | ttgaaggacgatNNNatcgacaataaagtgc |
| Mutagenesis codon 852 | gaaggacgattcaNNNgacaataaagtgcttacac |
| Mutagenesis codon 853 | ggacgattcaatcNNNaataaagtgcttacacgct |
| Mutagenesis codon 854 | ggacgattcaatcgacNNNaaagtgcttacac |
| Mutagenesis codon 855 | tcaatcgacaatNNNgtgcttacacgctcgg |
| Mutagenesis codon 856 | ttcaatcgacaataaaNNNcttacacgctcgga |
| Mutagenesis codon 857 | cgacaataaagtgNNNacacgctcggataag |
| Mutagenesis codon 858 | cgacaataaagtgcttNNNcgctcggataag |
| Mutagenesis codon 859 | cgacaataaagtgcttacaNNNtcggataagaacc |
| Mutagenesis codon 860 | gtgcttacacgcNNNgataagaaccgagg |
| Mutagenesis codon 861 | gcttacacgctcgNNNaagaaccgagggaaa |
| Mutagenesis codon 862 | acacgctcggatNNNaaccgagggaaa |
| Mutagenesis codon 863 | cacgctcggataagNNNcgagggaaaagt |
| Mutagenesis codon 864 | gctcggataagaacNNNgggaaaagtgacaa |
| Mutagenesis codon 865 | cacgctcggataagaaccgaNNNaaaagtgacaat |
| Mutagenesis codon 866 | cggataagaaccgagggNNNagtgacaatgtt |
| Mutagenesis codon 867 | aaccgagggaaaNNNgacaatgttccaagc |
| Mutagenesis codon 868 | cgagggaaaagtNNNaatgttccaagcgagg |
| Mutagenesis codon 869 | gggaaaagtgacNNNgttccaagcgagga |
| Mutagenesis codon 870 | gaaaagtgacaatNNNccaagcgaggaagtc |
| Mutagenesis codon 871 | aagtgacaatgttNNNagcgaggaagtcg |
| Mutagenesis codon 872 | gtgacaatgttccaNNNgaggaagtcgtaaag |
| Mutagenesis codon 873 | gtgacaatgttccaagcNNNgaagtcgtaaag |
| Mutagenesis codon 874 | gacaatgttccaagcgagNNNgtcgtaaagaaa |
| Mutagenesis codon 875 | ccaagcgaggaaNNNgtaaagaaaatgaagaac |
| Mutagenesis codon 876 | ccaagcgaggaagtcNNNaagaaaatgaagaactat |
| Mutagenesis codon 877 | ccaagcgaggaagtcgtaNNNaaaatgaagaac |
| Mutagenesis codon 878 | agcgaggaagtcgtaaagNNNatgaagaactat |
| Mutagenesis codon 879 | gcgaggaagtcgtaaagaaaNNNaagaactattgg |
| Mutagenesis codon 880 | gtcgtaaagaaaatgNNNaactattggcggcag |
| Mutagenesis codon 881 | gtcgtaaagaaaatgaagNNNtattggcggcag |
| Mutagenesis codon 882 | gaaaatgaagaacNNNtggcggcagctcctaaa |
| Mutagenesis codon 883 | aatgaagaactatNNNcggcagctcctaaatgc |
| Mutagenesis codon 884 | aagaactattggNNNcagctcctaaatgcgaaact |
| Mutagenesis codon 885 | aactattggcggNNNctcctaaatgcgaaact |
| Mutagenesis codon 886 | actattggcggcagNNNctaaatgcgaaact |
| Mutagenesis codon 887 | tggcggcagctcNNNaatgcgaaactg |
| Mutagenesis codon 888 | gcggcagctcctaNNNgcgaaactgataa |
| Mutagenesis codon 889 | gcggcagctcctaaatNNNaaactgataacg |
| Mutagenesis codon 890 | gctcctaaatgcgNNNctgataacgcaaaga |
| Mutagenesis codon 891 | gcggcagctcctaaatgcgaaaNNNataacgcaaagaa |
| Mutagenesis codon 892 | cctaaatgcgaaactgNNNacgcaaagaaagt |
| Mutagenesis codon 893 | gctcctaaatgcgaaactgataNNNcaaagaaagttc |
| Mutagenesis codon 894 | gcgaaactgataacgNNNagaaagttcgataac |
| Mutagenesis codon 895 | gcgaaactgataacgcaaNNNaagttcgataac |
| Mutagenesis codon 896 | gcgaaactgataacgcaaagaNNNttcgataacttaac |
| Mutagenesis codon 897 | acgcaaagaaagNNNgataacttaactaaagctgagagg |
| Mutagenesis codon 898 | cgcaaagaaagttcNNNaacttaactaaagctgagag |
| Mutagenesis codon 899 | cgcaaagaaagttcgatNNNttaactaaagctgag |
| Mutagenesis codon 900 | acgcaaagaaagttcgataacNNNactaaagctgag |
| Mutagenesis codon 901 | cgcaaagaaagttcgataacttaNNNaaagctgagagg |
| Mutagenesis codon 902 | cgataacttaactNNNgctgagaggggtggct |
| Mutagenesis codon 903 | gttcgataacttaactaaaNNNgagaggggtggc |
| Mutagenesis codon 904 | aacttaactaaagctNNNaggggtggcttgtc |
| Mutagenesis codon 905 | actaaagctgagNNNggtggcttgtctg |
| Mutagenesis codon 906 | aaagctgagaggNNNggcttgtctgaactt |
| Mutagenesis codon 907 | gctgagaggggtNNNttgtctgaacttg |
| Mutagenesis codon 908 | gagaggggtggcNNNtctgaacttgac |
| Mutagenesis codon 909 | aggggtggcttgNNNgaacttgacaag |
| Mutagenesis codon 910 | ggtggcttgtctNNNcttgacaaggcc |
| Mutagenesis codon 911 | gtggcttgtctgaaNNNgacaaggccggat |
| Mutagenesis codon 912 | ggcttgtctgaacttNNNaaggccggattt |
| Mutagenesis codon 913 | tggcttgtctgaacttgacNNNgccggatttatta |
| Mutagenesis codon 914 | gggtggcttgtctgaacttgacaagNNNggatttattaaa |
| Mutagenesis codon 915 | gtctgaacttgacaaggccNNNtttattaaacgtc |
| Mutagenesis codon 916 | gacaaggccggaNNNattaaacgtcagc |
| Mutagenesis codon 917 | caaggccggatttNNNaaacgtcagctc |
| Mutagenesis codon 918 | gccggatttattNNNcgtcagctcgtgga |
| Mutagenesis codon 919 | ccggatttattaaaNNNcagctcgtggaaacc |
| Mutagenesis codon 920 | ccggatttattaaacgtNNNctcgtggaaacc |
| Mutagenesis codon 921 | ggatttattaaacgtcagNNNgtggaaacccgc |
| Mutagenesis codon 922 | aaacgtcagctcNNNgaaacccgccaaatc |
| Mutagenesis codon 923 | cgtcagctcgtgNNNacccgccaaatc |
| Mutagenesis codon 924 | tcagctcgtggaaNNNcgccaaatcacaaa |
| Mutagenesis codon 925 | gctcgtggaaaccNNNcaaatcacaaagc |
| Mutagenesis codon 926 | cgtggaaacccgcNNNatcacaaagcat |
| Mutagenesis codon 927 | cgtggaaacccgccaaNNNacaaagcatgtt |
| Mutagenesis codon 928 | acccgccaaatcNNNaagcatgttgcacagata |
| Mutagenesis codon 929 | ccgccaaatcacaNNNcatgttgcacaga |
| Mutagenesis codon 930 | ccgccaaatcacaaagNNNgttgcacagata |
| Mutagenesis codon 931 | atcacaaagcatNNNgcacagatactagattccc |
| Mutagenesis codon 932 | ccgccaaatcacaaagcatgttNNNcagatactagat |
| Mutagenesis codon 933 | cacaaagcatgttgcaNNNatactagattcccgaa |
| Mutagenesis codon 934 | gcatgttgcacagNNNctagattcccgaa |
| Mutagenesis codon 935 | gttgcacagataNNNgattcccgaatgaatacg |
| Mutagenesis codon 936 | gttgcacagatactaNNNtcccgaatgaatacg |
| Mutagenesis codon 937 | gcacagatactagatNNNcgaatgaatacg |
| Mutagenesis codon 938 | gcacagatactagattccNNNatgaatacgaaatacg |
| Mutagenesis codon 939 | actagattcccgaNNNaatacgaaatacgacgagaa |
| Mutagenesis codon 940 | gattcccgaatgNNNacgaaatacgacgagaa |
| Mutagenesis codon 941 | tcccgaatgaatNNNaaatacgacgagaacg |
| Mutagenesis codon 942 | cccgaatgaatacgNNNtacgacgagaac |
| Mutagenesis codon 943 | ccgaatgaatacgaaaNNNgacgagaacgataag |
| Mutagenesis codon 944 | ccgaatgaatacgaaatacNNNgagaacgataagc |
| Mutagenesis codon 945 | acgaaatacgacNNNaacgataagctgattc |
| Mutagenesis codon 946 | gaaatacgacgagNNNgataagctgattcgggaagt |
| Mutagenesis codon 947 | tacgacgagaacNNNaagctgattcgggaa |
| Mutagenesis codon 948 | cgacgagaacgatNNNctgattcgggaa |
| Mutagenesis codon 949 | gacgagaacgataagNNNattcgggaagtc |
| Mutagenesis codon 950 | aacgataagctgNNNcgggaagtcaaagta |
| Mutagenesis codon 951 | gacgagaacgataagctgattNNNgaagtcaaagta |
| Mutagenesis codon 952 | gaacgataagctgattcggNNNgtcaaagtaatc |
| Mutagenesis codon 953 | cgagaacgataagctgattcgggaaNNNaaagtaatcact |
| Mutagenesis codon 954 | gctgattcgggaagtcNNNgtaatcactttaaa |
| Mutagenesis codon 955 | gctgattcgggaagtcaaaNNNatcactttaaag |
| Mutagenesis codon 956 | gctgattcgggaagtcaaagtaNNNactttaaagtca |
| Mutagenesis codon 957 | cgggaagtcaaagtaatcNNNttaaagtcaaaattgg |
| Mutagenesis codon 958 | cgggaagtcaaagtaatcactNNNaagtcaaaattg |
| Mutagenesis codon 959 | gtaatcactttaNNNtcaaaattggtgtcggacttcag |
| Mutagenesis codon 960 | atcactttaaagNNNaaattggtgtcggacttcag |
| Mutagenesis codon 961 | tcactttaaagtcaNNNttggtgtcggacttcag |
| Mutagenesis codon 962 | ttaaagtcaaaaNNNgtgtcggacttcagaaagg |
| Mutagenesis codon 963 | aagtcaaaattgNNNtcggacttcagaaagg |
| Mutagenesis codon 964 | gtcaaaattggtgNNNgacttcagaaaggat |
| Mutagenesis codon 965 | gtcaaaattggtgtcgNNNttcagaaaggat |
| Mutagenesis codon 966 | ttggtgtcggacNNNagaaaggattttcaattc |
| Mutagenesis codon 967 | tggtgtcggacttcNNNaaggattttcaattc |
| Mutagenesis codon 968 | ggtgtcggacttcagaNNNgattttcaattc |
| Mutagenesis codon 969 | ggtgtcggacttcagaaagNNNtttcaattctat |
| Mutagenesis codon 970 | gtcggacttcagaaaggatNNNcaattctataaag |
| Mutagenesis codon 971 | gtcggacttcagaaaggattttNNNttctataaagttagg |
| Mutagenesis codon 972 | cggacttcagaaaggattttcaaNNNtataaagttaggg |
| Mutagenesis codon 973 | ggacttcagaaaggattttcaattcNNNaaagttagggag |
| Mutagenesis codon 974 | cttcagaaaggattttcaattctatNNNgttagggagata |
| Mutagenesis codon 975 | caattctataaaNNNagggagataaataactaccaccatg |
| Mutagenesis codon 976 | ttctataaagttNNNgagataaataactaccaccatgcgc |
| Mutagenesis codon 977 | ctataaagttaggNNNataaataactaccaccatgcgc |
| Mutagenesis codon 978 | aaagttagggagNNNaataactaccaccatgc |
| Mutagenesis codon 979 | gttagggagataNNNaactaccaccatgcg |
| Mutagenesis codon 980 | agggagataaatNNNtaccaccatgcgcac |
| Mutagenesis codon 981 | gagataaataacNNNcaccatgcgcacgac |
| Mutagenesis codon 982 | gggagataaataactacNNNcatgcgcacgac |
| Mutagenesis codon 983 | gagataaataactaccacNNNgcgcacgacgctta |
| Mutagenesis codon 984 | taactaccaccatNNNcacgacgcttatct |
| Mutagenesis codon 985 | taccaccatgcgNNNgacgcttatcttaatg |
| Mutagenesis codon 986 | ccaccatgcgcacNNNgcttatcttaatg |
| Mutagenesis codon 987 | ccatgcgcacgacNNNtatcttaatgcc |
| Mutagenesis codon 988 | tgcgcacgacgctNNNcttaatgccgtc |
| Mutagenesis codon 989 | gcacgacgcttatNNNaatgccgtcgta |
| Mutagenesis codon 990 | gacgcttatcttNNNgccgtcgtaggg |
| Mutagenesis codon 991 | gacgcttatcttaatNNNgtcgtagggacc |
| Mutagenesis codon 992 | tatcttaatgccNNNgtagggaccgcac |
| Mutagenesis codon 993 | atcttaatgccgtcNNNgggaccgcactc |
| Mutagenesis codon 994 | aatgccgtcgtaNNNaccgcactcatta |
| Mutagenesis codon 995 | gccgtcgtagggNNNgcactcattaagaaata |
| Mutagenesis codon 996 | ccgtcgtagggaccNNNctcattaagaaata |
| Mutagenesis codon 997 | cgtagggaccgcaNNNattaagaaataccc |
| Mutagenesis codon 998 | gggaccgcactcNNNaagaaatacccgaa |
| Mutagenesis codon 999 | gaccgcactcattNNNaaatacccgaagc |
| Mutagenesis codon 1000 | cgcactcattaagNNNtacccgaagctag |
| Mutagenesis codon 1001 | ccgcactcattaagaaaNNNccgaagctagaa |
| Mutagenesis codon 1002 | ggaccgcactcattaagaaatacNNNaagctagaaagt |
| Mutagenesis codon 1003 | ctcattaagaaatacccgNNNctagaaagtgag |
| Mutagenesis codon 1004 | agaaatacccgaagNNNgaaagtgagtttgtgtatg |
| Mutagenesis codon 1005 | tacccgaagctaNNNagtgagtttgtgtatgg |
| Mutagenesis codon 1006 | acccgaagctagaaNNNgagtttgtgtatgg |
| Mutagenesis codon 1007 | cccgaagctagaaagtNNNtttgtgtatggtg |
| Mutagenesis codon 1008 | gaagctagaaagtgagNNNgtgtatggtgatta |
| Mutagenesis codon 1009 | cccgaagctagaaagtgagtttNNNtatggtgattac |
| Mutagenesis codon 1010 | gctagaaagtgagtttgtgNNNggtgattacaaagt |
| Mutagenesis codon 1011 | gagtttgtgtatNNNgattacaaagtttatgacgtccg |
| Mutagenesis codon 1012 | gtttgtgtatggtNNNtacaaagtttatgacgtccg |
| Mutagenesis codon 1013 | gtgtatggtgatNNNaaagtttatgacgtccgt |
| Mutagenesis codon 1014 | tgtgtatggtgattacNNNgtttatgacgtccg |
| Mutagenesis codon 1015 | ggtgattacaaaNNNtatgacgtccgtaagatg |
| Mutagenesis codon 1016 | gtatggtgattacaaagttNNNgacgtccgtaag |
| Mutagenesis codon 1017 | ggtgattacaaagtttatNNNgtccgtaagatgatcgc |
| Mutagenesis codon 1018 | acaaagtttatgacNNNcgtaagatgatcgc |
| Mutagenesis codon 1019 | caaagtttatgacgtcNNNaagatgatcgcgaaa |
| Mutagenesis codon 1020 | tatgacgtccgtNNNatgatcgcgaaaagc |
| Mutagenesis codon 1021 | gacgtccgtaagNNNatcgcgaaaagc |
| Mutagenesis codon 1022 | gtccgtaagatgNNNgcgaaaagcgaacag |
| Mutagenesis codon 1023 | ccgtaagatgatcNNNaaaagcgaacaggagata |
| Mutagenesis codon 1024 | aagatgatcgcgNNNagcgaacaggagata |
| Mutagenesis codon 1025 | gatgatcgcgaaaNNNgaacaggagatagg |
| Mutagenesis codon 1026 | atcgcgaaaagcNNNcaggagataggc |
| Mutagenesis codon 1027 | gcgaaaagcgaaNNNgagataggcaagg |
| Mutagenesis codon 1028 | cgcgaaaagcgaacagNNNataggcaaggcta |
| Mutagenesis codon 1029 | agcgaacaggagNNNggcaaggctaca |
| Mutagenesis codon 1030 | agcgaacaggagataNNNaaggctacagccaaata |
| Mutagenesis codon 1031 | caggagataggcNNNgctacagccaaata |
| Mutagenesis codon 1032 | acaggagataggcaagNNNacagccaaatac |
| Mutagenesis codon 1033 | gagataggcaaggctNNNgccaaatacttctt |
| Mutagenesis codon 1034 | cgaacaggagataggcaaggctacaNNNaaatacttcttt |
| Mutagenesis codon 1035 | ggcaaggctacagccNNNtacttcttttattc |
| Mutagenesis codon 1036 | ggcaaggctacagccaaaNNNttcttttattctaac |
| Mutagenesis codon 1037 | ggcaaggctacagccaaatacNNNttttattctaac |
| Mutagenesis codon 1038 | ggcaaggctacagccaaatacttcNNNtattctaacatt |
| Mutagenesis codon 1039 | ggctacagccaaatacttctttNNNtctaacattatg |
| Mutagenesis codon 1040 | ggctacagccaaatacttcttttatNNNaacattatgaat |
| Mutagenesis codon 1041 | cagccaaatacttcttttattctNNNattatgaatttc |
| Mutagenesis codon 1042 | gccaaatacttcttttattctaacNNNatgaatttcttt |
| Mutagenesis codon 1043 | tattctaacattNNNaatttctttaagacggaaatcactc |
| Mutagenesis codon 1044 | tctaacattatgNNNttctttaagacggaaatcactctgg |
| Mutagenesis codon 1045 | aacattatgaatNNNtttaagacggaaatcactctggc |
| Mutagenesis codon 1046 | cattatgaatttcNNNaagacggaaatcactctggcaaac |
| Mutagenesis codon 1047 | atgaatttctttNNNacggaaatcactctggcaaacgg |
| Mutagenesis codon 1048 | gaatttctttaagNNNgaaatcactctggcaaacgg |
| Mutagenesis codon 1049 | ttctttaagacgNNNatcactctggcaaacggaga |
| Mutagenesis codon 1050 | tttaagacggaaNNNactctggcaaacggaga |
| Mutagenesis codon 1051 | ctttaagacggaaatcNNNctggcaaacgga |
| Mutagenesis codon 1052 | gacggaaatcactNNNgcaaacggagag |
| Mutagenesis codon 1053 | gacggaaatcactctgNNNaacggagagata |
| Mutagenesis codon 1054 | atcactctggcaNNNggagagatacgc |
| Mutagenesis codon 1055 | cactctggcaaacNNNgagatacgcaaac |
| Mutagenesis codon 1056 | ctggcaaacggaNNNatacgcaaacgac |
| Mutagenesis codon 1057 | gcaaacggagagNNNcgcaaacgaccttta |
| Mutagenesis codon 1058 | ctggcaaacggagagataNNNaaacgaccttta |
| Mutagenesis codon 1059 | cggagagatacgcNNNcgacctttaattg |
| Mutagenesis codon 1060 | cggagagatacgcaaaNNNcctttaattgaaacc |
| Mutagenesis codon 1061 | cggagagatacgcaaacgaNNNttaattgaaacc |
| Mutagenesis codon 1062 | acgcaaacgacctNNNattgaaaccaatggg |
| Mutagenesis codon 1063 | aaacgacctttaNNNgaaaccaatggggagaca |
| Mutagenesis codon 1064 | cgacctttaattNNNaccaatggggagacag |
| Mutagenesis codon 1065 | acgcaaacgacctttaattgaaNNNaatggggagaca |
| Mutagenesis codon 1066 | tttaattgaaaccNNNggggagacaggtgaaatc |
| Mutagenesis codon 1067 | cgacctttaattgaaaccaatNNNgagacaggtgaa |
| Mutagenesis codon 1068 | gaaaccaatgggNNNacaggtgaaatcgtatg |
| Mutagenesis codon 1069 | accaatggggagNNNggtgaaatcgtatgggataag |
| Mutagenesis codon 1070 | aatggggagacaNNNgaaatcgtatgggataag |
| Mutagenesis codon 1071 | ggggagacaggtNNNatcgtatgggat |
| Mutagenesis codon 1072 | gggagacaggtgaaNNNgtatgggataag |
| Mutagenesis codon 1073 | gacaggtgaaatcNNNtgggataagggc |
| Mutagenesis codon 1074 | ggtgaaatcgtaNNNgataagggccgg |
| Mutagenesis codon 1075 | gaaatcgtatggNNNaagggccgggactt |
| Mutagenesis codon 1076 | atcgtatgggatNNNggccgggacttc |
| Mutagenesis codon 1077 | atcgtatgggataagNNNcgggacttcgcg |
| Mutagenesis codon 1078 | tgggataagggcNNNgacttcgcgacg |
| Mutagenesis codon 1079 | tgggataagggccggNNNttcgcgacggtgaga |
| Mutagenesis codon 1080 | aagggccgggacNNNgcgacggtgagaaa |
| Mutagenesis codon 1081 | ggccgggacttcNNNacggtgagaaaa |
| Mutagenesis codon 1082 | cgggacttcgcgNNNgtgagaaaagtt |
| Mutagenesis codon 1083 | cgggacttcgcgacgNNNagaaaagttttg |
| Mutagenesis codon 1084 | ttcgcgacggtgNNNaaagttttgtccatg |
| Mutagenesis codon 1085 | gcgacggtgagaNNNgttttgtccatg |
| Mutagenesis codon 1086 | gcgacggtgagaaaaNNNttgtccatgccc |
| Mutagenesis codon 1087 | cggtgagaaaagttNNNtccatgccccaag |
| Mutagenesis codon 1088 | cggtgagaaaagttttgNNNatgccccaagtc |
| Mutagenesis codon 1089 | aaagttttgtccNNNccccaagtcaacatagta |
| Mutagenesis codon 1090 | cggtgagaaaagttttgtccatgNNNcaagtcaacatagt |
| Mutagenesis codon 1091 | ttgtccatgcccNNNgtcaacatagtaaagaaa |
| Mutagenesis codon 1092 | gtccatgccccaaNNNaacatagtaaagaaaac |
| Mutagenesis codon 1093 | gtccatgccccaagtcNNNatagtaaagaaa |
| Mutagenesis codon 1094 | gccccaagtcaacNNNgtaaagaaaactgag |
| Mutagenesis codon 1095 | gccccaagtcaacataNNNaagaaaactgagg |
| Mutagenesis codon 1096 | gccccaagtcaacatagtaNNNaaaactgaggtg |
| Mutagenesis codon 1097 | cccaagtcaacatagtaaagNNNactgaggtgcaga |
| Mutagenesis codon 1098 | atagtaaagaaaNNNgaggtgcagaccggagg |
| Mutagenesis codon 1099 | gtaaagaaaactNNNgtgcagaccggagggtt |
| Mutagenesis codon 1100 | aaagaaaactgagNNNcagaccggaggg |
| Mutagenesis codon 1101 | aaagaaaactgaggtgNNNaccggagggttt |
| Mutagenesis codon 1102 | actgaggtgcagNNNggagggttttca |
| Mutagenesis codon 1103 | tgaggtgcagaccNNNgggttttcaaag |
| Mutagenesis codon 1104 | ggtgcagaccggaNNNttttcaaaggaa |
| Mutagenesis codon 1105 | gcagaccggagggNNNtcaaaggaatcg |
| Mutagenesis codon 1106 | gaccggagggtttNNNaaggaatcgattc |
| Mutagenesis codon 1107 | gaccggagggttttcaNNNgaatcgattctt |
| Mutagenesis codon 1108 | cggagggttttcaaagNNNtcgattcttccaaa |
| Mutagenesis codon 1109 | ccggagggttttcaaaggaaNNNattcttccaaaaa |
| Mutagenesis codon 1110 | tcaaaggaatcgNNNcttccaaaaaggaatagtg |
| Mutagenesis codon 1111 | caaaggaatcgattNNNccaaaaaggaatagtg |
| Mutagenesis codon 1112 | gggttttcaaaggaatcgattcttNNNaaaaggaatagtg |
| Mutagenesis codon 1113 | tcgattcttccaNNNaggaatagtgataagctc |
| Mutagenesis codon 1114 | attcttccaaaaNNNaatagtgataagctcatcgctcg |
| Mutagenesis codon 1115 | cttccaaaaaggNNNagtgataagctcatcg |
| Mutagenesis codon 1116 | ccaaaaaggaatNNNgataagctcatcgctcg |
| Mutagenesis codon 1117 | ccaaaaaggaatagtNNNaagctcatcgctcg |
| Mutagenesis codon 1118 | aaaggaatagtgatNNNctcatcgctcgt |
| Mutagenesis codon 1119 | aggaatagtgataagNNNatcgctcgtaaaaagg |
| Mutagenesis codon 1120 | ggaatagtgataagctcNNNgctcgtaaaaag |
| Mutagenesis codon 1121 | agtgataagctcatcNNNcgtaaaaaggactg |
| Mutagenesis codon 1122 | aagctcatcgctNNNaaaaaggactgggac |
| Mutagenesis codon 1123 | gctcatcgctcgtNNNaaggactgggac |
| Mutagenesis codon 1124 | atcgctcgtaaaNNNgactgggacccg |
| Mutagenesis codon 1125 | cgctcgtaaaaagNNNtgggacccgaaa |
| Mutagenesis codon 1126 | gctcgtaaaaaggacNNNgacccgaaaaagta |
| Mutagenesis codon 1127 | ctcgtaaaaaggactggNNNccgaaaaagtac |
| Mutagenesis codon 1128 | aaggactgggacNNNaaaaagtacggtgg |
| Mutagenesis codon 1129 | gactgggacccgNNNaagtacggtggctt |
| Mutagenesis codon 1130 | ctgggacccgaaaNNNtacggtggcttc |
| Mutagenesis codon 1131 | gacccgaaaaagNNNggtggcttcgatag |
| Mutagenesis codon 1132 | ccgaaaaagtacNNNggcttcgatagcc |
| Mutagenesis codon 1133 | cgaaaaagtacggtNNNttcgatagccctac |
| Mutagenesis codon 1134 | aagtacggtggcNNNgatagccctaca |
| Mutagenesis codon 1135 | tacggtggcttcNNNagccctacagtt |
| Mutagenesis codon 1136 | cggtggcttcgatNNNcctacagttgcctatt |
| Mutagenesis codon 1137 | gtggcttcgatagcNNNacagttgcctat |
| Mutagenesis codon 1138 | gcttcgatagccctNNNgttgcctattct |
| Mutagenesis codon 1139 | gatagccctacaNNNgcctattctgtcc |
| Mutagenesis codon 1140 | cgatagccctacagttNNNtattctgtcctag |
| Mutagenesis codon 1141 | gccctacagttgccNNNtctgtcctagta |
| Mutagenesis codon 1142 | acagttgcctatNNNgtcctagtagtggc |
| Mutagenesis codon 1143 | cagttgcctattctNNNctagtagtggca |
| Mutagenesis codon 1144 | gttgcctattctgtcNNNgtagtggcaaaag |
| Mutagenesis codon 1145 | gttgcctattctgtcctaNNNgtggcaaaagtt |
| Mutagenesis codon 1146 | tctgtcctagtaNNNgcaaaagttgagaagggaaa |
| Mutagenesis codon 1147 | gtcctagtagtgNNNaaagttgagaagggaaa |
| Mutagenesis codon 1148 | cctagtagtggcaNNNgttgagaagggaaa |
| Mutagenesis codon 1149 | agtagtggcaaaaNNNgagaagggaaaatcc |
| Mutagenesis codon 1150 | gtcctagtagtggcaaaagttNNNaagggaaaatcc |
| Mutagenesis codon 1151 | gtggcaaaagttgagNNNggaaaatccaagaaa |
| Mutagenesis codon 1152 | ggcaaaagttgagaagNNNaaatccaagaaactg |
| Mutagenesis codon 1153 | gttgagaagggaNNNtccaagaaactgaag |
| Mutagenesis codon 1154 | gagaagggaaaaNNNaagaaactgaagtcagtca |
| Mutagenesis codon 1155 | gagaagggaaaatccNNNaaactgaagtcagtca |
| Mutagenesis codon 1156 | agggaaaatccaagNNNctgaagtcagtcaaaga |
| Mutagenesis codon 1157 | gagaagggaaaatccaagaaaNNNaagtcagtcaaag |
| Mutagenesis codon 1158 | gggaaaatccaagaaactgNNNtcagtcaaagaa |
| Mutagenesis codon 1159 | ccaagaaactgaagNNNgtcaaagaattattggg |
| Mutagenesis codon 1160 | gggaaaatccaagaaactgaagtcaNNNaaagaattattg |
| Mutagenesis codon 1161 | gaaactgaagtcagtcNNNgaattattggggataac |
| Mutagenesis codon 1162 | ctgaagtcagtcaaaNNNttattggggataacg |
| Mutagenesis codon 1163 | gtcagtcaaagaaNNNttggggataacgattatg |
| Mutagenesis codon 1164 | agtcaaagaattaNNNgggataacgattatggagcg |
| Mutagenesis codon 1165 | caaagaattattgNNNataacgattatggagcgctcg |
| Mutagenesis codon 1166 | cagtcaaagaattattggggNNNacgattatggag |
| Mutagenesis codon 1167 | caaagaattattggggataNNNattatggagcgctc |
| Mutagenesis codon 1168 | ttggggataacgNNNatggagcgctcg |
| Mutagenesis codon 1169 | gggataacgattNNNgagcgctcgtct |
| Mutagenesis codon 1170 | gggataacgattatgNNNcgctcgtcttttg |
| Mutagenesis codon 1171 | tggggataacgattatggagNNNtcgtcttttgaa |
| Mutagenesis codon 1172 | cgattatggagcgcNNNtcttttgaaaagaacc |
| Mutagenesis codon 1173 | cgattatggagcgctcgNNNtttgaaaagaac |
| Mutagenesis codon 1174 | gagcgctcgtctNNNgaaaagaacccc |
| Mutagenesis codon 1175 | gcgctcgtcttttNNNaagaaccccatc |
| Mutagenesis codon 1176 | gctcgtcttttgaaNNNaaccccatcgac |
| Mutagenesis codon 1177 | ctcgtcttttgaaaagNNNcccatcgacttc |
| Mutagenesis codon 1178 | gcgctcgtcttttgaaaagaacNNNatcgacttcctt |
| Mutagenesis codon 1179 | tgaaaagaaccccNNNgacttccttgagg |
| Mutagenesis codon 1180 | aagaaccccatcNNNttccttgaggcgaa |
| Mutagenesis codon 1181 | aaccccatcgacNNNcttgaggcgaaag |
| Mutagenesis codon 1182 | cccatcgacttcNNNgaggcgaaaggttaca |
| Mutagenesis codon 1183 | ccatcgacttccttNNNgcgaaaggttac |
| Mutagenesis codon 1184 | cgacttccttgagNNNaaaggttacaaggaagta |
| Mutagenesis codon 1185 | ttccttgaggcgNNNggttacaaggaagta |
| Mutagenesis codon 1186 | cttccttgaggcgaaaNNNtacaaggaagta |
| Mutagenesis codon 1187 | gaggcgaaaggtNNNaaggaagtaaaaaaggatct |
| Mutagenesis codon 1188 | ggcgaaaggttacNNNgaagtaaaaaaggatct |
| Mutagenesis codon 1189 | ggcgaaaggttacaagNNNgtaaaaaaggatct |
| Mutagenesis codon 1190 | ggcgaaaggttacaaggaaNNNaaaaaggatctc |
| Mutagenesis codon 1191 | ggcgaaaggttacaaggaagtaNNNaaggatctcataa |
| Mutagenesis codon 1192 | ggcgaaaggttacaaggaagtaaaaNNNgatctcataatt |
| Mutagenesis codon 1193 | caaggaagtaaaaaagNNNctcataattaaactaccaaag |
| Mutagenesis codon 1194 | caaggaagtaaaaaaggatNNNataattaaactaccaaag |
| Mutagenesis codon 1195 | ggaagtaaaaaaggatctcNNNattaaactaccaaag |
| Mutagenesis codon 1196 | caaggaagtaaaaaaggatctcataNNNaaactaccaaag |
| Mutagenesis codon 1197 | ggatctcataattNNNctaccaaagtatagtctg |
| Mutagenesis codon 1198 | ggatctcataattaaaNNNccaaagtatagtctg |
| Mutagenesis codon 1199 | ggatctcataattaaactaNNNaagtatagtctgtttgag |
| Mutagenesis codon 1200 | ggatctcataattaaactaccaNNNtatagtctgtttgag |
| Mutagenesis codon 1201 | aaactaccaaagNNNagtctgtttgagttagaaaatgg |
| Mutagenesis codon 1202 | ctaccaaagtatNNNctgtttgagttagaaaatggc |
| Mutagenesis codon 1203 | ccaaagtatagtNNNtttgagttagaaaatggccg |
| Mutagenesis codon 1204 | ccaaagtatagtctgNNNgagttagaaaatggc |
| Mutagenesis codon 1205 | gtatagtctgtttNNNttagaaaatggccg |
| Mutagenesis codon 1206 | aaagtatagtctgtttgagNNNgaaaatggccga |
| Mutagenesis codon 1207 | tctgtttgagttaNNNaatggccgaaaacggatg |
| Mutagenesis codon 1208 | tttgagttagaaNNNggccgaaaacggatg |
| Mutagenesis codon 1209 | tgagttagaaaatNNNcgaaaacggatgttggc |
| Mutagenesis codon 1210 | gagttagaaaatggcNNNaaacggatgttg |
| Mutagenesis codon 1211 | agaaaatggccgaNNNcggatgttggct |
| Mutagenesis codon 1212 | aatggccgaaaaNNNatgttggctagcgccggagag |
| Mutagenesis codon 1213 | ggccgaaaacggNNNttggctagcgccggaga |
| Mutagenesis codon 1214 | gccgaaaacggatgNNNgctagcgccggagag |
| Mutagenesis codon 1215 | aaacggatgttgNNNagcgccggagag |
| Mutagenesis codon 1216 | acggatgttggctNNNgccggagagcttca |
| Mutagenesis codon 1217 | ggatgttggctagcNNNggagagcttcaa |
| Mutagenesis codon 1218 | ttggctagcgccNNNgagcttcaaaag |
| Mutagenesis codon 1219 | gctagcgccggaNNNcttcaaaagggg |
| Mutagenesis codon 1220 | agcgccggagagNNNcaaaaggggaac |
| Mutagenesis codon 1221 | cgccggagagcttNNNaaggggaacgaact |
| Mutagenesis codon 1222 | ggagagcttcaaNNNgggaacgaactc |
| Mutagenesis codon 1223 | gccggagagcttcaaaagNNNaacgaactcgcacta |
| Mutagenesis codon 1224 | gcttcaaaaggggNNNgaactcgcacta |
| Mutagenesis codon 1225 | cttcaaaaggggaacNNNctcgcactaccgtctaaat |
| Mutagenesis codon 1226 | aaggggaacgaaNNNgcactaccgtcta |
| Mutagenesis codon 1227 | gggaacgaactcNNNctaccgtctaaatac |
| Mutagenesis codon 1228 | gaacgaactcgcaNNNccgtctaaatacg |
| Mutagenesis codon 1229 | gggaacgaactcgcactaNNNtctaaatacgtgaa |
| Mutagenesis codon 1230 | cgaactcgcactaccgNNNaaatacgtgaat |
| Mutagenesis codon 1231 | ctcgcactaccgtctNNNtacgtgaatttc |
| Mutagenesis codon 1232 | cgcactaccgtctaaaNNNgtgaatttcctg |
| Mutagenesis codon 1233 | cgcactaccgtctaaatacNNNaatttcctgtat |
| Mutagenesis codon 1234 | ccgtctaaatacgtgNNNttcctgtatttagc |
| Mutagenesis codon 1235 | ccgtctaaatacgtgaatNNNctgtatttagcgtc |
| Mutagenesis codon 1236 | ccgtctaaatacgtgaatttcNNNtatttagcgtccca |
| Mutagenesis codon 1237 | cgtgaatttcctgNNNttagcgtcccattac |
| Mutagenesis codon 1238 | cgtgaatttcctgtatNNNgcgtcccattac |
| Mutagenesis codon 1239 | cgtgaatttcctgtatttaNNNtcccattacgag |
| Mutagenesis codon 1240 | cctgtatttagcgNNNcattacgagaagttgaaag |
| Mutagenesis codon 1241 | cctgtatttagcgtccNNNtacgagaagttgaa |
| Mutagenesis codon 1242 | ttagcgtcccatNNNgagaagttgaaaggttc |
| Mutagenesis codon 1243 | agcgtcccattacNNNaagttgaaaggttc |
| Mutagenesis codon 1244 | gcgtcccattacgagNNNttgaaaggttca |
| Mutagenesis codon 1245 | cccattacgagaagNNNaaaggttcacctga |
| Mutagenesis codon 1246 | tacgagaagttgNNNggttcacctgaagataac |
| Mutagenesis codon 1247 | cgtcccattacgagaagttgaaaNNNtcacctgaagat |
| Mutagenesis codon 1248 | cgagaagttgaaaggtNNNcctgaagataacg |
| Mutagenesis codon 1249 | gagaagttgaaaggttcaNNNgaagataacgaacag |
| Mutagenesis codon 1250 | gaaaggttcacctNNNgataacgaacagaagc |
| Mutagenesis codon 1251 | ggttcacctgaaNNNaacgaacagaagcaac |
| Mutagenesis codon 1252 | ggttcacctgaagatNNNgaacagaagcaactt |
| Mutagenesis codon 1253 | tcacctgaagataacNNNcagaagcaacttt |
| Mutagenesis codon 1254 | gaagataacgaaNNNaagcaactttttgttgagcagcac |
| Mutagenesis codon 1255 | cacctgaagataacgaacagNNNcaactttttgttgag |
| Mutagenesis codon 1256 | cctgaagataacgaacagaagNNNctttttgttgag |
| Mutagenesis codon 1257 | cgaacagaagcaaNNNtttgttgagcagcac |
| Mutagenesis codon 1258 | gaacagaagcaacttNNNgttgagcagcac |
| Mutagenesis codon 1259 | gaagcaactttttNNNgagcagcacaaacattatc |
| Mutagenesis codon 1260 | gcaactttttgttNNNcagcacaaacattatctcg |
| Mutagenesis codon 1261 | gcaactttttgttgagNNNcacaaacattatctcg |
| Mutagenesis codon 1262 | tttgttgagcagNNNaaacattatctcgacgaaatc |
| Mutagenesis codon 1263 | tgttgagcagcacNNNcattatctcgacgaa |
| Mutagenesis codon 1264 | gttgagcagcacaaaNNNtatctcgacgaaa |
| Mutagenesis codon 1265 | gagcagcacaaacatNNNctcgacgaaatc |
| Mutagenesis codon 1266 | gcagcacaaacattatNNNgacgaaatcatagagc |
| Mutagenesis codon 1267 | gagcagcacaaacattatctcNNNgaaatcatagag |
| Mutagenesis codon 1268 | gcagcacaaacattatctcgacNNNatcatagagcaaat |
| Mutagenesis codon 1269 | gcagcacaaacattatctcgacgaaNNNatagagcaaatt |
| Mutagenesis codon 1270 | gcacaaacattatctcgacgaaatcNNNgagcaaatttcg |
| Mutagenesis codon 1271 | ctcgacgaaatcataNNNcaaatttcggaattcag |
| Mutagenesis codon 1272 | ctcgacgaaatcatagagNNNatttcggaattc |
| Mutagenesis codon 1273 | cgacgaaatcatagagcaaNNNtcggaattcagt |
| Mutagenesis codon 1274 | atagagcaaattNNNgaattcagtaagagagtcatcc |
| Mutagenesis codon 1275 | gagcaaatttcgNNNttcagtaagagagtcatc |
| Mutagenesis codon 1276 | gagcaaatttcggaaNNNagtaagagagtcatcc |
| Mutagenesis codon 1277 | gagcaaatttcggaattcNNNaagagagtcatcc |
| Mutagenesis codon 1278 | tcggaattcagtNNNagagtcatcctagctg |
| Mutagenesis codon 1279 | tcggaattcagtaagNNNgtcatcctagctg |
| Mutagenesis codon 1280 | ttcagtaagagaNNNatcctagctgatgccaatc |
| Mutagenesis codon 1281 | cagtaagagagtcNNNctagctgatgccaatct |
| Mutagenesis codon 1282 | gtaagagagtcatcNNNgctgatgccaatct |
| Mutagenesis codon 1283 | gagagtcatcctaNNNgatgccaatctggacaa |
| Mutagenesis codon 1284 | gagtcatcctagctNNNgccaatctggac |
| Mutagenesis codon 1285 | gagtcatcctagctgatNNNaatctggacaaagt |
| Mutagenesis codon 1286 | atcctagctgatgccNNNctggacaaagtatta |
| Mutagenesis codon 1287 | gctgatgccaatNNNgacaaagtattaagcgc |
| Mutagenesis codon 1288 | gctgatgccaatctgNNNaaagtattaagcg |
| Mutagenesis codon 1289 | tgccaatctggacNNNgtattaagcgcataca |
| Mutagenesis codon 1290 | tgccaatctggacaaaNNNttaagcgcataca |
| Mutagenesis codon 1291 | ctggacaaagtaNNNagcgcatacaacaagcacag |
| Mutagenesis codon 1292 | ggacaaagtattaNNNgcatacaacaagcacag |
| Mutagenesis codon 1293 | ggacaaagtattaagcNNNtacaacaagcacagg |
| Mutagenesis codon 1294 | ggacaaagtattaagcgcaNNNaacaagcacagggataa |
| Mutagenesis codon 1295 | ggacaaagtattaagcgcatacNNNaagcacagggataa |
| Mutagenesis codon 1296 | agcgcatacaacNNNcacagggataaacccatac |
| Mutagenesis codon 1297 | gcgcatacaacaagNNNagggataaacccata |
| Mutagenesis codon 1298 | gcgcatacaacaagcacNNNgataaacccatac |
| Mutagenesis codon 1299 | aacaagcacaggNNNaaacccatacgtgag |
| Mutagenesis codon 1300 | caagcacagggatNNNcccatacgtgag |
| Mutagenesis codon 1301 | gcacagggataaaNNNatacgtgagcaggcggaaa |
| Mutagenesis codon 1302 | cagggataaacccNNNcgtgagcaggcg |
| Mutagenesis codon 1303 | agggataaacccataNNNgagcaggcggaaa |
| Mutagenesis codon 1304 | ggataaacccatacgtNNNcaggcggaaaat |
| Mutagenesis codon 1305 | agggataaacccatacgtgagNNNgcggaaaatatta |
| Mutagenesis codon 1306 | cccatacgtgagcagNNNgaaaatattatcc |
| Mutagenesis codon 1307 | cccatacgtgagcaggcgNNNaatattatccatt |
| Mutagenesis codon 1308 | cgtgagcaggcggaaNNNattatccatttg |
| Mutagenesis codon 1309 | cgtgagcaggcggaaaatNNNatccatttgttt |
| Mutagenesis codon 1310 | gcaggcggaaaatattNNNcatttgtttactcttacc |
| Mutagenesis codon 1311 | gagcaggcggaaaatattatcNNNttgtttactcttacc |
| Mutagenesis codon 1312 | aatattatccatNNNtttactcttaccaacctcggcgctc |
| Mutagenesis codon 1313 | ggcggaaaatattatccatttgNNNactcttaccaac |
| Mutagenesis codon 1314 | atccatttgtttNNNcttaccaacctcggc |
| Mutagenesis codon 1315 | ccatttgtttactNNNaccaacctcggcgctcc |
| Mutagenesis codon 1316 | ttgtttactcttNNNaacctcggcgctcc |
| Mutagenesis codon 1317 | tttactcttaccNNNctcggcgctcca |
| Mutagenesis codon 1318 | actcttaccaacNNNggcgctccagcc |
| Mutagenesis codon 1319 | cttaccaacctcNNNgctccagccgcattca |
| Mutagenesis codon 1320 | accaacctcggcNNNccagccgcattca |
| Mutagenesis codon 1321 | aacctcggcgctNNNgccgcattcaag |
| Mutagenesis codon 1322 | ctcggcgctccaNNNgcattcaagtat |
| Mutagenesis codon 1323 | cggcgctccagccNNNttcaagtatttt |
| Mutagenesis codon 1324 | gctccagccgcaNNNaagtattttgacacaac |
| Mutagenesis codon 1325 | gcgctccagccgcattcNNNtattttgacaca |
| Mutagenesis codon 1326 | gccgcattcaagNNNtttgacacaacgataga |
| Mutagenesis codon 1327 | gccgcattcaagtatNNNgacacaacgatag |
| Mutagenesis codon 1328 | ccagccgcattcaagtattttNNNacaacgatagat |
| Mutagenesis codon 1329 | ccgcattcaagtattttgacNNNacgatagatcgc |
| Mutagenesis codon 1330 | gccgcattcaagtattttgacacaNNNatagatcgcaaac |
| Mutagenesis codon 1331 | caagtattttgacacaacgNNNgatcgcaaacga |
| Mutagenesis codon 1332 | ttgacacaacgataNNNcgcaaacgatacac |
| Mutagenesis codon 1333 | gacacaacgatagatNNNaaacgatacacttc |
| Mutagenesis codon 1334 | acgatagatcgcNNNcgatacacttctacca |
| Mutagenesis codon 1335 | caacgatagatcgcaaaNNNtacacttctaccaag |
| Mutagenesis codon 1336 | gatcgcaaacgaNNNacttctaccaaggag |
| Mutagenesis codon 1337 | tcgcaaacgatacNNNtctaccaaggag |
| Mutagenesis codon 1338 | aaacgatacactNNNaccaaggaggtgctag |
| Mutagenesis codon 1339 | cgatacacttctNNNaaggaggtgctagac |
| Mutagenesis codon 1340 | gatacacttctaccNNNgaggtgctagac |
| Mutagenesis codon 1341 | acttctaccaagNNNgtgctagacgcga |
| Mutagenesis codon 1342 | tctaccaaggagNNNctagacgcgacactgat |
| Mutagenesis codon 1343 | accaaggaggtgNNNgacgcgacactg |
| Mutagenesis codon 1344 | aaggaggtgctaNNNgcgacactgattca |
| Mutagenesis codon 1345 | gaggtgctagacNNNacactgattcaccaat |
| Mutagenesis codon 1346 | gtgctagacgcgNNNctgattcaccaat |
| Mutagenesis codon 1347 | gctagacgcgacaNNNattcaccaatcc |
| Mutagenesis codon 1348 | gacgcgacactgNNNcaccaatccatca |
| Mutagenesis codon 1349 | acgcgacactgattNNNcaatccatcacg |
| Mutagenesis codon 1350 | gcgacactgattcacNNNtccatcacgggattat |
| Mutagenesis codon 1351 | acgcgacactgattcaccaaNNNatcacgggatta |
| Mutagenesis codon 1352 | gcgacactgattcaccaatccNNNacgggattatat |
| Mutagenesis codon 1353 | caccaatccatcNNNggattatatgaaactcgg |
| Mutagenesis codon 1354 | ccaatccatcacgNNNttatatgaaactcggataga |
| Mutagenesis codon 1355 | tccatcacgggaNNNtatgaaactcggata |
| Mutagenesis codon 1356 | catcacgggattaNNNgaaactcggataga |
| Mutagenesis codon 1357 | cacgggattatatNNNactcggatagatttg |
| Mutagenesis codon 1358 | ccatcacgggattatatgaaNNNcggatagatttg |
| Mutagenesis codon 1359 | cacgggattatatgaaactNNNatagatttgtcacagc |
| Mutagenesis codon 1360 | gggattatatgaaactcggNNNgatttgtcacag |
| Mutagenesis codon 1361 | gaaactcggataNNNttgtcacagcttggg |
| Mutagenesis codon 1362 | actcggatagatNNNtcacagcttgggggtg |
| Mutagenesis codon 1363 | ctcggatagatttgNNNcagcttgggggt |
| Mutagenesis codon 1364 | ctcggatagatttgtcaNNNcttgggggtgac |
| Mutagenesis codon 1365 | tcggatagatttgtcacagNNNgggggtgacatg |
| Mutagenesis codon 1366 | ttgtcacagcttNNNggtgacatgcatcc |
| Mutagenesis codon 1367 | gtcacagcttgggNNNgacatgcatccg |
| Mutagenesis codon 1368 | acagcttgggggtNNNatgcatccgggac |

**S4 Table. Primers used for SMOOT reactions and PacBio library preparation.**
