# Supplementary material for: SMOOT libraries and phage-induced directed evolution of Cas9 to engineer reduced off-target activity
Source: PLoS One. 2020 Apr 16;15(4):e0231716. doi: 10.1371/journal.pone.0231716 (PMC7161989; doi:10.1371/journal.pone.0231716)
Supplement: S6 Table — (DOCX) [file pone.0231716.s015.docx]

| Component | Volume |
| --- | --- |
| Template | 1 µL (100ng) |
| BS60 | 2.5 µL of 10 µM |
| BS61 | 2.5 µL of 10 µM |
| dH2O | 19 µL |
| 2X MM Phu HF | 25 µL |

|  | Temperature | Time |
| --- | --- | --- |
|  | 98°C | 30 sec |
|  | 98°C | 10 sec |
| 30 Cycles | 55°C | 30 sec |
|  | 72°C | 3 min |
|  | 72°C | 10 min |
|  | 4°C | Infin. |

**S6 Table.** **Reaction conditions for the PCR to prepare for PacBio sequencing.**
